# Emergent Ascomycetes in Viticulture: An Interdisciplinary Overview

**DOI:** 10.3389/fpls.2019.01394

**Published:** 2019-11-22

**Authors:** Carlotta Pirrello, Chiara Mizzotti, Tiago C. Tomazetti, Monica Colombo, Paola Bettinelli, Daniele Prodorutti, Elisa Peressotti, Luca Zulini, Marco Stefanini, Gino Angeli, Simona Masiero, Leocir J. Welter, Ludger Hausmann, Silvia Vezzulli

**Affiliations:** ^1^Research and Innovation Centre, Fondazione Edmund Mach, San Michele all’Adige, Italy; ^2^Department of Agricultural, Food, Environmental and Animal Sciences, University of Udine, Udine, Italy; ^3^Department of Biosciences, University of Milan, Milan, Italy; ^4^Center of Agricultural Sciences, Federal University of Santa Catarina, Rodovia Admar Gonzaga, Florianópolis, Brazil; ^5^Technology Transfer Centre, Fondazione Edmund Mach, San Michele all’Adige, Italy; ^6^Department of Natural and Social Sciences, Federal University of Santa Catarina, Campus of Curitibanos, Rodovia Ulysses Gaboardi, Curitibanos, Brazil; ^7^Julius Kühn Institute (JKI), Institute for Grapevine Breeding Geilweilerhof, Siebeldingen, Germany

**Keywords:** anthracnose, black rot, disease symptom phenotyping, genetic diversity, grapevine, powdery mildew, disease resistance loci, transcriptomics

## Abstract

The reduction of pesticide usage is a current imperative and the implementation of sustainable viticulture is an urgent necessity. A potential solution, which is being increasingly adopted, is offered by the use of grapevine cultivars resistant to its main pathogenic threats. This, however, has contributed to changes in defense strategies resulting in the occurrence of secondary diseases, which were previously controlled. Concomitantly, the ongoing climate crisis is contributing to destabilizing the increasingly dynamic viticultural context. In this review, we explore the available knowledge on three Ascomycetes which are considered emergent and causal agents of powdery mildew, black rot and anthracnose. We also aim to provide a survey on methods for phenotyping disease symptoms in fields, greenhouse and lab conditions, and for disease control underlying the insurgence of pathogen resistance to fungicide. Thus, we discuss fungal genetic variability, highlighting the usage and development of molecular markers and barcoding, coupled with genome sequencing. Moreover, we extensively report on the current knowledge available on grapevine-ascomycete interactions, as well as the mechanisms developed by the host to counteract the attack. Indeed, to better understand these resistance mechanisms, it is relevant to identify pathogen effectors which are involved in the infection process and how grapevine resistance genes function and impact the downstream cascade. Dealing with such a wealth of information on both pathogens and the host, the horizon is now represented by multidisciplinary approaches, combining traditional and innovative methods of cultivation. This will support the translation from theory to practice, in an attempt to understand biology very deeply and manage the spread of these Ascomycetes.

## Introduction

The earliest evidence of viticulture, namely grapevine cultivation and winemaking, was found in Iran, dating back to 7,400-7,000 B.C. (McGovern, 2004). Among the 60 hybridizing species (2*n* = 38) belonging to the *Vitis* genus, the Eurasian grapevine (*Vitis vinifera* L.) is the most extensively cultivated and of renowned worldwide economic importance, being used for the production of high quality wines, table grapes and raisins (Olmo, 1979). With the exception of a few recently explored *V. vinifera* accessions coming from the Caucasian cradle of grapevine domestication (e.g. Toffolatti et al., 2018), *V. vinifera* cultivars are generally highly susceptible to most fungal diseases, such as downy mildew, grey mold, powdery mildew (PM), black rot (BR), and anthracnose (AN) (Olmo, 1971). For this reason, the main strategy to prevent yield losses due to biotic adversities is the application of fungicide which is necessary to control the causal agents with inevitable negative impact on humans, animals and environment. Around 68,000 tons of fungicides per year are used in Europe to manage grapevine diseases, i.e. 65% of all fungicides used in agriculture, though viticulture encompasses only 4% of the arable land available in the EU (Muthmann and Nardin, 2007). Forecasts predict large increases in this trend, especially in viticulture, consistent with the worldwide data available (FAO, 2016).

**Figure 1:**
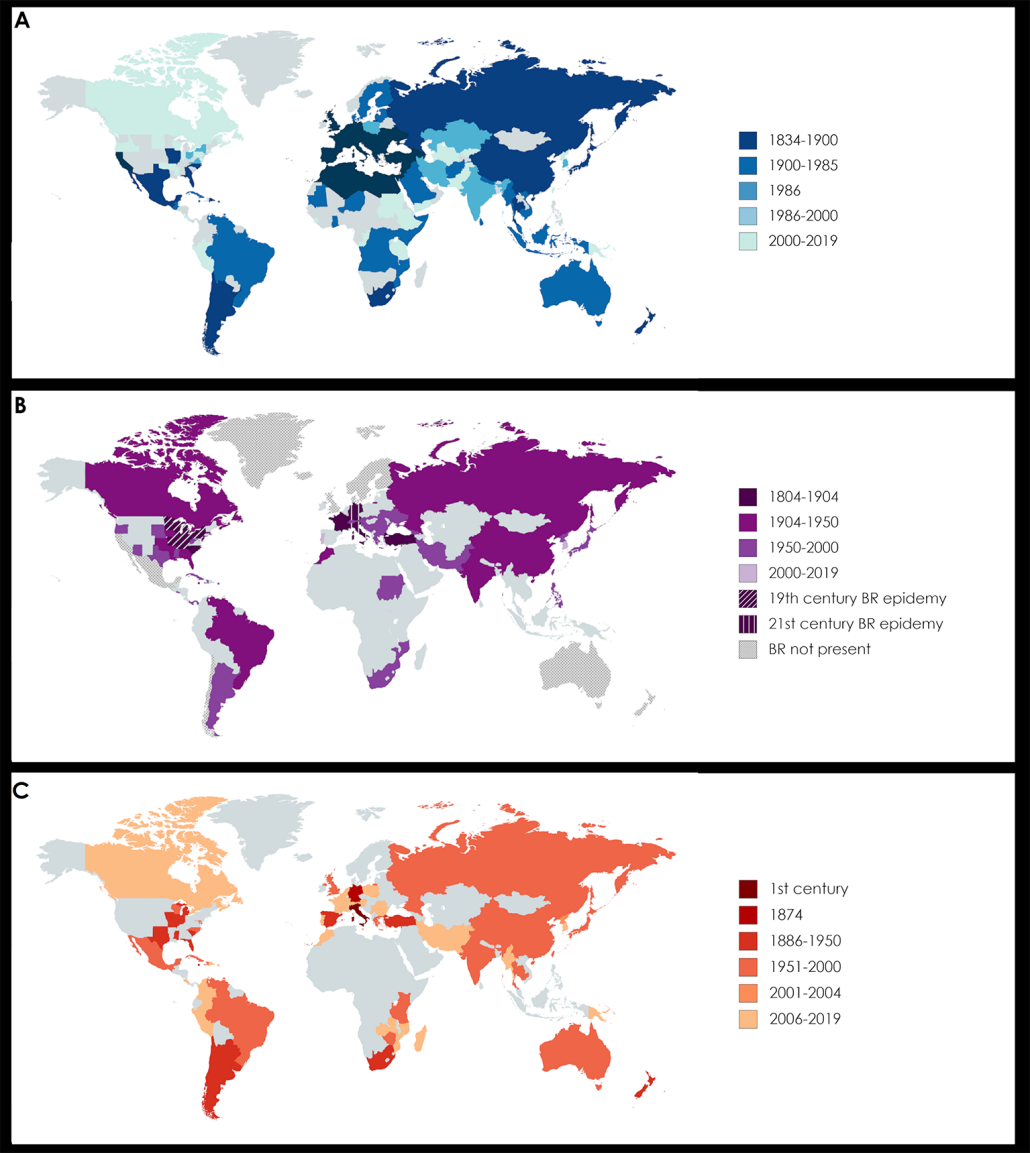
Worldwide diffusion of powdery mildew (PM, panel A), black rot (BR, panel B), and anthracnose (AN, panel C). The relative bibliographic sources are reported in **Table S1**. **(A)** PM was first reported in northeastern America in 1834 by Schweinitz. In 1845 it was introduced in Europe and less than 10 years later was affecting all the wine producing country of the Mediterranean region. In 1986, Amano published an outstanding review listing the countries all over the world where fungi causing PM were present at that time in relation with their specific plant hosts, including grapevine. Today PM can be considered a “worldwide grapevine disease”, since it afflicts vineyards all over the world. **(B)** BR is native of northeastern America. In 1804 it was noticed in Dufour’s vineyard (Kentucky) and it became epidemic in the second half of the 19th century in all the Great Lake Region, where the entire yield in many fields was lost. The first occurrence in Europe was recorded in 1885 by Viala and Ravaz in Southern France, then it spread all around the world, although without a huge economic impact. In 1989, BR showed an increasing presence in Switzerland, but it was in the 21th century that a second outbreak afflicted Europe, starting from Germany, where the economic losses were severe, to the Alps area (Ticino, Switzerland; Friuli and Veneto, Italy), Hungary and Romania. Regions with an unfavourable climate, as Scandinavia for cold and Mexico for dry weather, are considered BR “free” today. Interestingly, also in Australasia and Chile BR remains absent. **(C)** AN is considered one of the oldest known plant disease, since reference to it were reported in ancient Rome by Theophrastus (in *De causis plantarum*) and by Pliny the Elder (in *Naturalis historia*), dating back to the first century of the Christian era. Its European origin was also confirmed by the first report of the modern era in 1874 by De Bary, in Germany. AN diffusion was not alarming until its arrival in Tropical areas, such as South America. Nowadays AN is again becoming a threat in Europe.

A useful strategy to reduce the impact of pesticides towards a sustainable viticulture relies on breeding, by introducing resistance traits from wild species into domesticated varieties. Therefore, the change to these current varieties (cultivars) is now strongly advised. After initial difficulties, due to considerations regarding the quality of the wine produced with these varieties, resistant cultivars have recently been allowed by the EU Commission for the production of *PDO* (*Protected Denomination of Origin*) Dons wines in Denmark (Implementing Regulation 2018/606). In the last decade, it has been reported that the cultivation of new varieties resistant to downy mildew and PM, whose management needs less copper and sulphur-based treatments, favored BR diffusion (Harms et al., 2005; Töpfer et al., 2011). In fact, most cultivars which exhibit adequate resistance against mildews are highly susceptible to BR (Harms et al., 2005). In addition, considering BR is originally native to northern America, the appearance of BR symptoms is ongoing in a previously BR-free area (CABI, Crop Protection Compendium, 2018) (**Figure 1B**). Analogously, AN is sometimes problematic on highly susceptible interspecific cultivars, which typically receive only modest fungicide programs to control other diseases (Wilcox et al., 2017).

The transition to production of resistant cultivars, however, needs to be framed within the current climatic challenges. The two main players involved in the climate crises are temperature (global warming) and precipitation (including extreme phenomena) (Higgins and Scheiter, 2012). Besides having already extremely impacted natural phenology by limiting yields through drought and spring-frost damages, dramatic changes in climate will result in the accelerated reproductive cycles of biological organisms, destabilizing even further the precarious equilibrium among pathogens, pests and hosts (Salinari et al., 2006; Caffarra et al., 2012). Global surveys to identify the most relevant diseases and pests in many grape-growing regions worldwide provided preliminary results which allowed for the determination of the distribution of diseases by 2050 as a function of agroclimatic indicators. Upon these recent investigations, PM derived from northern America (**Figure 1A**), and AN which originated in Europe (**Figure 1C**), were also recently discovered in extremely diverse climatic conditions, including temperate regions with high rainfall, especially during spring months (Bregaglio et al., 2013; Bois et al., 2017; Wilcox et al., 2017).

Finally, in this challenging and dynamic viticultural context, we are witnessing the emergence of fungal diseases caused by Ascomycetes. The term “emergent disease” in this case is not to be interpreted in the strict sense of phytosanitary emergencies, but refers to diseases whose causative agents are already known, and for which control plans exist. These diseases however have manifested themselves or already conquered new regions, due to the recovery or the onset of favorable conditions, representing a real threat to worldwide viticulture.

## Disease Description

### Disease Symptom Assessment

#### Powdery Mildew

**Figure 2:**
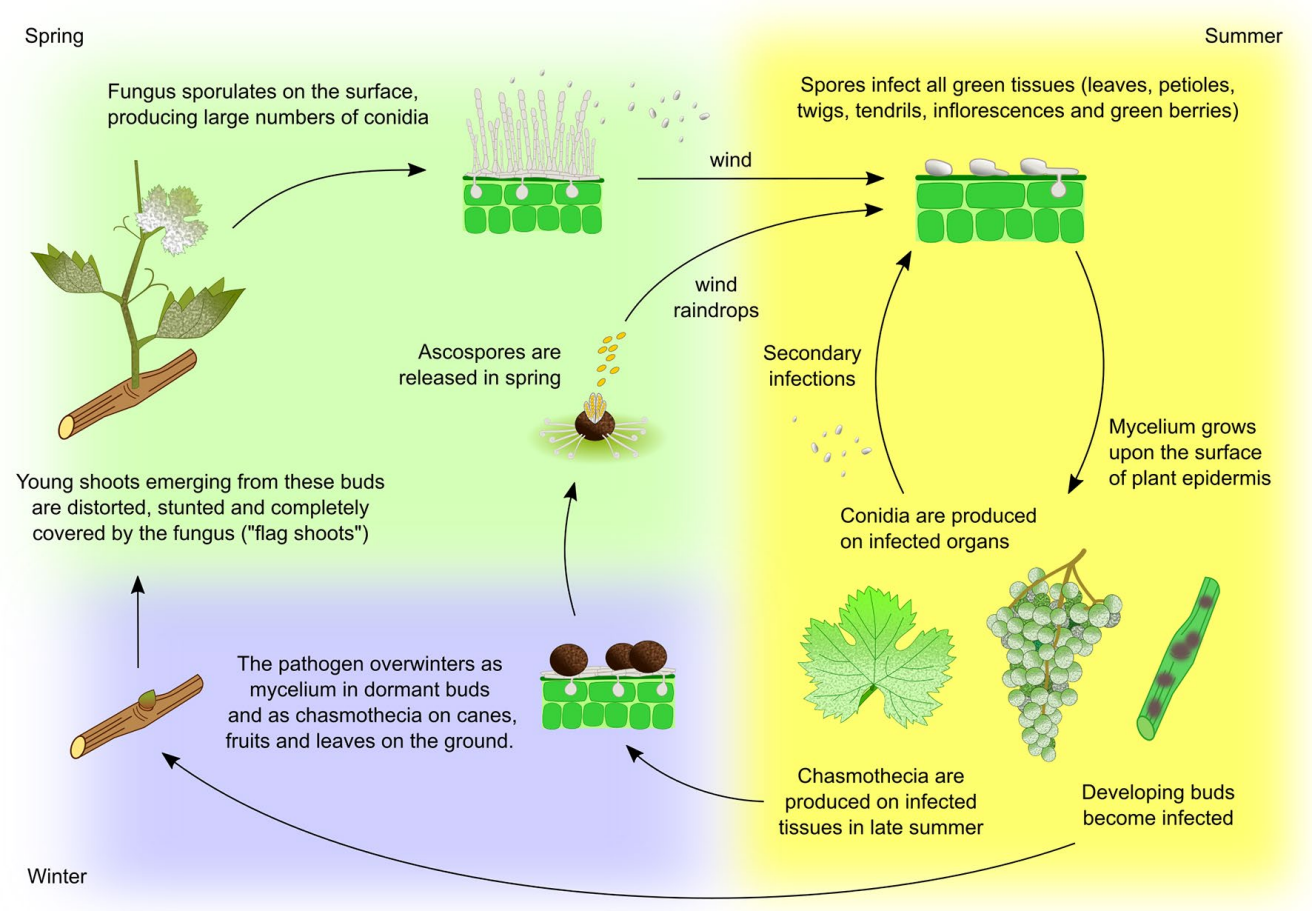
*Erysiphe necator* life cycle. Two overwintering strategies have been observed in *E. necator*. In areas with relatively mild winters, the fungus commonly overwinters as mycelium on leaf primordia within dormant buds. In the following spring mycelium activity resumes, resulting in the production of heavily infected and deformed shoots, called ‘flag shoots’. The fungus sporulates on these shoots, producing a large number of conidia that are carried by the wind to healthy plant tissues. Alternatively, the fungus can overwinter as chasmothecium (syn. cleistothecium, a former term for this structure that is still widely used) in bark, on canes, leftover fruit, and on leaves on the ground. Chasmothecia form on the surface of heavily diseased tissues from mid-summer to autumn. During spring rainfall the chasmothecia open and release ascospores, which are spread by wind or raindrops to infect the lower leaves near where the chasmothecia have overwintered. Although free water is necessary to release ascospores, continued wetness is not required for subsequent spore germination and infection. At each new infection site, conidia and ascospores germinate and form an appressorium. From its lower surface a penetration peg develops, piercing the cuticle and entering through an epidermal cell where a haustorium is formed. Mycelium grows upon the surface of the plant epidermis and new conidia are produced within a few days, completing the cycle. Repetition of this cycle continues throughout the growing season resulting in a rapid increase in disease incidence (Wilcox et al., 2017).

The causative agent of PM is the biotroph (obligate parasite) *Erysiphe necator* Schw. (asexual morph *Oidium tuckeri* Berk.) (**Figure 2**). PM is recognized by the appearance of a whitish-gray dusty layer on the grape which is caused by the spreading of mycelia and conidia onto green tissues (Pearson and Gadoury, 1992). Biological assays for the assessment of disease symptoms are fundamental to shed light onto host-pathogen interactions; conveniently, these can be carried out by observations in the field (e.g. Li, 1993; Wang et al., 1995; Pap et al., 2016), in greenhouses (e.g. Li, 1993; Amrine et al., 2015; Pap et al., 2016; Pessina et al., 2016), and *ex vivo* (e.g. Li, 1993; Wang et al., 1995; Staudt, 1997; Pap et al., 2016; Pessina et al., 2016).

**Figure 3:**
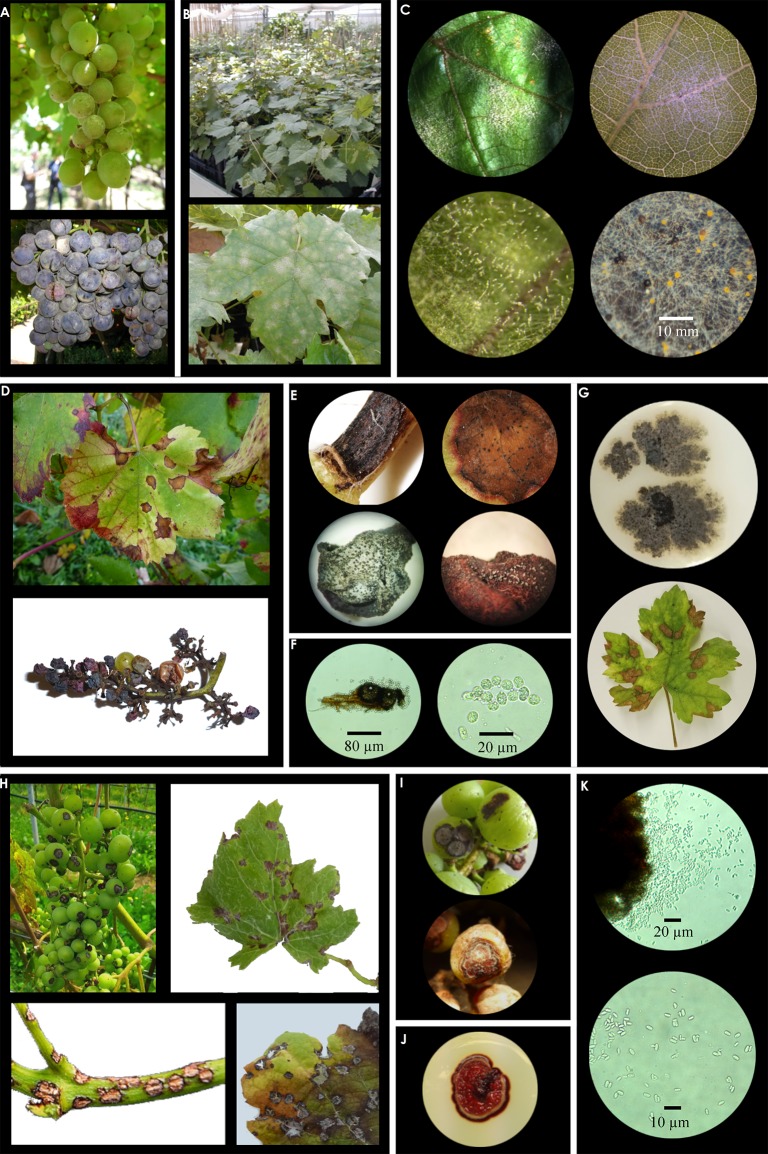
Fungal morphological characteristics and symptoms of powdery mildew (PM, panel **A**-**C**), black rot (BR, panel **D**-**G**), and anthracnose (AN, panel **H**-**K**). **(A)** PM on grapes (field). **(B)** PM on leaves (greenhouse). **(C)** PM leaf disc infection under different magnification (above); conidiophores and conidia on a leaf surface (below-left); mature (black) and immature (yellow) chasmothecia (below-right). **(D)** BR field symptoms on leaves and grape cluster. **(E)** *G. bidwellii* pycnidia on petiole and leaf (above), on berry with detail of cirri development under humid conditions (below). **(F)** Detail of *G. bidwellii* pycnidia and conidia under different magnification. **(G)** *G. bidwellii* isolate growing on culture media (above) and leaf symptoms after artificial infection (below). **(H)** AN field symptoms on a grape cluster, leaf and young shoot, along with detail about the typical “shot-hole” lesions on old infected leaf. **(I)** AN symptom details on berries. **(J)** *E. ampelina* colony on culture media. **(K)** *E. ampelina* acervulus releasing conidia and detail of conidia.

In the field, observations are carried out in mid-summer, when the symptoms are more evident and it is possible to estimate the occurrence of the pathogen in vineyards treated and not-treated with fungicide (Li, 1993; Wang et al., 1995). Symptoms can be monitored studying artificial infections (inoculations) using amplified conidia (Pap et al., 2016). To standardize results, the age of the leaf must be recorded, since older leaves display ontogenic resistance (Gadoury et al., 2012). In a greenhouse setting, different strategies can be adopted: for instance Li (1993) and Pap et al. (2016) inoculated potted plants by spraying them with a suspension of *E. necator* conidia in aqueous and Tween solution using 5 × 10^5^ conidia/ml and 0.7×10^5^ conidia/ml respectively. Pessina et al. (2016) brushed the adaxial surface of older leaves with young leaves carrying *E. necator* sporulation. Under these growing conditions, disease severity can be assessed within 3 to 21 days post-inoculation (dpi). *Ex vivo* pathogenesis assays are conducted using detached leaves or leaf disks and different inoculation strategies have been used. Péros et al. (2006b) tested three different spots of inoculum on detached leaves, using a glass needle to transport 20-60 conidia a time. Alternatively, Staudt (1997) brushed leaf disks with conidia from the mycelium of *E. necator*, while Miclot et al. (2012) developed a ventilation method. Wang et al. (1995) and Pap et al. (2016) successfully sprayed conidia suspension on detached leaves and foliar disks (**Figures 3A–C**).

In these studies, symptom assessment was valued qualitatively–counting the ratio of organs infected over the healthy ones–and quantitatively–by measuring the percentage of organ surface affected by symptoms with respect to the total surface. Miclot et al. (2012) compared three different inoculation techniques to assess the best method for quantitative analysis of PM resistance in grapevine. Starting from dry (Cartolaro and Steva, 1990), wet (Yamamoto et al., 2000) and drop (Moyer et al., 2010) inoculations, they determined a semi-quantitative index, which integrates pathogen sporulation and mycelium growth values. To normalize their results, data were converted into values according to the OIV 455 descriptor (Organisation Internationale de la Vigne et du Vin, 2009). This system relies on a rating of 1 to 9, where 1 represents the highest sporulated surface area (total susceptibility), 3 represents a strong infection, 5 indicates a medium infection, 7 a weak infection and 9 indicate that no symptoms are recognized (completely resistant). Within the EPPO (European and Mediterranean Plant Protection Organization, 2001) code, the two indicated parameters of quality (disease incidence) and quantity (disease severity) are available for symptom assessment. Improved accuracy in symptom assessment can be achieved through histochemical staining and microscopic analysis. Light microscopy can distinguish hyphae, appressoria, conidia and conidiophores, while haustoria cannot be monitored (Cadle-Davidson et al., 2010). Also confocal scanning electron microscopy (SEM) and low-temperature scanning electron microscopy (LTSEM) have been used to study *E. necator* (Carver et al., 1994; Cadle-Davidson et al., 2010; Gadoury et al., 2012; Ramming et al., 2012; Gao et al., 2016). Stainings successfully employed are: i) Coomassie blue, to monitor conidium germination and infiltration into host cells (Ramming et al., 2011; Ramming et al., 2012), ii) Trypan blue, to label the plant dead cells, following conidium germination, hyphae growth, and conidiophore emergence (Gao et al., 2016); iii) Aniline blue, to follow the infection using bright microscopy (Fekete et al., 2009; Gao et al., 2012a) as well as to localise the spores using fluorescence (Vanacker et al., 2000; Pessina et al., 2016).

#### Black Rot

The causal agent, *Guignardia bidwellii* (Ellis) Viala & Ravaz, is a hemibiotrophic pathogen [asexual morph *Phyllosticta ampelicida* (Engelm.) Aa]. BR can attack all the herbaceous expanding organs of the plant (leaves, shoots, tendrils, petioles and berries), with young shoots and fruits being extremely sensitive (Kuo and Hoch, 1996). The infection is characterized by a first symptomless phase and a second necrotic and damaging phase (Luttrell, 1974; Kuo and Hoch, 1996). On the adaxial surface of the leaves, the fungus causes the appearance of small circular spots that evolve into light brown lesions with darker borders. The central portion of the spot turns necrotic and pycnidia become visible as small black dots. On the fruits, the first occurrence is the appearance of small whitish dots that rapidly expand concentrically around the berry, forming a brown patch. Later, darker pycnidia develop as the berries rot and shrink, turning into black mummies (Ramsdell and Milholland, 1988).

**Figure 4:**
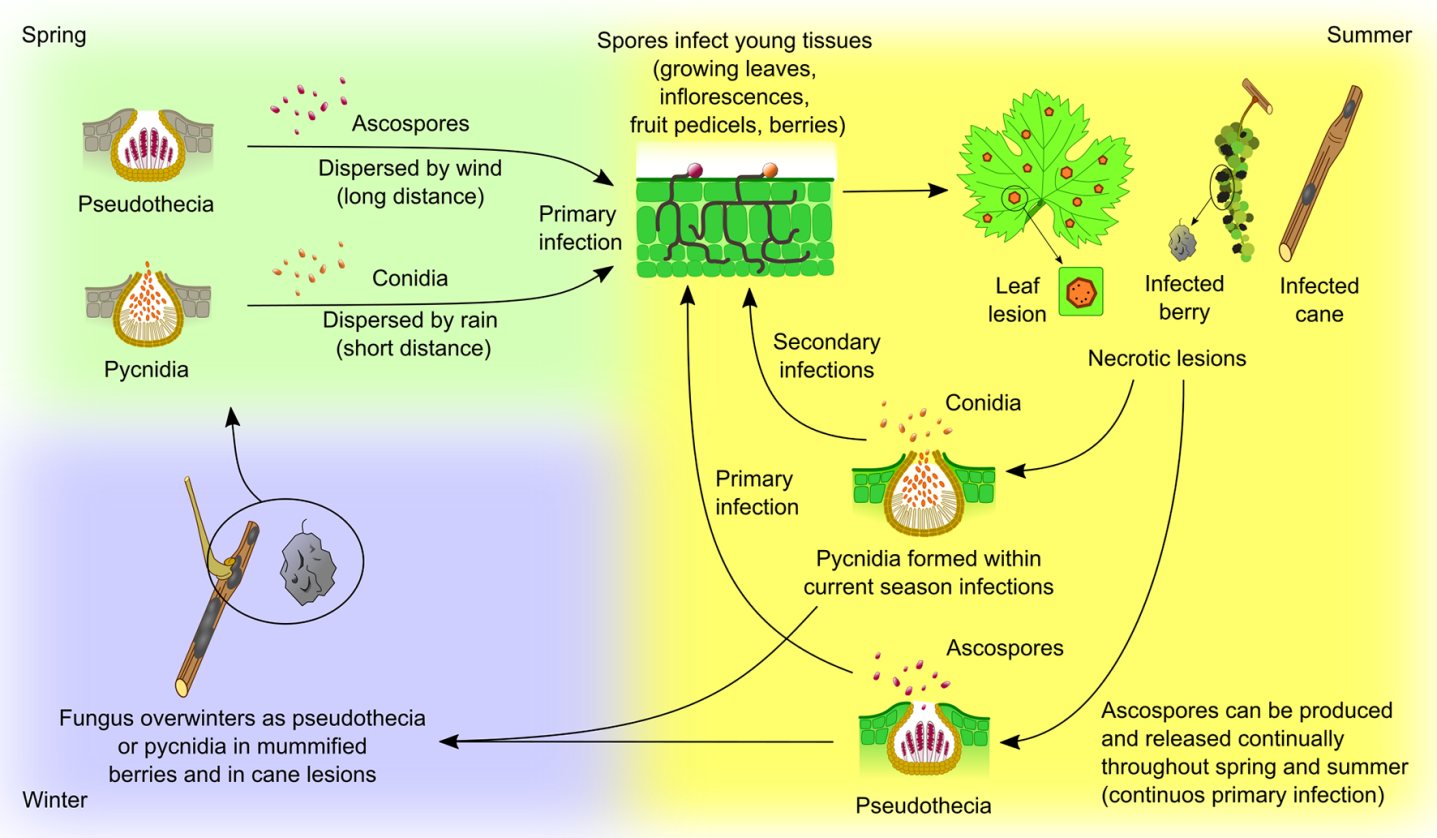
*Guignardia bidwellii* life cycle. The fungus overwinters in mummified berries, retained on the vine or fallen to the ground, and on infected canes. Berry mummies host both pseudothecia, containing asci with ascospores, and pycnidia, with conidia, while canes and tendrils host pycnidia. Lesions capable of producing conidia can persist in the wood for at least two years. In spring, ascospores and conidia are released when pseudothecia and pycnidia become thoroughly wet; infection is therefore favored by frequent rainfall as the spores need water to be released and to germinate. Ascospores released from mummified berries are the most common form of primary inoculum. They are ejected actively from the asci during rainfall and are dispersed by wind currents (long distance dispersion). On the contrary, conidia are exuded from the pycnidium in a white, mucilaginous cirrus from which they can be splashed away by rain (short distance dispersion). Primary infection from ascospores or conidia takes place on young, rapidly growing green tissues (growing leaves, inflorescences, fruit pedicels, berries). Adult leaves and ripe fruits, that have become fully expanded, are not susceptible to infection. Pycnidia are produced rapidly within the necrotic lesions found on leaves, shoots and berries, and, once mature and dampened by rain, they release the conidia which serve as secondary inoculum throughout the season. When the weather is moist, ascospores may be produced and released continually throughout spring and summer from mummies retained in the canopy, providing continuous primary infection, although most of them are discharged in the spring. In late summer, the sexual cycle initiates on infected berries and pseudothecia are formed (Wilcox et al., 2017).

BR is a polycyclic disease with repeated cycles of primary and secondary infections (**Figure 4**). As reviewed by Onesti (2015), the fruiting bodies bearing the ascospores (ascocarps) have been referred to as either perithecia or pseudothecia, since the wall of stromal tissue of the pseudothecium can be confused with the wall formed by the peridium in a simple perithecium; hence the term pseudothecia will be used throughout the manuscript. Ascospores and conidia are both released during precipitation events: Ferrin and Ramsdell (1978) found a positive correlation between BR infections and the magnitude of rainfall, the number of events and their duration, and the persistence of water on leaves. These observations were also confirmed recently (Onesti et al., 2018). As ascospores and conidia are both sensitive to desiccation, BR is not a prevalent disease in dry climates (Ferrin, 1976; Spotts, 1976; Ferrin and Ramsdell, 1977; Spotts, 1977), however, in the field, spores can germinate even after dry summer periods (Ferrin and Ramsdell, 1978; Besselat and Bouchet, 1984; Hoffman et al., 2004) and pycnidia can produce conidia even after three months of low humidity (Onesti et al., 2017a). Field experiments demonstrated that release dynamics of both types of spores are conserved: conidia are released approximatively at budburst stage, while for ascospores it occurs two weeks later; in the course of the fruiting season, when berries are pea-sized, both types reach their maximum (Onesti et al., 2018). For the ascospores, the first peak is registered between flower pre-blooming to anthesis (Ferrin and Ramsdell, 1977).

The most sensitive period for direct infection on berries is after flowering, from fruit onset to the beginning of bunch closure. A field-trial of artificial infection in a *V. labrusca* ‘Concord’ vineyard revealed that plants infected between mid-bloom and fruit onset present the highest lesion number on leaves and the greatest berry infection (Ferrin and Ramsdell, 1978). These data were confirmed also in greenhouse conditions (Kuo and Hoch, 1996) (**Figures 3D–G**). Moreover, berries can be susceptible for a longer period to *G. bidwellii* compared to other relevant pathogens, for instance *E. necator* (Gee et al., 2008). Since the duration of phenological stages can differ among cultivars, windows of susceptibility (Hoffman et al., 2002) and the number of days after infection for symptom appearance (Roznik et al., 2017) are cultivar-specific.

Ontogenic resistance of plant hosts to fungi is widely documented (Populer, 1978), and for *G. bidwellii* it might be a defense mechanism able to counteract the pathogen, rather than a reduction in the germination ability of the fungus in older tissues (Kuo and Hoch, 1996; Molitor and Berkelmann-Löhnertz, 2011). Notably, the extent of the infection negatively correlates with leaf size, since smaller leaves display greater infected surface than larger, older leaves (Luttrell, 1948; Jabco et al., 1985). Kuo and Hoch (1996) suggest “expanding” and “non-expanding” organs to better describe the resistance displayed by aging tissue. With regards to the duration of pathogen incubation, Molitor et al. (2012) do not report differences on leaves of different ages. In contrast, at grape level ontogenic resistance under field conditions is responsible for the decrease of the number of infected berries and the increases of the incubation time (Hoffman and Wilcox, 2002; Hoffman et al., 2002). Roznik et al. (2017) suggested that the developmental stage of plant tissue is crucial for the results of the artificial tests, which could explain the incongruence of research group results. Finally, this issue is tightly linked to different inoculation conditions since constant temperature shortens the incubation period rather than fluctuations (Spotts, 1980), the required incubation time on leaves (Spotts, 1977; Molitor et al., 2012) and shoots (Northover, 2008), and the release of ascospores are temperature dependent (Rossi et al., 2015).

Cross-inoculation experiments between fungi collected from different species (*Parthenocissus tricuspidata, P. quinquefolia, Muscadinia rotundifolia, V. labrusca, V. bourquina, V. vinifera*) demonstrated that *G. bidwellii* includes three *formae speciales (f.sp.)*, named *parthenocissi, muscadinii* and *euvitis*, with different degree of pathogenicity on the different hosts (Luttrell, 1946; Luttrell, 1948). Greenhouse assays were later performed (Jabco et al., 1985) with *f.sp euvitis* and *muscadinii* exploring leaf and petiole infection to assess the specific resistance response of four grapevine classes: *vinifera*, French and American hybrids, *rotundifolia*. The latter class showed a medium to high susceptibility to f.sp. *muscadinii*, while it resulted highly resistant to f.sp. *euvitis*; in contrast, the other three classes displayed high to medium resistance to f.sp. *muscadinii*, but medium to high susceptibility to f.sp. *euvitis*, with *vinifera* class developping larger lesions. *In vitro* cultures were also used to clarify the life cycle of the homotallic *G. bidwellii* (Jailloux, 1992). Light triggers pseudothecia maturation and differentiation, but during the first phase of mycelial growth, it inhibits pseudothecial growth and it induces pycnidia production. The optimal temperature for mycelial growth and pycnidia development (25°C) is also adequate for the first phase of pseudothecial growth, however lower temperatures are necessary for the maturation and differentiation of the ascospores. Temperature was also found to influence the dynamic and the number of pycnidia production and conidial germination (Onesti et al., 2017b).

#### Anthracnose

**Figure 5:**
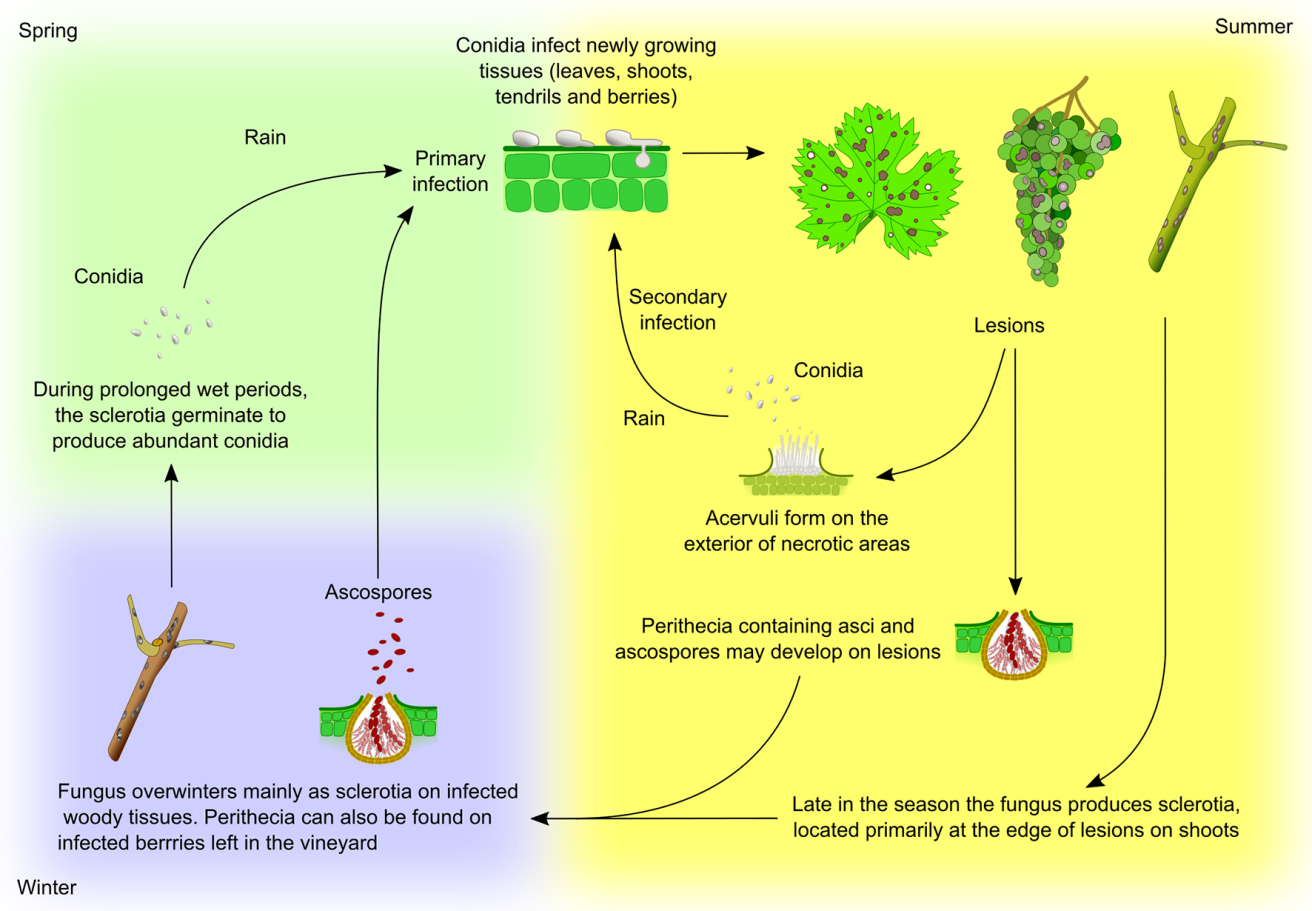
*Elsinoë ampelina* life cycle. The fungus overwinters mainly in infected canes as sclerotia, dense mycelial masses which are formed in autumn at the edge of lesions on shoots. In spring, sclerotia develop stromata on which, under humid conditions, produce abundant conidia. These conidia are then carried by rain or free water to young, rapidly growing green tissues (leaves, shoots, tendrils and young berries) where they germinate causing the primary infection. Conidia are by far the most important source of primary inoculum in spring. The fungus can also overwinter on infected berries, left on the vine or vineyard floor, as conidia or ascospores, which may also cause primary infections. Once the disease is established, the pathogen produces lesions upon which acervuli form and produce conidia which, dispersed by raindrops, serve as secondary source of inoculum for the rest of the growing season. During periods of humid weather, conidia can be released the entire spring and summer providing continuous infection. Heavy rainfall and warm temperatures are ideal for disease development and spread. At times pseudothecia, containing asci and ascospores, develop on the lesions (Wilcox et al., 2017).

The causal agent of AN is commonly attributed to the hemibiotrophic *Elsinoë ampelina* Shear, whose asexual morph is *Sphaceloma ampelinum* de Bary (**Figure 5**). Moreover, some authors reported also *Colletotrichum* spp., another ascomycete, associated with the disease symptoms, such as *C. nymphaeae*, *C. fructicola* and *C. gloeosporioides* (Sawant et al., 2012; Liu et al., 2016; Guginski-Piva et al., 2018), *C. goditiae* (Baroncelli et al., 2014; Zapparata et al., 2017) and a complex of species that were grouped as *C. viniferum* (Yan et al., 2015). However, according to another author, *Colletotrichum* spp. is the causal agent of “ripe rot” in grapevine (Huang, 2016; Wilcox et al., 2017). Therefore, this association between pathogen and disease is still controversial.

The symptoms reported for the disease are similar, regardless of the causal agent. The pathogen attacks all aerial green parts of the plant, including fruit stems, leaves, petioles, tendrils, young shoots, and berries, however lesions of the pathogen are more common and distinctive on young shoots and berries (Magarey et al., 1993a) (**Figures 3H–K**). Structures of the pathogen are also found in dead tissues of the host, such as branches and fruits, making it difficult to eliminate the initial inoculum source of the pathogen (Magarey et al., 1993b). Infection on branches is recognizable by small circular reddish spots, afterwards the spots enlarge, forming a depression with gray center and rounded or angular edges, and eventually becomes surrounded by reddish brown or violet edges. Later on, the lesions may coalesce, killing the infected tissues. In some cases, slightly raised edges surrounding the lesions are also visible (Magarey et al., 1993a). On berries, the symptoms appear similar, initially, reddish circular spots appear, which evolve in size, and normally become slightly sunken. As spots grow, the center of the lesion turns whitish-gray, while edges assume a reddish-brown to black color. At this stage the lesion resembles a “bird’s eye”, hence the popular name for the disease (Magarey et al., 1993a; Jang et al., 2011). Young leaves are more susceptible to infection than older leaves. The initial lesions are small, circular and chlorotic. The lesions become larger with gray centers and brown to black margins with round or angular edges. The center of the lesions becomes dry and ash, and often drops out, forming a “shot hole” appearance (Magarey et al., 1993a). The size of the lesions may vary with the degree of resistance of the host genotype. While bigger and circular spots are reported for susceptible cultivars, smaller and with irregular shape lesions were observed in resistant cultivars (Kono et al., 2012) (**Figures 3H–K**).

The identification of resistance sources to AN has been a major task for grapevine breeders, especially in Brazil and China. Screenings for AN resistance in fields with ongoing natural infections have effectively been applied for genetic improvement goals (Fennell, 1948; Mortensen, 1981; Wang et al., 1998; Li et al., 2008). However, in the field the disease is highly influenced by the climatic conditions, requiring some years of evaluation to produce robust data. This, in addition to the perennial nature of the host, makes the analysis time consuming and costly.

Therefore, many authors focused their efforts on establishing alternative methodologies based on artificial infection with conidia on potted plants in greenhouses and on detached cane, detached leaf or leaf piece assays. For these purposes, *in vitro* production of conidia of *E. ampelina* proved to be a major challenge, due to its poor and unstable sporulation in culture. Even though Hopkins and Harris (2000) produced conidia from 3 to 4 week old cultures of *E. ampelina* maintained on potato dextrose agar (PDA), other researchers were unable to reproduce these results (Santos et al., 2018a; Santos et al., 2018b). In order to improve the yield of conidia, Yun et al. (2006) transferred the pathogen in Fries liquid medium and incubated it in a shaking incubator (140 rpm) at 28°C for 10 days. Cultures were then transferred to a V-8 juice agar medium and incubated at 28°C under a near ultraviolet lamp for two days to stimulate spore production. Kono et al. (2009) took a step forward and was the first to test the effect of culture conditions on conidia formation of *E. ampelina*. The study revealed the following three indispensable conditions for conidial production: (i) the density of colonies in pre-culture should be lesser than 2.5 colonies per cm^2^; (ii) depending on colony density, colonies should grow for 4 to 9 days on PDA; and (iii) grown colonies should be cultured in water with shaking (150 rpm) for 8 to 10 h in the dark at 24°C. Santos et al. (2018a) used a similar protocol. Conidial suspensions were obtained by placing *E. ampelina* cultures in rainwater and shaking at 200 rpm in darkness for 7 days to induce sporulation. Mortensen (1981) set up greenhouse screenings for resistance to *E. ampelina*, however the protocol was deemed not reliable; later Hopkins and Harris (2000) optimized greenhouse screening by misting the seedlings with a suspension containing 10^6^ conidia mL^-1^ and placing the inoculated seedlings in a moist chamber at 24°C for 48 hours, followed by 8 days on a greenhouse bench. However, consistent results were obtained at temperatures ranging from 20 to 28°C. Inoculations using conidia suspension were also performed on detached canes (Santos et al., 2018a), on detached leaves (Tharapreuksapong et al., 2009; Kono et al., 2012) and on leaf pieces (Poolsawat et al., 2012). After the inoculation, the tissues were maintained under high humidity at 25-27°C. The symptoms were assessed in variable times of incubation. The results obtained by these assays were consistent and were applied for pathogenicity analysis (Tharapreuksapong et al., 2009; Santos et al., 2018a), germplasm characterization (Kono et al., 2012) and genetic analysis (Tantasawat et al., 2012; Poolsawat et al., 2013).

As an alternative to artificial infection with conidia, Yun et al. (2006) developed a bioassay based on fungal cell-free culture filtrate (CFCF). In this case, the pathogen was cultivated in Fries medium for 21 days at 28°C, after then the CFCF was obtained by centrifugation and sterilized through ultra-filtration (0.2 µm). The CFCF was applied on wounded young leaves. The results demonstrated that the bioassay with culture filtrates of the pathogen were consistent with those from pathogen inoculation in the greenhouse and screening in the vineyard. The methodology was further validated by Yun et al. (2007) and applied for germplasm characterization (Jang et al., 2011; Louime et al., 2011) and genetic analysis (Kim et al., 2008).

### Powdery Mildew, Black Rot and Anthracnose Control Measures

Effective disease control encompasses the combination of sanitary as well as cultivation practices, the use of resistant or at least less susceptible grapevine varieties, the application of fungicides, and decision support systems (e.g. Wilcox, 2003; Hoffman et al., 2004; Molitor and Beyer, 2014). Cultivation and sanitation practices are essential to manage PM, BR and AN; for instance, adequate pruning and removal of leaves covering clusters provide conditions to reduce infections of PM. The removal of mummified bunches, infected canes and tendrils from the vineyard allows for the reduction of the primary inoculum of BR and AN, as well as covering mummies or infected berries on the ground by soil cultivation or mulching (Wilcox et al., 2017). Especially for BR, abandoned vineyards should be cleared since BR can build up high amounts of inoculum that can escape under favorable weather conditions and cause unexpected serious damages in the neighboring vineyards (Ullrich et al., 2009).

Due to the susceptibility of *V. vinifera* cultivars to PM, BR and AN, fungicide applications are necessary to control these diseases. In particular, thallus of *E. necator* develops almost completely outside of the infected tissues on the leaf and bunch surface, therefore the fungus is susceptible to topical applications of several contact active ingredients (Wilcox et al., 2017). Since the 19^th^ century, sulfur remains the most widely used fungicide, due to its low cost and protectant-curative action (Wilcox et al., 2017). The persistence of sulfur efficacy relies on the lack of resistance development, depending on its multi-site mechanism of action by direct contact and vapour phase: respiration inhibition, chelation of heavy metals needed for biochemical pathways, and disruption of protein function (Oliver and Hewitt, 2014). It causes the damage of cellular membrane followed by loss of water and therefore death of the fungus by dehydration. Other than sulfur, several single-site synthetic fungicides are effective against PM, including contact, translaminar and systemic products, with specifically targeted mechanisms of action. Among them, mitosis and cell division inhibitors (e.g. benzimidazoles) and cell membrane synthesis alteration *via* ergosterol biosynthesis inhibitors (e.g. triazoles); different mechanisms concern respiration chain inhibition *via* quinon inhibitors (e.g. strobilurines) or succinate dehydrogenase inhibitors; and signal transduction inhibition (e.g. azanaftalenes) (Oliver and Hewitt, 2014; Wilcox et al., 2017). Alternation of active ingredients with different modes of action is important to avoid the development of pathogen resistance, especially towards single-site systemic fungicides. Regular plant protection with chemical fungicides is the strategy of choice to control BR. High efficacy was observed with the fungicide classes of demethylation inhibitors (DMI), quinone outside inhibitors (QoI) and dithiocarbamates (Molitor and Beyer, 2014). Analogously to PM and BR, AN control relies on prophylactic fungicide applications. The first measure against the disease is to reduce the inoculum source by the elimination of cultivation remains and dormant spray of lime sulfur (Magarey et al., 1993b). The initial development of the fungus occurs outside the green tissues, resembling the conditions described for PM. For chemical control of the AN disease, multi-site active fungicides based on copper (e.g. copper hydroxide, copper oxychloride, cuprous oxide and copper sulfate) and on the chemical groups dithiocarbamate, phthalimide, phthalonitrile and anthraquinon may be employed. Single-site synthetic fungicides are also effective acting on cell division (e.g. thiophanate), on extracellular inhibitor of quinone (e.g. methoxy-acrylate) and on sterol biosynthesis (e.g. triazoles) (FRAC, 2019).

Due to the potential negative impacts of fungicide application, non-synthetic chemicals and organic control measures are also used to regulate these three diseases. For organic viticulture, applications of copper and sulfur are recommended, but generally they are less effective in comparison to the synthetic active compounds (e.g. Loskill et al., 2009; Wilcox et al., 2017). Nowadays, organic management against PM can rely, other than sulfur, on non-toxic substances such as botanical oils and inorganic salts, acting by contact with the fungal thallus (Wilcox et al., 2017). The application of *Ampelomyces quisqualis* (hyperparasite fungus) at the time of chasmothecia formation can help in reducing the overwintering inoculum of *E. necator* (Pertot et al., 2017). Concerning organic BR control, the application of copper is insufficient but in combination with sulfur showed a clearly higher effect to reduce the disease impact (Loskill et al., 2009). Moreover, first attempts with plant extracts from *Primula* roots and *Hedera helix*, containing saponine as active compound, showed clear effects in greenhouse experiments. However, under field conditions the activity was low due to the high water solubility (Koch et al., 2013). Consequently, in organic farming, it is crucial to integrate adequate sanitation, new varieties with genetic resistance or high tolerance, application of forecast models and web-based decision support systems (Molitor et al., 2016; Onesti et al., 2016) for the optimization of the fungicide spray regime. Regarding AN, plant extracts from *Chaetomium cupreum* and *C. globosum*, antagonistic microorganisms such as *Trichoderma harzianum*, *T. hamatum*, *Penicillium chrysogenum* and the natural antibiotic substances Rotiorinol, Chaetoglobosin-C and Trichotoxin A50 have been tested and proved to reduce AN incidence on leaves, shoots and grapes (Soytong et al., 2005). Bacterial antagonists are also cited for organic control, like *Bacillus* species strains TS-204 and TL-171 (Sawant et al., 2016).

### Ascomycete Resistance to Fungicide

To date, more than 50 modes of action have been identified for fungicides (FRAC, 2019); those ones that have a single-site mode of action are more problematic since resistance can rapidly evolve by a single mutation (Brent and Hollomon, 2007). Pathogens show cross resistance to compounds with the same mode of action but not to the other ones (Hollomon, 2015).

Among the widely employed fungicides used to control PM are the sterol DMI and QoI. *E. necator* resistance to DMI was reported in the 80s from California, Portugal and Australia (Ogawa et al., 1988; Gubler et al., 1996; Steva and Cazenave, 1996; Ypema et al., 1997; Savocchia et al., 1999). The DMI resistance is a multigenic trait, but with one major mechanism involving a single mutation in the gene *CYP51* coding for the cytochrome P450 lanosterol C-14α demethylase. Studies on DMI fungicide resistance revealed several possibilities to confer reduced sensitivity: (i) mutation of *CYP51*; (ii) overexpression of *CYP51*; (iii) overexpression of transporter coding for efflux pumps and (iv) other unknown mechanisms able to confer weak resistance (Délye et al., 1997a; Délye et al., 1998; Hamamoto et al., 2000; Schnabel and Jones, 2001; Hayashi et al., 2002; Lupetti et al., 2002; Corio-Costet et al., 2003; Stergiopoulos et al., 2003; Wyand and Brown, 2005; De Waard et al., 2006; Ma et al., 2006; Leroux et al., 2007; Luo et al., 2008; Cannon et al., 2009; Kretschmer et al., 2009; Sombardier et al., 2010; Leroux and Walker, 2011; Cools and Fraaije, 2013; Frenkel et al., 2015). *E. necator* is one of the first fungi for which it was demonstrated that a point mutation in *CYP51* is associated with DMI resistance. A mutation in codon 136 converts tyrosine (Y) to phenylalanine (F), reducing the sensitivity to the fungicide (Délye et al., 1997a). Moreover, a nucleotide substitution in position 1119 (A1119C) increases the *CYP51* expression causing a comparable lower sensitivity to the fungicide (Frenkel et al., 2015). QoI fungicides inhibit mitochondrial respiration by binding to the cytochrome bc1 enzyme complex (complex III) at the Qo site, blocking the electron transfer to cytochrome c1, and preventing the synthesis of adenosine-5′-triphosphate (ATP). Several point mutations in the *cytochrome b* (*CYTB*) gene confer QoI resistance (Gisi et al., 2002). *E. necator* resistance to QoI was initially described in the United States (Wilcox, 2005; Baudoin et al., 2008; Miles et al., 2012) and it is mainly associated with a point mutation in the codon 143 of *CYTB* that converts glycine (G) to alanine (A) (Bartlett et al., 2002; Ma and Michailides, 2005; Dufour et al., 2011). Recently, the emergence of *E. necator* resistance to other fungicides was reported, such as metrafenone, a benzophenone of which mode of action is still not known, and boscalid, a fungicide that inhibits the activity of the enzyme succinate dehydrogenase (Kunova et al., 2016; Cherrad et al., 2018).

*G. bidwellii* resistance to fungicide is not well studied even if the first report of DMI fungicide reduced sensitivities dates back to 1986 in France (Thind et al., 1986). Different field experiments demonstrated that DMI and QoI are almost 100% efficient in the BR control (Lafon et al., 1984; Ellis, 1986; Wilcox and Riegel, 1996; Wilcox and Riegel, 1997; Wilcox et al., 1999; Wilcox, 2000; Harms et al., 2005; Loskill et al., 2009; Tomoiaga and Comsa, 2010). Moreover, sequence analysis pinpoints that *G. bidwellii* has a low risk to generate QoI resistance (Miessner et al., 2011).

Fungicide resistance in *E. ampelina* has not yet been characterized. So far just one report describes the reduced sensitivity of the pathogen to carbendazim, a methyl benzimidazole carbamate fungicide, that inhibits microtubule assembly during mitosis (Deokate et al., 2002).

## Genetic Variability

### Molecular Marker Development

#### Erysiphe necator

Variations in the overwintering strategies have been observed in *E. necator* specimens in correlation to their geographic location. Given the scarcity of information about PM epidemiology, in the 90’s Délye and co-workers (Délye et al., 1997b; Délye and Corio-Costet, 1998) carried out a number of studies based on the use of RAPD (Random Amplified Polymorphic DNA) molecular markers, as well as mutagenized *CYP51* which encodes a eburicol 14α-demethylase, a highly conserved cytochrome P-450 enzyme essential for sterol biosynthesis (Délye et al., 1999). Analyzing the genetic variation among populations of *E. necator* from Europe, Asia, North-Africa and Australia, the existence of two main genetic groups was identified: the flag-shoot (A) and the ascospores (B) biotypes. In contrast, Cortesi et al. (2004) using the Inter-Simple Sequence Repeat (ISSR) markers did not observe any correlation between overwintering strategies and genetic groups. To better understand the distribution of the two biotypes, more in-depth research was devised. Some studies employed tagging of specific sequences by PCR (e.g. Brewer et al., 2011; Oliveira and Cunha, 2015), while others reported the use of dominant markers such Amplified Fragment Length Polymorphisms (AFLPs) (Núñez et al., 2006) and Random Amplification of Polymorphic DNA (RAPDs) (Péros et al., 2005). Codominant markers, i.e. Sequence Characterized Amplified Regions (SCARs), Random Fragment Length Polymorphisms (RFLPs), and Single Nucleotide Polymorphisms (SNPs), have been adopted on *CYP51* and *Entub* genes with the same aim (Evans et al., 1997; Stummer et al., 2000; Hajjeh et al., 2005; Amrani and Corio-Costet, 2006; Miazzi et al., 2008). More recently, Single Sequence Repeats (SSRs) were proven to be the most effective markers because of their high polymorphism, co-dominance and reproducibility. SSRs are widely used but their application in fungi was limited due to scarce sources of genetic diversity; moreover microsatellites are less abundant and present a reduced number of repeats in fungi (Dutech et al., 2007). One of the first attempts to use SSR markers to distinguish genetic groups of *E. necator* was made by Péros et al. (2006a). Wakefield et al. (2011) used cDNA-AFLPs to investigate the transcriptional changes during pathogenesis stages. Currently, the leading study on this topic is Frenkel et al. (2012) which developed microsatellite markers to investigate population structure of the causal agent of grapevine PM in North America, using 11 transcriptome-based microsatellites. According to these studies—despite a higher amount of genetic group A isolates being usually found early in the growing season, slightly giving away to group B over the course of the epidemics (Amrani and Corio-Costet, 2006; Miazzi et al., 2008)—the separation between the two biotypes is not dependent on the primary source of inoculum (Núñez et al., 2006). Moreover, in Europe the distribution of the two biotypes seems to be linked to the geographic location and host cultivar though in some cases both groups were detected in the same vineyard or even on the same plant (Amrani and Corio-Costet, 2006; Oliveira and Cunha, 2015).

#### Guignardia bidwellii

*G. bidwellii*-specific SSR markers appeared almost ten years later than *E. necator* markers. Only recently, Narduzzi-Wicht et al. (2014) developed 11 specific SSR markers, which were used to evaluate more than 1300 specimens, albeit without finding a relation between the species/cultivar of *Vitis* host, and the genotype of the infecting *G. bidwellii*. These 11 SSR markers were also used to analyze 37 strains of *G. bidwellii* and to assess the genetic variability of the fungus. This analysis revealed the presence of 56 haplotypes from 421 analyzed berries divided into four main subpopulations, pinpointing that the sexual reproduction is a crucial step in the progression of the epidemic in the vineyard (Rinaldi et al., 2017).

#### Elsinoë ampelina

The genus *Elsinoë* includes at least 75 species causing diseases on many plant hosts, including economically important crops. Hyun et al. (2001) performed a molecular characterization of several *Elsinoë* strains using RAPD markers, dominant markers with a limited application in genetic diversity studies. More recently, genome sequencing approaches allowed the revision of the fungus taxonomy, using both DNA sequences and published morphological data (Fan et al., 2017). As of today, codominant markers (SSRs or SNPs) are not available for genetic studies. Very little is known about *E. ampelina* genetic variability. First evidences of the pathogen variability were based on morphological characterization, which revealed polymorphism of colony size, colony color/appearance and conidial morphology (Poolsawat et al., 2009; Mathukorn, 2012). However, the morphological characteristics are too variable and do not necessarily reflect the genetic diversity (Poolsawat et al., 2009; Poolsawat et al., 2010). Therefore, molecular characterization is required to precisely differentiate the isolates. The use of the dominant RAPD markers confirmed the high pathogen diversity in Thailand (Tharapreuksapong et al., 2009; Poolsawat et al., 2010). It should be noted that, in this instance, genetically divergent isolates have shown different levels of pathogenicity. For example, Poolsawat et al. (2010) analysed five isolates, representing four genetically different groups, testing nine genotypes of grapevine. All isolates were pathogenic to susceptible genotypes: in particular, the host genotypes Wilcox321 and Illinois547-1 were highly resistant to all isolates. NY65.0550.04 was highly resistant to most isolates except Nk4-1. NY88.0517.01 and NY88.0507.01 were resistant only to some isolates and NY65.0551.05 was susceptible to most isolates except Nk5-1. These results suggest the presence of different resistance genes in the host and corresponding avirulent genes of the pathogen.

Within *Colletotrichum* spp., only *C. gloeosporioides* has 39 SSR markers identified (Penet et al., 2017) with a range of 2-29 alleles, enabling genetic diversity studies without the need of previous sequencing. However, there are no studies reporting the extensive use of these markers for isolate characterization. So far, the publications exploring *Colletotrichum* diversity and phylogeny involved the sequencing of genomic regions (Baroncelli et al., 2014; Guginski-Piva et al., 2018).

### Barcoding and Genome Sequencing

#### Erysiphe necator

The first internal transcribed spacer (ITS) of ribosomal gene sequence from *E. necator* was released in 1999 (Saenz and Taylor, 1999): this enabled the identification of primers unique to *E. necator* to differentiate this fungus from other causal agents of PMs (Falacy et al., 2007). Since then several sequences have been deposited and nowadays there are 3380 nucleotide accessions available at the NCBI (National Center for Biotechnology Information), whereas 6681 genes are reported in the Ensembl Fungi database. However, since control of PM infection in the field is based on the use of chemical fungicides and canopy management, a deep focus on ‘-omics’ approaches is fundamental to get a broad picture of the pathogen profile. Given the wealth of know-how and technologies becoming available, in the last decade this resulted timely and feasible and the genome of five *E. necator* isolates has been released in 2014 (Jones et al., 2014).

Causal agents of PMs (Ascomycota) are the most important biotrophic fungi together with rusts (Basidiomycota). In the last decade the genome of the most impacting pathogens belonging to Erysiphales were deciphered (Spanu et al., 2010; Wicker et al., 2013; Hacquard, 2014; Jones et al., 2014). Surprisingly the genome-size of these biotrophic pathogens (≈125Mb in *E. necator*) is 3-4 times larger than other Ascomycetes. Sanger sequencing of *Blumeria graminis* (Spanu et al., 2010) and shotgun approaches on *E. necator* (Jones et al., 2014) uncovered that genome expansion is a consequence of transposable element (TEs) and microsatellite accumulation, constituting more than the 60% of the total genome. Moreover, genes encoding enzymes involved in repeat-induced point mutations (RIPs), a natural mechanism to prevent TE accumulation, were lost. Genes, whose products participate in the synthesis of primary and secondary metabolites not required for biotrophy, have been lost too (Hacquard, 2014). This suggests that the high capacity of fungal genome to adapt to different environmental conditions is closely linked to their biotrophic life cycle (Bindschedler et al., 2016). Following the sequencing of five wild strains of *E. necator*, the data comparison confirmed the large number of repetitive elements and the difference in their copy number variations (CNVs) among isolates (Jones et al., 2014). This study demonstrated the adaptive role of CNVs in the establishment of resistance to DMI (sterol demethylase inhibitors) fungicides since structural variations are related to their target protein EnCYP51 (cytochrome P450 lanosterol C-14α-demethylase).

#### Guignardia bidwellii

The identification of *G. bidwellii* commonly relied on morphological analyses and on the observation of the symptoms on the affected plants (Kong, 2009), however these parameters are not sufficient for specific pathogen identification. Currently, a few hundred *G. bidwellii* reference sequences are available in public databases, most of them related to the ITS1 and ITS2 of ribosomal RNA genes (*18S* rRNA and *28S* rRNA), calmodulin and beta-tubulin genes. Some of these sequences (ITS1-2 and calmodulin genes) were used to confirm that Boston ivy infections were due to *G. bidwellii* (Kwon et al., 2015) and to analyze *G. bidwellii* samples collected from several grapevine cultivars and ornamental plants suggesting a specificity at the host genus level (Wicht et al., 2012) (Figure S1A). Detailed phylogenetic trees were generated based on ITS, actin, TEF1A and GAPDH sequences (Zhang et al., 2013). The genetic relationship of *G. bidwellii* (asexual morph *P. ampelicida*) to other *Phyllosticta* species suggested to consider the *parthenocissi* form as a new species.

Currently, full genome sequencing of *G. bidwellii* is not yet available. This would greatly advance the wealth of biological information of this pathogen and it will certainly enhance the potential development of tools aimed to control infections.

#### Elsinoë ampelina

The recognition of AN mostly relies on the observation of symptoms on the plants and/or of fungal morphology by *in vitro* culture, as well as characterization of conidia (Mortensen, 1981; Fan et al., 2017). Recent studies have employed molecular tools to identify isolates using sequences of fungal barcode regions (Seifert, 2009; Schoch et al., 2012), which aim at the characterization and differentiation within the fungal species (Guginski-Piva et al., 2018; Santos et al., 2018a). Sequencing analysis of a selection of genes from *E. ampelina* is also quite recent; the sequencing of ITS, TEF1A and HIS3 in 39 *E. ampelina* isolates collected in 38 vineyards in southern and south-eastern Brazil revealed low genetic variability (Santos et al., 2018b). HIS3 sequence resulted to be the most informative, enabling the grouping of isolates into five haplotypes. Using the same genes, Santos et al. (2018a) compared the sequences of 18 isolates from Brazil and 17 isolates from Australia, where low levels of genetic variability were also detected. Remarkably, ITS and TEF sequences obtained from 35 isolates were identical. Polymorphism was observed only for HIS3 sequences, showing four distinct haplotypes. One of them was most predominant (82.9%) and was observed in both countries, other two were found exclusively in Brazil and one uniquely in Australia. The authors also found cultivation and conidial variation among the isolates, however no relationship was observed between the haplotype network structure and morphological characteristics of the isolates. Different levels of pathogenicity were observed, but this was not related to the origin of the isolates. Currently, there are no reports of complete sequencing, or scaffolding of the genome of *E. ampelina* in public databases. On NCBI 913 nucleotide sequences are available for this fungus, however 676 ESTs of them are related to the expression of host genes when this is challenged by the pathogen. Thus, only 236 deposited sequences are fragments of the genome of *E. ampelina*. Among these, ITS1 and ITS2 are the most representative with 78 and 79 deposited sequences, respectively, as well as the sequence for the 18S rRNA small subunit (SSU rRNA) with 36 sequences deposited and 42 sequences deposited as ssRNA, while for 5.8S and 28S, there are 78 and 36 deposited sequences respectively (Figure S1B).

Sequencing of the barcode regions was also used to identify *C. nymphaeae* as the causal agent of AN in China (Liu et al., 2016) and *C. godetiae* as the causal agent of AN in Italy (Zapparata et al., 2017) and the United Kingdom (Baroncelli et al., 2014). Among the various *Colletotrichum* spp. reported as causing AN, only *C. nymphaeae* and *C. fructicola* have scaffold sequences deposited on NCBI. With regards to *C. nymphaea* (access: JEMN01000491.1) 14,404 genes have been found within a 49.96 Mb genomic region, with 52.7% GC content. While for *C. fructicola* three sequencing projects are reported, one in the contig assembly phase and two with the scaffold of the genome; the bio design with greater detail of the genome (access: ANPB00000000.1) has a sequencing coverage of 55.61 Mb, with 53.5% GC content, where 15,469 genes and 88 pseudogenes were annotated, totaling 15,381 deduced proteins.

## Grapevine-Ascomycete Interaction

### Resistance Loci Identification

#### Powdery Mildew

Conidia of *E. necator* germinate on green tissues of living grapevine plants producing a infection vesicle that penetrates the surface and allows the mycelium to grow between the cells. To cope with the many microorganisms and potential pathogens on their surface, plants have evolved a system of basal immunity. This non-host resistance is based on the recognition of a pathogen-associated molecular pattern (PAMP), probably including chitin in PM (Qiu et al., 2015), sensed by an extracellular receptor-like kinase (RLK) (Dry et al., 2010). The response consists of the production of extracellular antimicrobial compounds and accumulation of molecules to reinforce cell wall as callose papillae. In grapevine-*E. necator* interaction, PAMP-triggered immunity (PTI) can be overcome by the secretion of fungal effectors, detected by the plant and able to trigger effector-triggered immunity (ETI) mediated by resistance (*R*) genes to reach programmed cell death (PCD) (Gadoury et al., 2012).

With regards to PM resistance, in the last decades, it emerged that American and Asian *Vitis* represent a valuable source of *R* genes, which are localized within *R*-loci or genomic intervals (**Table 1**). *Run1* (Resistance to *Uncinula necator* 1) is a single dominant locus on chromosome 12 known to confer high resistance to *E. necator* detected in *M. rotundifolia* (Bouquet, 1986; Barker et al., 2005). Introgressed into a *V. vinifera* background through marker-assisted selection (MAS) (Pauquet et al., 2001), it was found to co-segregate with the *Rpv1* (Resistance to *Plasmopara viticola* 1) locus and to encode full-length and truncated TIR-NBS-LRR (Toll/interleukin-1 receptor-nucleotide-binding site-leucine-rich repeat) resistance proteins (Feechan et al., 2013). Surveys on resistant cultivars showed that this locus is involved in the induction of PCD within penetrated cells at 24 and 48 hours post-inoculation (hpi) (Dry et al., 2010). Subsequently, the *Run2.1* and *Run2.2* loci variants (haplotypes) were identified on chromosome 18 in *M. rotundifolia* ‘Magnolia’ (Riaz et al., 2011), while *Ren5* (misnamed, actually *Run3*) was mapped on chromosome 14 in *M. rotundifolia* ‘Regale’ (Blanc et al., 2012). Resistance to *E. necator* due to PCD was also observed in ‘Kishmish vatkana’ and ‘Dzhandzhal kara’. These related cultivars share the *Ren1* (Resistance to *E. necator* 1) locus carried on the chromosome 13 (Hoffmann et al., 2008; Coleman et al., 2009) and are an exception among the PM resistance donors since they belong to the *V. vinifera proles orientalis*. Very recently, a genome-wide characterization revealed role of NBS-LRR genes during PM infection in *V. vinifera* (Goyal et al., 2019).

**Table 1:** *R*-loci associated with powdery mildew (*Run/Ren*) and black rot (*Rgb*) resistance [improved based on VIVC web source developed by Maul et al. (2012), Merdinoglu et al. (2018), and Hausmann et al. (2019)].

Locus	Chr	Origin of resistence	Genotype of origin	Resistence level	Associated marker	Reference
***Ren1***	13	*V. vinifera*	Kishmish vatkana	Partial	UDV-020	Hoffmann et al., 2008
					VMC9h4-2	Hoffmann et al., 2008
					VMCNg4e10.1	Hoffmann et al., 2008
***Ren2***	14	*V. cinerea*	Illinois 547-1	Partial	CS25	Dalbó et al., 2001
***Ren3***	15	American *Vitis*	Regent	Partial	UDV-015b	Welter et al., 2007
					VVIv67	Welter et al., 2007
					ScORA7-760	Akkurt et al., 2007
					VChr15CenGen02	van Heerden et al., 2014
					GF15-28/VVIv67	Zyprian et al., 2016
					ScORGF15-02	Zendler et al., 2017
					GF15-42	Zendler et al., 2017
***Ren4***	18	*V. romanetii*	C166-043	Partial	VMC7f2	Riaz et al., 2011
			C87-41		SNPs	Mahanil et al., 2012
***Ren5***	14	*M. rotundifolia*	Regale	Total	VMC9c1	Blanc et al., 2012
***Ren6***	9	*V. piasezkii*	DVIT2027	Total	PN9-057	Pap et al., 2016
					PN9-068	Pap et al., 2016
***Ren7***	19	*V. piasezkii*	DVIT2027	Partial	VVIp17.1	Pap et al., 2016
					VMC9a2.1	Pap et al., 2016
					minor QTL of Ren6	
***Ren8***	18	American *Vitis*	Regent	Partial	minor QTL of Ren3	Zyprian et al., 2016
***Ren9***	15	American *Vitis*	Regent	Partial	CenGen6	Zendler et al., 2017
***Ren10***	2	American *Vitis*	Seyval blanc		S2_17854965	Teh et al., 2017
					Haploblock validation	Teh et al., 2017
***Run1***	12	*M. rotundifolia*	VRH3082-1-42	Total	VMC4f3.1	Barker et al., 2005
					VMC8g9	Barker et al., 2005
					49MRP1.P2	Feechan et al., 2013
					CB53.54	Feechan et al., 2013
***Run2.1***	18	*M. rotundifolia*	Magnolia	Partial	VMC7f2	Riaz et al., 2011
					VMCNg1e3	Riaz et al., 2011
					VVIn16	Riaz et al., 2011
***Run2.2***	18	*M. rotundifolia*	Trayshed	Partial	VMC7f2	Riaz et al., 2011
***Rgb1***	14	*V. cinerea*	Boerner	Partial	Gf14-42	Rex et al., 2014
***Rgb2***	16	*V. cinerea*	Boerner	Partial	VChr16c	Rex et al., 2014

In the same years, several Quantitative Trait Loci (QTL) analyses were carried out with the aim to identify new PM resistance loci. Partial resistance is conferred by major QTLs found on different chromosomes. *Ren2* on chromosome 14 confers race-specific resistance in *V. cinerea* (Dalbó et al., 2001; Cadle-Davidson et al., 2016). *Ren*3 on chromosome 15–derived from an undetermined American *Vitis* species–was localized in the variety Regent (Welter et al., 2007) and recently found to determine race-specific hypersensitive response by two different regions on that chromosome; in fact, Zendler et al. (2017) defined the *Ren3* limit and identified *ex novo* the distal *Ren9* locus. In addition, *Ren8* was mapped on chromosome 18 although with an uncertain origin (Zyprian et al., 2016). Besides the American sources, the wild Chinese species *V. romanetii* is donor of a non-race-specific and tissue-independent resistance conferred by the dominant locus *Ren4* on chromosome 18 (Riaz et al., 2011; Mahanil et al., 2012); this was introgressed into *V. vinifera* background to obtain vines able to prevent hyphal emergence from the PM agent (Ramming et al., 2011). Moreover, two major *R*-loci against *E. necator* were discovered in another Chinese species, *V. piasezkii*, *Ren6* and *Ren7* which are respectively localized on chromosome 9 and 19, and they both act in the post-infection stage bringing to PCD. The highest strength of (total) resistance is conferred by *Ren6*, even higher than *Run1*, while *Ren7* is responsible of a weak partial resistance to the pathogen (Pap et al., 2016). Finally, Teh et al. (2017) identified the new *Ren10* locus on chromosome 2 acting moderately against PM sporulation (**Table 1**).

Given the reported advances also on the molecular characterization of the studied Ascomycetes, we can assert that nowadays pathogen genetics can inform host genetics and host-pathogen interaction mechanisms. For instance, in the Eastern US, where the pathogen co-evolved with many mapped PM resistance genes, the *Ren2* locus has recently fully broken down and is no longer detectable in the vineyard (Cadle-Davidson, 2018). Actually, in North America naturally occurring isolates displaying virulence on vines carrying the *Run* loci were already observed demonstrating that qualitative (vertical) resistance is strong, but since it is race-specific can be easily overcome (Feechan et al., 2015). By contrast, partial (horizontal) resistance–which typically is controlled by at least 4-5 QTLs–is usually more durable, particularly when it involves morphological or developmental changes in the plant, although might be prone to gradual loss (erosion) in the long term (Stuthman et al., 2007). Therefore, to achieve long lasting resistance, the combination of both types is needed; this process, named *R* gene pyramiding, relies on genetics built into vines.

Pyramiding entails stacking multiple genes leading to the simultaneous expression of more than one gene in a cultivar to develop durable resistance expression. Gene pyramiding is gaining considerable importance as it would improve the efficiency of plant (including grapevine) breeding, leading to the development of genetic stocks and precise development of broad spectrum resistance capabilities (Joshi and Nayak, 2010). Both wine and table grape breeding programs for PM resistance around the world are using this approach, providing endurance both in term of absolute disease resistance degree (e.g. Feechan et al., 2015) and of the degree fluctuation reduction during different years (e.g. Zini et al., 2019). According to the most updated information on MAS applications at European level, in France “ResDur” varieties presenting assorted combinations of *Rpv1, Rpv3, Rpv10* associated with DM and of *Run1, Ren3* and *Ren3.2* associated with PM resistance were obtained by breeding “Bouquet” varieties with American, Asian and wild *Vitis* backgrounds (Delmotte et al., 2018). In Italy, Vezzulli et al. (2019) were able to obtain pyramided genotypes carrying two or three *Run/Ren* loci, up to seven *R*-loci in total, while Foria et al. (2019) developed resistant genotypes derived from “elite” cultivars carrying *Rpv1, Rpv12* coupled with *Run1* and *Ren3*. Finally, besides increasing host diversity and complexity, to achieve higher durability, populations of biotrophic pathogens should be regularly monitored for their virulence frequencies and virulence combinations (Miedaner, 2016).

In order to indirectly dissect PM resistance, an alternative approach relies on the biological candidacy of susceptibility (*S*) genes. Unlike *R* genes, *S* genes are required for successful pathogen infection, and thus are considered essential for compatible plant-pathogen interactions. A state of the art about the investigation and the future application of grapevine *S* genes associated with PM susceptibility is reported in the Text S1.

#### Black Rot

In contrast to PM, for which several *R*-loci have already been discovered in various grapevine cultivars, very little is known about the genetic loci involved in BR resistance (**Table 1**). It has been observed almost a century ago that North American and French hybrids are more resistant to *G. bidwellii* infection, while *V. vinifera* is extremely susceptible (Demaree et al., 1937; Barrett, 1955; Hausmann et al., 2017). The American origin of this ascomycete explains the occurrence of genetic resistance in American *Vitis* species, due to their coevolution (Ramsdell and Milholland, 1988; Hausmann et al., 2017). Hints about the genetic segregation pattern of BR resistance were suggested in a ‘Blue Lake’ progeny by Mortensen (1977), but the first QTL analysis was performed much later by Dalbó et al. (2000). They studied the inheritance of BR resistance in the cross ‘Horizon’ (‘Seyval’ × ‘Schuyler’) × ‘Illinois 547-1’ (*V. rupestris* × *V. cinerea*) and constructed a genetic map based on RAPD markers for each parent. This analysis identified one QTL on chromosome 14 associated with the CS25b marker (Dalbó et al., 2000; Dalbó et al., 2001). Interestingly, the latter map interval was also associated with PM resistance though to different degrees (Dalbó et al., 2000).

Later Rex et al. (2014) carried out a QTL analysis using a cross between a cultivar that shows high resistance to BR (cultivar ‘Börner’, an inter-specific hybrid of *V. riparia* × *V. cinerea*) and a susceptible breeding line (V3125, ‘Schiava grossa’ × ‘Riesling’). They performed six independent resistance tests in a climate chamber and one disease evaluation in the field, and identified two *R*-loci; the major QTL on chromosome 14, called *Resistance to G. bidwellii1* (*Rgb1*) co-mapped with the QTL identified by Dalbó et al. (2000). A second QTL on chromosome 16 (named *Rgb2*) was detected both in climate chamber and in field analysis, while the results on other minor QTLs were not reproducible. All these data suggest a polygenic nature of the resistance (Rex et al., 2014). The analysis of Sequence-Tagged Sites (STS) markers did not allow a more accurate delineation of the *Rgb2* locus position. For *Rgb1* locus, the fine mapping on chromosome 14 restricted the region of QTL to about 2.4 Mb in the reference grapevine genome and allowed the authors the development of a new marker, GF14-42, that showed a strong association with *Rgb1*. Some genes with a putative defense function were annotated in the genomic region of *Rgb1* (i.e. chitinase, RIN4-like protein, MAP kinase and F-box domain protein; Rex et al., 2014). The QTL site on chromosome 14 was also detected in another biparental mapping population derived from a cross between ‘GF.GA-47-42’ × ‘Villard Blanc’ with both parents being resistant to BR. This study revealed the presence of seven *R*-loci distributed over the genome. The major QTL on chromosome 14 in this mapping population was tightly linked to the SSR markers GF14-04 and UDV-095, both are located in the vicinity of the above mentioned markers CS25b and GF14-42 (Hausmann et al., 2017) (**Table 1**). All these data suggest that the major *R*-locus on chromosome 14, identified in three different biparental population, plays a leading role in conferring BR resistance. Finally, from the application point of view, nowadays some new bred varieties with a good field resistance to BR – carrying *Rgb* loci – are available (e.g. Töpfer and Eibach, 2016; Töpfer et al., 2018).

#### Anthracnose

In contrast to *E. necator* and *G. bidwellii* no QTL analysis has been published for *E. ampelina*. This is perhaps due to the fact that AN is not a major disease in the main grapevine production regions, such as Europe. However, the use of resistant cultivars, which reduces the amount of fungicide associated with the climatic break-down expected, might increase the incidence of this disease also in these regions. Therefore, the development of new varieties resistant to AN will be a major task worldwide in the near future and it is essential to develop molecular markers linked to AN resistance genes.

Resistance sources to *E. ampelina* have been identified through natural infection in field screenings (Fennell, 1948; Mortensen, 1981; Wang et al., 1998; Yun et al., 2006; Li et al., 2008; Louime et al., 2011; Poolsawat et al., 2012), by artificial infection in greenhouse (Hopkins and Harris, 2000), by detached-leaf assays (Kono et al., 2012; Poolsawat et al., 2012) and filtrate culture (Yun et al., 2006; Kim et al., 2008; Jang et al., 2011; Louime et al., 2011). In the American genepool, variable levels of resistance to AN were found in different *Vitis* spp. Based on natural infections, Fennell (1948) reported that the majority of the American tropical grapevine species show moderate to good resistance to AN. Accessions of *V. gigas* (Fen), *V. rufotomentosa* (Small) and *V. smalliana* (Bailey) were classified as highly resistant. Similarly, Mortensen (1981) reported as sources of resistance to AN accessions of *M. rotundifolia* Michx., *V. aestivalis* ssp. *simpsonii*, *V. aestivalis* ssp. *smalliana, V. caribeae* DC (syn. *V. tiliafolia* Humb. & Bonpl.)*, V. champini* Planch., *V. labrusca* L., *V. munsoniana* Simps, *V. rupestris* Scheele, *V. shuttleworthii* House and *V. vulpina* L. Field evaluations under natural infection of *E. ampelina* revealed that all the Asian *Vitis* species tested were classified as highly resistant or resistant to AN (Wang et al., 1998; Li et al., 2008). The species evaluated were *V. adstricta*, *V. amurensis*, *V. bashanica*, *V. betulifolia*, *V. bryoniifolia*, *V. davidii*, *V. flexuosa*, *V. hancockii*, *V. liubanensis*, *V. piazezkii*, *V. pseudoreticulata*, *V. qinlingensis*, *V. quinquangularis*, *V. romanetii*, *V. sinocinerea*, *V. wilsonae* and *V. yenshanensis*. In the same fields, the *V. vinifera* cvs. Cabernet Sauvignon, Carignane and Chardonnay were classified as susceptible, highly susceptible and highly susceptible, respectively.

Under natural conditions the *V. vinifera* cultivars evaluated (e.g. Alexandria, Cabernet Sauvignon, Cardinal, Carignane, Chardonnay, Chasselas Golden, Exotic, Lignan Blanc, Malaga, Muscat of Alexandria, Perlette, Red Muscat, Sultanina, Thompson Seedless) were classified as susceptible or highly susceptible to the disease (Fennell, 1948; Mortensen, 1981; Wang et al., 1998; Li et al., 2008). In contrast, a detached leaf assay with Exotic and Perlette cultivars possess moderate resistance (Kono et al., 2012). Fennell (1948) reported that, when tropical species were crossed to the highly susceptible *V. vinifera* cultivars, all the F_1_ progenies were susceptible. The susceptibility was conditioned by two or three dominant genes, and the resistance transmitted as a recessive locus. A more detailed investigation to elucidate the inheritance of AN resistance of American *Vitis* species was performed by Mortensen (1981). His genetic strategy pinpointed that AN susceptibility/resistance is controlled by three genes, two dominants for susceptibility (*An1* and *An2*), and one dominant (*An3*) conferring resistance. The three genes segregate independently. When both dominant susceptibility alleles (*An1* and *An2*) are present, the phenotype is susceptible regardless of whether *An3* is present or no. If either *An1* or *An2* or both are not present, than *An3* conditions resistance and *an3* susceptibility. According to Mortensen (1981), most *V. vinifera* cultivars are homozygous dominant for both *An1* and *An2* (e.g. Cabernet Sauvignon; *An1An1*/*An2An2*/*an3an3*), complicating the introgression of AN resistance genes from American species into *V. vinifera* background. On the other hand, F_1_ progenies derived from crosses between hybrids with variable levels of genetic composition from several American species (*V. cinerea*, *V. riparia*, *V. rupestris*, *V. labrusca*, and *V. lincecumii*, along with *V. vinifera*) and *V. vinifera* segregated for the resistance to AN (Kim et al., 2008; Poolsawat et al., 2013). The proportion of resistant and susceptible plants in the progenies obtained by Kim et al. (2008) suggests that the resistance is conferred by a single dominant gene. One example is the crossing between Concord (resistant) and Neomuscat (susceptible), for which a proportion of 1 resistant: 1 susceptible was obtained. The disease phenotyping was done by culture filtrates. In accordance, Poolsawat et al. (2013) reported high narrow sense heritability for AN resistance, suggesting the prevalence of additive over non-additive gene action. On the other hand, the crossing of a susceptible hybrid with a susceptible *V. vinifera* cultivar also segregated for AN resistance, suggesting the presence of susceptibility genes (Poolsawat et al., 2013).

The resistance present in the Asian species seems to be monogenic and dominantly inherited, without the presence of susceptibility genes. Wang et al. (1998) reported that all the progenies derived from the crosses between the Asian *Vitis* species *V. amurensis*, *V. davidii*, *V. piasezkii*, *V. pseudoreticulata*, *V. quinquangularis* and *V. romanetii* with *V. vinifera* were resistant to the disease, like their native parents used as resistant donor. The absence of susceptibility genes renders the Asian germplasm very interesting for breeding, since the segregation of resistance in the first generation saves time in the process of gene introgression. Finally, it is important to mention that in all progenies evaluated, a continuum variation of the AN symptoms were observed, suggesting also a quantitative inheritance of the disease resistance (e.g. Fennell, 1948; Mortensen, 1981; Yun et al., 2006). Taking all the data together, it may indicate that major genes–which explain the main variation observed–together with minor genes–confer the resistance to AN, as observed in other pathosystems. So far, only one work describes the genetic mapping of genes conferring resistance to AN (Kim et al., 2008). A RAPD marker closely linked to an AN-resistant gene was identified using bulked-segregant analysis (BSA) and was converted into a SCAR marker. The SCAR amplifies a specific band in resistant cultivars/hybrids with different American backgrounds (*V. berlandierii, V. champinii*, *V. labrusca, V. riparia, V. rupestris*) and no band in susceptible ones, mainly *V. vinifera* cultivars. However, no information was found about the use of the SCAR in routine breeding programs to assist the introgression of the resistant gene into *V. vinifera* germplasm. Recently, also RGA-SSCP markers have been found associated with AN resistance (Tantasawat et al., 2012).

Finally, AN resistance was also pursuit in an *in vitro* study carried out to select resistant cv. Chardonnay (*V. vinifera*) plants using *E. ampelina* culture filtrates (Jayasankar et al., 2000).

### Transcript, Protein and Metabolite Exploration

#### Erysiphe necator

To date, several studies aimed to decipher the interaction between different *Vitis* genetic backgrounds and *E. necator* at different molecular levels, i.e. by analyzing transcripts, proteins and metabolites.

As an obligate biotroph, one of the most challenging steps for transcriptional characterization of *E. necator* is the harvest of fungal tissue free of contaminating grapevine material. The technique for the isolation of fungal RNA developed by Cadle-Davidson et al. (2010) constituted a turning point. Taking advantage of it, Wakefield et al. (2011) investigated the genetic regulation of conidiation in *E. necator*. They identified new genes involved in conidiation, never found before in other filamentous fungi, probably related to the obligate biotrophic lifestyle. With the advent of next generation sequencing technologies additional progresses have been made. *De novo* transcriptomes were used to detect polymorphisms between different isolates of *E. necator* (Myers, 2012) as well as to identify candidate effector proteins specific to haustoria (Barnett, 2015). Transcriptome analyses, in addition to assisting in genome annotation (Jones et al., 2014), revealed the presence of several transcripts derived from transposable elements, indicating that they are transcriptionally active and can account for the observed genome expansion (Jones et al., 2014; Snyder, 2018).

Similarly, a large number of studies have been carried out to determine the genetic basis of resistance in *Vitis* species (**Tables 2A** and S2A). Plants react to pathogen attack with multiple common defense responses, such as cell wall reinforcements through callose and lignin synthesis at the site of attempted penetration and accumulation of antimicrobial secondary metabolites (phytoalexins) and of pathogenesis-related (PR) proteins (e.g. chitinases and glucanases, able to degrade fungal and oomycete cell walls). Pathogen recognition can also be followed by a burst of reactive oxygen species (ROS) culminating in a PCD at the site of invasion (Wilkinson et al., 2019). Moreover, plant defense responses are tightly regulated by hormone-mediated signaling pathways, with jasmonate (JA) and salicylic acid (SA) playing the main role (Berens et al., 2017). Genes implicated in PM resistance can be identified thanks to their activation or increased transcription during infection and/or different expression levels between resistant and susceptible species. First significant data on grapevine resistance mechanisms were showed by Jacobs et al. (1999), which observed the specific and local induction of some PR genes in susceptible *V. vinifera* cultivars in response to PM infection. Godfrey et al. (2007) characterized the *V. vinifera Germin-like proteins* (*GLP*) gene family, ubiquitous plant proteins known to be involved in the response to various stress conditions including plant defense against pathogens (Bernier and Berna, 2001), and found that *VvGLP3* was specifically and locally induced in the epidermal cells in response to pathogen attack, suggesting a role in the penetration-based defense response against PM infection.

**Table 2:** Transcriptomics, proteomics and metabolomics of grapevine-ascomycete interaction: *E. necator* (panel A) and *E. ampelina* (panel B).

A)													
Gene or Metabolite		Description		Taxon		Genotype		Defense Response		Organ		Reference	
PR proteins, e.g. chitinases (PR-2), glucanases (PR-3) and thaumatin-like (TL) proteins (PR-5)		Pathogenesis-Related protein		*V. vinifera* spp. *sativa*		Sultana, Cabernet Sauvignon		Susceptible		Leaves, berries		Jacobs et al., 1999	
*VvGLP3*		Germin-like proteins 3		*V. vinifera* spp. *sativa*		Chardonnay		Susceptible		Leaves, berries		Godfrey et al., 2007	
*VvGLP4*		Germin-like proteins 4											
*Several gene families*				*V. vinifera* spp. *sativa*		Cabernet Sauvignon		Susceptible		Leaves		Fung et al., 2007	
				*V. aestivalis*		Norton		Resistant					
*Several gene families*				*V. vinifera* spp. *sativa*		Cabernet Sauvignon		Susceptible		Leaves		Fung et al., 2008	
				*V. aestivalis*		Norton		Resistant					
*Several gene families*				*V. vinifera* spp. *sativa*		Cabernet Sauvignon		Susceptible		Leaves		Fekete et al., 2009	
*Several gene families*				*V. pseudoreticulata*		Baihe-35-1		Resistant		Leaves		Xu et al., 2009	
*VaEDS1, VvEDS1*		Orthologs of *Arabidopsis thaliana ENHANCED DISEASE SUSCEPTIBILITY1*		*V. aestivalis* *V. vinifera* spp. *sativa*		Norton Cabernet Sauvignon		Resistant Susceptible				Gao et al., 2010	
*VpWRKY1, VpWRKY2*		WRKY domain transcription factor		*V. pseudoreticulata*		Baihe-35-1, Baihe-13, Baihe-13-1, 6-12-6, Guangxi-1		Resistant		Leaves		Li et al., 2010	
				*V. pseudoreticulata*		Guangxi-2, Hunan-1, 6-12-2, Shangnan-2, Baihe-35-2		Susceptible					
				*V. vinifera* spp. *sativa*		Carignane		Susceptible					
Several gene families				*V. vinifera* spp. *sativa*		Cabernet Sauvignon		Susceptible		Leaves		Marsh et al., 2010	
*VpSTS*		Stilbene synthase		*V. pseudoreticulata*		Baihe-35-1		Resistant				Xu et al., 2010a	
*VpPR10*		Pathogenesis-Related protein 10		*V. pseudoreticulata*		Baihe-35-1		Resistant		Leaves		Xu et al., 2010b	
*VpGLOX*		Glyoxal oxidase		*V. pseudoreticulata*		Baihe-35-1		Resistant				Guan et al., 2011	
				*V. pseudoreticulata*		Guangxi-2		Susceptible					
*VpRFP1, VvRFP1*		C4C4-type RING finger protein		*V. pseudoreticulata*		Baihe-35-1		Resistant		Leaves		Yu et al., 2011	
				*V. vinifera* spp. *sativa*		Carignane		Susceptible					
*STS*		Stilbene synthase		*V. pseudoreticulata*		Baihe-35-1		Resistant		Leaves		Xu et al., 2011b	
				*V. vinifera* spp. *sativa*		Thompson Seedless		Susceptible					
				*V. vinifera* spp. *sativa*		Carignane		Susceptible					
*STS8, STS13, STS16, STS17, STS22, STS23, STS27, STS31*		Stilbene synthase		*V. aestivalis*		Norton		Resistant		Leaves, berries		Dai et al., 2012	
				*V. vinifera* spp. *sativa*		Cabernet Sauvignon		Susceptible					
				*V. quinquangularis*		Shang-24		Resistant		Leaves		Gao et al., 2012a	
Several gene families				*V. pseudoreticulata*		Hunan-1		Susceptible					
*VpALDH2B4*		Aldehyde dehydrogenase		*V. pseudoreticulata*		Baihe-35-1		Resistant				Wen et al., 2012(Wang et al., 2007)	
*VpWRKY3*		WRKY domain transcription factor		*V. pseudoreticulata*		Baihe-35-1		Resistant		Leaves		Zhu et al., 2012a	
*VpNAC1*		NAC transcription factor		*V. pseudoreticulata*		Baihe-35-1		Resistant		Leaves		Zhu et al., 2012b	
Several gene families				*V. vinifera* spp. *sativa*		Touriga Nacional		Susceptible		Leaves		Borges et al., 2013	
*VpRFP1, VvRFP1*		C4C4-type RING finger protein		*V. pseudoreticulata*		Baihe-35-1		Resistant				Yu et al., 2013a	
				*V. vinifera* spp. *sativa*		Carignane		Susceptible					
*VpEIRP1*		E3 ubiquitin ligase *E. necator*-induced C3HC4-type Really Interesting New Gene (RING) finger protein 1		*V. pseudoreticulata*		Baihe-35-1		Resistant		Leaves		Yu et al., 2013b	
*VpWRKY11*		WRKY domain transcription factor		*V. pseudoreticulata*		Baihe-35-1		Resistant					
*VpERF* genes		Ethylene response factor		*V. pseudoreticulata*		Baihe-35-1		Resistant				Zhu et al., 2013	
*VvWRKY* family		WRKY domain transcription factor		*V. aestivalis*		Norton		Resistant		Leaves		Wang et al., 2014	
				*V. vinifera* spp. *sativa*		Cabernet Sauvignon		Susceptible					
				*V. pseudoreticulata*		Baihe-35-1		Resistant					
*VvNPF3.2*		NITRATE TRANSPORTER1/PEPTIDE TRANSPORTER FAMILY		*V. vinifera* spp. *sativa*		Cabernet Sauvignon		Susceptible				Pike et al., 2014	
				*V. aestivalis*		Norton		Resistant					
*trans*-Resveratrol				*V. quinquangularis* Rehd.		Danfeng-2, Taishan-12, 83-4-96, Shangnan-24				Berries		Shi et al., 2014	
				*V. ficifolia Bunge*		Weinan-3							
				*V. amurensis* Rupr.		Shuangyou, Zuoshan-1, Zuoshan-2							
				*V. piasezkii* Maxim.		Liuba-7							
				*V. pseudoreticulata* W.T.Wang		Hunan-1, Guangxi-1							
				*V. thunbergii* Sieb.et Zucc		Anlin-3							
				*V. yeshanensis* J.X.Chen		Yanshan-1							
				*V. vinifera* spp. *sativa*		Ugni Blanc, Pinot Noir, Carignane, Cabernet Sauvignon							
*STS* family; *trans*-Resveratrol				*V. quinquangularis*		Danfeng-2		Resistant		Leaves, berries			
				*V. vinifera* spp. *sativa*		Pinot Noir		Susceptible					
*VpPR10-1*		Pathogenesis-Related protein 10		*V. pseudoreticulata*		Baihe-35-1		Resistant				Xu et al., 2014	
*REN1*-associated genes				*V. vinifera* spp. *sativa*		Carignane		Susceptible		Leaves		Amrine et al., 2015	
				*V. vinifera* spp. *sativa*		Late Vavilov		Mid-Susceptible					
				*V. vinifera* spp. *sativa*		Husseine, Khalchili, Sochal		Mid-Resistant					
				*V. vinifera* spp. *sativa*		Karadzhandal		Resistant					
				*V. vinifera* spp. *sylvestris*		O34-16, DVIT3351.27		Resistant					
*Several gene families*				*V. pseudoreticulata*		Baihe-13-1		Resistant		Leaves		Jiao et al., 2015	
				*V. pseudoreticulata*		Hunan-1		Susceptible					
				*V. quinquangularis*		Shang-24		Resistant					
*VpCN*		Disease resistance protein RxCC-like-NB-ARC		*V. pseudoreticulata*		Baihe-35-1		Resistant		Leaves		Wen et al., 2015	
*VqSTS6*		Stilbene synthase		*V. quinquangularis*		Danfeng-2		Resistant				Cheng et al., 2016	
*VpPR4-1*		Pathogenesis-Related protein 4		*V. pseudoreticulata*		Baihe-35-1		Resistant		Leaves		Dai et al., 2016	
vvi-NewmiR2118 (*MIR2118*)		miRNA		*V. pseudoreticulata*		Baihe-35-1		Resistant		Leaves		Han et al., 2016	
*VfMlo-like* gene family		Powdery-mildew resistance locus o-like		*V. flexuosa*		VISKO001		Resistant		Leaves		Islam and Yun 2016a	
*VpSTS, VvSTS*		Stilbene synthase		*V. pseudoreticulata*		Baihe-35-1		Resistant				Jiao et al., 2016	
				*V. vinifera* spp. *sativa*		Carignane		Susceptible					
*Several gene families*				*V. vinifera* spp. *sativa*		Cabernet Sauvignon		Susceptible		Leaves		Toth et al., 2016	
*VqDUF642*		Domain of Unknown Function 642		*V. quinquangularis*		Danfeng-2		Resistant		Leaves, berries		Xie and Wang, 2016	
*VpUR9*		RING-type ubiquitin ligase gene		*Vitis pseudoreticulata*		Baihe-35-1		Resistant		Leaves		Dai et al., 2017	
*VqMAPKKK38*		Raf-like Mitogen-activated protein kinase kinase kinase		*V. quinquangularis*		Danfeng-2		Resistant		Leaves		Jiao et al., 2017	
*VpEIFP1*		F-box/Kelch-repeat protein		*Vitis pseudoreticulata*		Baihe-35-1		Resistant		Leaves		Wang et al., 2017a	
*VpRH2*		RING-H2-type ubiquitin ligase		*V. pseudoreticulata*		Baihe-35-1		Resistant		Leaves		Wang et al., 2017b	
				*V. vinifera* spp. *sativa*		Thompson seedless		Susceptible					
*VqWRKY52*		WRKY domain transcription factor		*V. quinquangularis*		Shang-24		Resistant		Leaves		Wang et al., 2017c	
*VaSTS19*		Stilbene synthase		*V. amurensis* Rupr.		Tonghua-3		Resistant		Leaves		Wang et al., 2017d	
*VpTNL1*		TIR-NB-ARC-LRR R protein		*V. pseudoreticulata*		Baihe-35-1		Resistant		Leaves		Wen et al., 2017	
*VvTLP*gene family		Thaumatin-like protein (TLP)		*V. vinifera*		Red Globe		Susceptible		Leaves		Yan et al., 2017	
*VqTLP29*				*V. quinquangularis*		Shang-24		Resistant					
				*V. pseudoreticulata*		Hunan-1		Susceptible					
*VqJAZ7*		Jasmonate ZIM-domain (JAZ) transcriptional repressor		*V. quinquangularis*		Shang-24		Resistant		Leaves		Hanif et al., 2018	
**B)**													
**Gene or Metabolite**		**Description**		**Taxon**		**Genotype**		**Defense Response**		**Organ**		**Reference**	
*NBS-LRR*family		Nucleotide Binding Sites-Leucine Rich Repeats proteins		*V. vinifera* spp. *sativa*		DVIT3351.27, Husseine, O34–16, Karadzhandal, Khalchili, Late vavilov, Sochal		From mid-susceptible to resistant				Goyal et al., 2019	
						Carignane, Thompson seedless		Susceptible					
*ATP Synthase, GS*		Adenosine Triphosphate Synthase beta subunit, Glutamine Synthetase		*Vitis interspecific crossing* (Florida hybrids)		Lake Emerald, Blue Lake		Tolerant		Leaves		Vasanthaiah et al., 2009	
*Rubisco*		Ribulose 1-5 bisphosphate-carboxylase		*Vitis interspecific crossing* (Florida hybrids)		Blanc du Bois, Suwannee		Susceptible					
				*Muscadinia (or Vitis) rotundifolia* (muscadine grape)		cv. Carlos		Tolerant					
*CHI* *STS*		Chitinase Stilbene Synthase Transcription factor, Protein kinase, Sugar kinase genes		*Vitis interspecific crossing* (Florida hybrids)		Lake Emerald, Blue Lake		Tolerant		Leaves		Vasanthaiah et al., 2010	
				*Vitis interspecific crossing* (Florida hybrids)		Blanc du Bois, Suwannee		Susceptible					
*STS, CHS, CHI, PGIP, LIP*		Stilbene Synthase, Chalcone Synthase (CHS), Chitinase (CHI), Polygalacturonase Inhibiting Protein (PGIP), Lipid Transfer Protein (LIP)		*M. rotundifolia*		Derived selections		from susceptible to tolerant		Leaves		Louime et al., 2011	
Several gene families				*V. coignetiae*				Resistant		Leaves		Choi et al., 2011	
Several gene families				*V. quinquangularis*		Shang-24		Resistant		Leaves		Gao et al., 2012b	
				*V. vinifera*		Red Globe		Susceptible					
Several gene families				*V. flexuosa*		VISKO001		Resistant		Leaves		Ahn et al., 2014a	
*VfGlu* gene family		β-1,3-glucanase		*V. flexuosa*		VISKO001		Resistant		Leaves		Ahn et al., 2014b	
*VfRPS5-like* gene family		Resistance to *Pseudomonas syringae* 5 (*RPS5*), member of the NBS-LRR family		*V. flexuosa*		VISKO001		Resistant		Leaves		Islam et al., 2015a	
*VfRLK* gene family		Receptor-like protein kinase		*V. flexuosa*		VISKO001		Resistant		Leaves		Islam et al., 2015b	
*VfMlo-like* gene family		Powdery-mildew resistance locus o-like		*V. flexuosa*		VISKO001		Resistant		Leaves		Islam and Yun 2016a	
*VfCXE* gene family		Carboxylesterase		*V. flexuosa*		VISKO001		Resistant		Leaves		Islam and Yun 2016b	
*VfGST* gene family		Glutathione S-transferase		*V. flexuosa*		VISKO001		Resistant		Leaves		Ahn et al., 2016	
*VfEDL1, VfEDL2, VfEDL3*		Enhanced Disease Susceptibility 1 (EDS1)-like1		*V. flexuosa*		VISKO001		Resistant		Leaves		Islam and Yun 2017	
*VvTLP*gene family		Thaumatin-like protein (TLP)		*V. vinifera*		Red Globe		Susceptible		Leaves		Yan et al., 2017	
*VqTLP29*				*V. quinquangularis*		Shang-24		Resistant					
				*V. pseudoreticulata*		Hunan-1		Susceptible					

A) Erysiphe necator; B) Elsinoë ampelina.

A more comprehensive view of the networks and mechanisms mediating plant defense was obtained through high-throughput methods, able to monitor transcriptome and proteome-wide changes. Comparative transcriptomic studies revealed substantial variation in gene expression between *V. vinifera* and the resistant *V. aestivalis*, which showed a constitutively elevated expression of several PR genes, as well as higher endogenous SA levels (Fung et al., 2007; Fung et al., 2008). Moreover, the very weak PM-induced response occurring in *V. aestivalis*, unlike the major transcriptome remodelling observed in *V. vinifera*, suggests that the constitutive transcriptomic profile of the resistant genotype is already defense-oriented (Fung et al., 2008). Transcriptome and proteome changes observed in Cabernet Sauvignon during infection (Fekete et al., 2009; Marsh et al., 2010) demonstrated that the susceptible plant is able to initiate a basal defense response, since several defense-related transcripts and proteins, as well as SA, accumulate in infected leaves. This response however turns out to be insufficient to restrict fungal growth, thanks to the ability of the pathogen effectors to suppress the host defense system, as already observed in the barley (*Hordeum vulgare*)-PM agent (*Blumeria graminis* f. sp. *hordei*) interaction (Caldo et al., 2004; Caldo et al., 2006). Indeed, the expression levels of many grapevine defense-related genes reached a maximum at 12 hpi and then declined as the fungal infection became established (Fung et al., 2008).

In addition to differential transcriptional regulation, species- or cultivar-specific genes may contribute substantially to determine variable disease susceptibility among *Vitis* species (Da Silva et al., 2013; Venturini et al., 2013). Xu et al. (2009) identified several ESTs potentially associated with plant defense responses in *V. pseudoreticulata*. Gao et al. (2012a) focused on PM-induced gene expression changes in *V. quinquangularis*. Among the genes differentially expressed, a large part encoded for resistance and stress-related proteins, and some of them showed specific PM-induction only in the resistant genotype.

High-throughput transcriptomic analyses were performed more recently by next-generation sequencing (RNA-seq). Weng et al. (2014) investigated how global gene expression profile changes in response to PM infection in *V. pseudoreticulata*. They identified several genes and pathways that may contribute to PM resistance and found that the enhancement of JA pathway, systemic acquired resistance (SAR) and ROS-dependent hypersensitive responses as well as the accumulation of phytoalexins play a key role in the defense response mechanism. Jiao et al. (2015)*de novo* assembled the transcriptomes of three wild Chinese *Vitis* accessions and studied the differences with the reference genome. A large number of distinct transcripts were identified in the resistant accessions, which resulted to be highly enriched in genes involved in plant secondary metabolisms, such as biosynthesis of phenolic compounds, and defense responses. These studies, together with other research done in grapevine (e.g. Borges et al., 2013) and in several other plant-PM agent interactions (e.g. Xu et al., 2011; Fu et al., 2016; Zhu et al., 2018; Polonio et al., 2019; Tian et al., 2019), further highlight the contribution of secondary metabolites to the plant defense mechanisms. Amrine et al. (2015) studied wild and cultivated Central Asian *V. vinifera* accessions carrying a common *Ren1* locus but displaying significant variations in disease susceptibility. They identified several genes potentially involved in the *Ren1*-dependent resistance mechanisms, as well as genes whose expression levels correlated with the different levels of resistance observed among accessions. These transcriptomic data have recently been used by Goyal et al. (2019) to investigate the role played by the *NBS-LRR* genes. They identified a total of 63 PM-responsive *NBS-LRR* genes in different *V. vinifera* accessions, ranging from susceptible to partially resistant. Gene expression levels in response to PM infection changed greatly between cultivars. Some genes were either up-regulated or expressed only in partially resistant cultivars. Moreover, at 5 dpi most of the genes were up-regulated in all the accessions, indicating a putative role in later stages of infection.

Since 1999, in different genetic backgrounds several genes that may contribute to PM resistance have been identified, such as *VpGLOX* (Guan et al., 2011); *VpPR-10.1* (Xu et al., 2010b; Xu et al., 2014); *VpRFP1* (Yu et al., 2011); *VpALDH2B4* (Wen et al., 2012); *VpNAC1* (Zhu et al., 2012b); *VpPR4-1* (Dai et al., 2016) (**Tables 2A** and S2A). Many of them are transcription factors (Zhu et al., 2012a; Zhu et al., 2012b), as expected since they are master regulators in controlling plant response to biotic stress (Amorim et al., 2017; Ng et al., 2018). In particular, *WRKY* transcription factors play an important role in plant defense (Pandey and Somssich, 2009) and WRKYs conferring resistance towards bacterial or fungal agents have been identified in several plants (Phukan et al., 2016). *WRKY* genes involved in response to fungal pathogens had been identified also in *V. vinifera*, i.e. *VvWRKY1* (Marchive et al., 2007) and *VvWRKY2* (Mzid et al., 2007). The entire *V. vinifera WRKY* gene family was later characterized by Wang et al. (2014). Interestingly, a significant induction of many *VvWRKY* genes occurred in the susceptible cv. Cabernet Sauvignon in response to PM infection, unlike the resistant cultivar Norton which however showed a constitutive higher level of *WRKY* genes expression, potentially confirming their role in defense even in resistant cultivars. Other *WRKY* genes implicated in PM resistance were identified in *V. pseudoreticulata* (Li et al., 2010; Zhu et al., 2012a; Yu et al., 2013b) and *V. quinquangularis* (Wang et al., 2017c).

Stilbenes, the grapevine phytoalexins, play a critical role in plant defense (Schnee et al., 2008). In grapevine stilbene synthase (*STS*) genes, the key enzyme in the biosynthesis of stilbenic compounds, are organized in an unusually large family (Parage et al., 2012; Vannozzi et al., 2012). Expression analyses performed in *V. vinifera* and *V. aestivalis* showed that individual *STS* genes are differentially expressed during leaf and berry development as well as in response to PM attack (Dai et al., 2012). PM-induction of *STS* genes was observed mainly in *V. vinifera*, whereas *V. aestivalis* showed constitutively higher transcript levels of some *STS* genes (Fung et al., 2008; Dai et al., 2012). Chinese wild grapevines are an important source of resistance genes related to stilbene production since several of them have a significantly higher resveratrol content than most European *V. vinifera* cultivars, and this accumulation correlates with *STS* genes expression. Indeed, both the endogenous and the PM-induced *STS* genes expression levels were much higher in the resistant *V. quinquangularis* than in *V. vinifera* (Shi et al., 2014). Comparative analyses of *STS* genomic regions and promoter activities in resistant and susceptible species revealed that the differential responsiveness of the *STS* genes is associated with differences in their promoter sequences, whereas the coding regions are highly conserved (Xu et al., 2010a; Xu et al., 2011b; Jiao et al., 2016). Jiao et al. (2017) identified in *V. quinquangularis VqMAPKKK38*, a PM-inducible Raf-like MAPKKK gene involved in the very complex network controlling activation of *STS* transcription.

Unlike the majority of the studies seen so far, which focus mainly on the early or midterm stages of infection, Borges et al. (2013) investigated the specific long term remodeling of the grapevine transcriptome operated by the pathogen to promote its survival, which requires uptake of nutrients and suppression of host defense responses. Interestingly none of the differentially expressed genes identified had correspondence to the ones detected by Fekete et al. (2009) in the early stages of infection. The mechanisms used by the pathogen to modulate host physiology were further investigated by Pike et al. (2014) through the functional characterization of *VvNPF3.2*, a putative pathogen-inducible transporter in *V. vinifera* (Fung et al., 2008), and more recently by Toth et al. (2016), who identified genes induced by PM colonization but not by SA treatment.

Plant microRNAs (miRNAs) play pivotal roles in plant defense processes (reviewed in Niu et al., 2015; Islam et al., 2018). Han et al. (2016) used high-throughput sequencing of small RNAs to identify miRNAs potentially involved in PM resistance in *V. pseudoreticulata*. They showed that the expression of the highly accumulated vvi-NewmiR2118 miRNA, whose predicted targets are *NBS-LRR* type *R* genes, strongly and rapidly decreases following infection.

#### Guignardia bidwellii

Regarding the interaction between grapevine and *G. bidwellii* or only on the ascomycete itself, there are still no transcriptomic, proteomic or metabolomic data available. A possible reason of this delay could be attributed to the fact that research activities and investments from funding agencies mainly address major diseases (e.g. PM as well as downy mildew). Filling this gap of knowledge in the near future will be very helpful to understand not only the causal agent of BR, but also the molecular events which occur during the infection process.

#### Elsinoë ampelina

Unlike PM, few transcriptomic and proteomic studies have been conducted to elucidate the molecular interactions between *Vitis* species and *E. ampelina* (**Tables 2B**, S2B). A proteomic investigation identified several proteins differentially expressed between tolerant (mid-resistant) and susceptible cultivars, and between uninfected and infected leaves (Vasanthaiah et al., 2009). Two of them, the mitochondrial adenosine triphosphate synthase and glutamine synthetase, were newly expressed in tolerant cultivars upon *E. ampelina* inoculation. Conversely, several proteins, including ribulose-1,5-bisphosphate carboxylase (Rubisco), involved in photosynthesis, were suppressed in infected susceptible genotypes. These results highlighted the ability of the pathogen to significantly affect host physiology, especially in the susceptible cultivars. The tolerant genotypes had the ability to up-regulate and to induce new proteins in order to defend themselves from pathogen invasion and to maintain the normal physiological processes. In tolerant genotypes, a rapid and specific induction of various defense-related genes, including chitinase, stilbene synthase, chalcone synthase, putative proline-rich cell wall protein, thaumatin-like protein, pathogenesis-related (PR) protein 10 and other signal related genes were observed upon *E. ampelina* inoculation (Vasanthaiah et al., 2010; Choi et al., 2011; Louime et al., 2011; Gao et al., 2012b). Most of these genes were strongly induced by the pathogen only in the resistant accessions.

Ahn et al. (2014a)*de novo* assembled the transcriptome of the resistant Korean species *V. flexuosa* inoculated with the pathogen. This analysis led to the identification and subsequent characterization of many resistance related responsive genes to *E. ampelina* inoculation, such as β-1,3-glucanase genes (Ahn et al., 2014b), RPS5-like genes (Islam et al., 2015a), receptor-like protein kinase (RLK) genes (Islam et al., 2015b), Mlo-like genes (Islam and Yun, 2016a), carboxylesterase genes (Islam and Yun, 2016b), glutathione S-transferase genes (Ahn et al., 2016) and EDS1-like genes (Islam and Yun, 2017). Several *V. vinifera* thaumatin-like proteins (*VvTLP*) genes responsive to anthracnose were also recently identified (Yan et al., 2017).

## Discussion

Considering the combination of the three studied diseases, we have concluded that grapevine cultivars resistant to PM and downy mildew are mostly susceptible to BR and AN. This result could suggest a potential compromise of resistance to diseases, but instead can be attributed to the use of a limited source of genetic variation that confers resistance in breeding, particularly when compared with the genetic variability commonly found in grapevine germplasm collections. Secondly, positive selection of resistance to BR and AN is often overlooked, even when parental lines are resistant to one of these diseases in addition to PM. This implies the future proper use of multi-disease resistance sources, considering that potential interactions are not excluded. Indeed, it is currently unknown whether a common resistance mechanism is present in the genus *Vitis*, and if so at what biological scale, in light of the fact that the causal agents are all Ascomycetes. *R*-loci are polymorphic among species, and the upstream recognition phase is specific while a number of downstream mechanisms are common, highlighting that variability is present in the regulation, rather than the mechanisms themselves. In fact, heterologous expression of pathogen-responsive genes mostly confers broad-spectrum resistance, including in fungi of the same family, as well as bacteria. Focusing on single diseases, studies show that the pyramiding of *R*-loci provides an equal or higher degree of disease resistance, which confers long-term ability of overcoming pathogenic invasions.

Ultimately, it will be of paramount importance to characterize genes and pathways underpinning the quantitative PM, BR and AN resistance traits displayed by certain wild *Vitis* species. In order to prove conclusively that these genes do contribute to disease resistance, it will be necessary to demonstrate that the segregation of resistance is genetically linked to the inheritance of these candidate genes. This will also elucidate whether these genes are able to function in different genetic backgrounds i.e. in *V. vinifera*, which will be essential if they are to constitute the basis for any gene pyramiding strategy. Even nowadays, detailed insights about mechanisms underlying gene pyramids are missing–such as redundancy, additive effects or synergistic mechanisms (epistatic effects)–and expression and functional studies on pyramidized materials are very rare or lacking. The adoption of integrated strategies might facilitate the closing of the circle among grapevine/host disciplines (genomics, transcriptomics, and metabolomics).

Multidisciplinary approaches are also crucial and needed for a more comprehensive characterization of the ascomycete/pathogen. The identification of the described Ascomycetes commonly relied on the analysis of their morphological characters and on the observation of the affected plant symptoms, however these parameters are not sufficient for specific pathogen discrimination. The recent advances in high-throughput sequencing technologies and in fungal genomics are opening new possibilities for fungi barcoding. Despite the economic importance of these pathogens, many aspects of their biology have not yet been fully explored because some (i.e. *E. necator*) are impossible to cultivate and propagate *in vitro* due to their obligate biotrophy lifestyle. In this respect, genomic studies, although complicated and challenging, are strongly advised to contribute to identify candidate effector genes involved in the pathogenicity mechanisms. On the other hand, more efforts and funding should be addressed to deepen our understanding of the mechanisms of biotrophy, shedding light onto its molecular and evolutionary basis. So far, to develop artificial media which resembles the host, only empiric strategies could be attempted to design media supplemented with (most probably several) plant molecules able to energetically support the fungus as well as to stimulate its growth.

To face the overall challenge, the vision is that each host discipline will inform the corresponding pathogen discipline, and *vice versa*, based on the practice of multidisciplinary culture.

## Author Contributions

CP, CM, and TCT searched for as well as organized literature and drafted the manuscript. MC searched for literature, drafted the manuscript, created figures and tables, and curated bibliography. PB searched for literature, created figures and tables, and curated bibliography. DP, LW, and LH searched for literature and integrated as well as revised the manuscript. EP, GA, and SM revised the manuscript. LZ searched for and organized literature. MS searched for literature. SV conceptualized as well as coordinated the writing work, searched for literature, and drafted as well as revised the manuscript. All authors read and approved the ﬁnal manuscript.

## Conflict of Interest

The authors declare that the research was conducted in the absence of any commercial or financial relationships that could be construed as a potential conflict of interest.

## References

[B1] Ahn S. Y. Kim S. A. Jo S. H. Yun H. K. (2014a). De novo transcriptome assembly of Vitis flexuosa grapevines inoculated with Elsinoe ampelina. Plant Genet. Resour. 12, S130–S133. 10.1017/S1479262114000410

[B2] Ahn S. Y. Kim S. A. Yun H. K. (2014b). Differential Expression of β -1,3-Glucanase Transcripts Induced by Pathogens in the Leaves of Vitis flexuosa. Plant Breed. Biotechnol. 2, 176–183. 10.9787/PBB.2014.2.2.176

[B3] Ahn S. Y. Kim S. A. Yun H. K. (2016). Glutathione S -transferase genes differently expressed by pathogen-infection in Vitis flexuosa. Plant Breed. Biotechnol. 4, 61–70. 10.9787/PBB.2016.4.1.61

[B4] Akkurt M. Welter L. Maul E. Töpfer R. Zyprian E. (2007). Development of SCAR markers linked to powdery mildew (Uncinula necator) resistance in grapevine (Vitis vinifera L. Mol. Breed. 19, 103–111. 10.1007/s11032-006-9047-9

[B5] Amorim L. L. B. da Fonseca Dos Santos R. Neto J. P. B. Guida-Santos M. Crovella S. Benko-Iseppon A. M. (2017). Transcription factors involved in plant resistance to pathogens. Curr. Protein Pept. Sci. 18, 335–351. 10.2174/1389203717666160619185308 27323805

[B6] Amrani L. Corio-Costet M. F. (2006). A single nucleotide polymorphism in the β-tubulin gene distinguishing two genotypes of Erysiphe necator expressing different symptoms on grapevine. Plant Pathol. 55, 505–512. 10.1111/j.1365-3059.2006.01390.x

[B7] Amrine K. C. H. Blanco-Ulate B. Riaz S. Pap D. Jones L. Figueroa-Balderas R. (2015). Comparative transcriptomics of Central Asian Vitis vinifera accessions reveals distinct defense strategies against powdery mildew. Hortic. Res. 2, 15037. 10.1038/hortres.2015.37 26504579 PMC4591678

[B8] Barker C. L. Donald T. Pauquet J. Ratnaparkhe M. B. Bouquet A. Adam-Blondon A. F. (2005). Genetic and physical mapping of the grapevine powdery mildew resistance gene, Run1, using a bacterial artificial chromosome library. Theor. Appl. Genet. 111, 370–377. 10.1007/s00122-005-2030-8 15902396

[B9] Barnett M. E. (2015). Computational identification of conserved haustorial-expressed genes in the grapevine powdery mildew fungus Erysiphe necator.

[B10] Baroncelli R. Sreenivasaprasad S. Lane C. R. Thon M. R. Sukno S. A. (2014). First report of Colletotrichum acutatum sensu lato (Colletotrichum godetiae) causing anthracnose on grapevine (Vitis vinifera) in the United Kingdom. New Dis. Rep. 29, 26. 0.5197/j.2044-0588.2014.029.026

[B11] Barrett H. C. (1955). Black rot resistance of the foliage on seedlings in selected grape progenies. Proc. Am. Soc Hort. Sci. 66, 220–224.

[B12] Bartlett D. W. Clough J. M. Godwin J. R. Hall A. A. Hamer M. Parr-Dobrzanski B. (2002). The strobilurin fungicides. Pest Manage. Sci. 58, 649–662. 10.1002/ps.520 12146165

[B13] Baudoin A. Olaya G. Delmotte F. Colcol J. F. Sierotzki H. (2008). QoI resistance of Plasmopara viticola and Erysiphe necator in the Mid-Atlantic United States. Plant Heal. Prog. 9, 25. 10.1094/php-2008-0211-02-rs

[B14] Berens M. L. Berry H. M. Mine A. Argueso C. T. Tsuda K. (2017). Evolution of hormone signaling networks in plant defense. Annu. Rev. Phytopathol. 55, 401–425. 10.1146/annurev-phyto-080516-035544 28645231

[B15] Bernier F. Berna A. (2001). Germins and germin-like proteins: Plant do-all proteins. But what do they do exactly? Plant Physiol. Biochem. 39, 545–554. 10.1016/S0981-9428(01)01285-2

[B16] Besselat B. Bouchet J. (1984). Black-rot: situation inquiétante dans certains vignobles. Phytoma-défense des Cult. 356, 33–35.

[B17] Bindschedler L. V. Panstruga R. Spanu P. D. (2016). Mildew-Omics: how global analyses aid the understanding of life and evolution of powdery mildews. Front. Plant Sci. 7, 123. 10.3389/fpls.2016.00123 26913042 PMC4753294

[B18] Blanc S. Wiedemann-Merdinoglu S. Dumas V. Mestre P. Merdinoglu D. (2012). A reference genetic map of Muscadinia rotundifolia and identification of Ren5, a new major locus for resistance to grapevine powdery mildew. Theor. Appl. Genet. 125, 1663–1675. 10.1007/s00122-012-1942-3 22865124

[B19] Bois B. Zito S. Calonnec A. Ollat N. (2017). Climate vs grapevine pests and diseases worldwide: the first results of a global survey. J. Int. des Sci. la Vigne du Vin 51, 133–139. 10.20870/oeno-one.2016.0.0.1780

[B20] Borges A. F. Ferreira R. B. Monteiro S. (2013). Transcriptomic changes following the compatible interaction Vitis vinifera-Erysiphe necator. Plant Physiol. Biochem. PPB 68, 71–80. 10.1016/j.plaphy.2013.03.024 23639450

[B21] Bouquet A. (1986). Introduction dans l’espèce Vitis vinifera L. Vignevini 12, 141–146.

[B22] Bregaglio S. Donatelli M. Confalonieri R. (2013). Fungal infections of rice, wheat, and grape in Europe in 2030-2050. Agron. Sustain. Dev. 33, 767–776. 10.1007/s13593-013-0149-6

[B23] Brent K. J. Hollomon D. W. (2007). “Fungicide resistance: the assessment of risk,” in FRAC Monog (Brussels, Belgium: Published by the Fungicide Resistance Action Committee 2007).

[B24] Brewer M. T. Cadle-Davidson L. Cortesi P. Spanu P. D. Milgroom M. G. (2011). Identification and structure of the mating-type locus and development of PCR-based markers for mating type in powdery mildew fungi. Fungal Genet. Biol. 48, 704–713. 10.1016/j.fgb.2011.04.004 21515399

[B25] CABI, Crop Protection Compendium (2018). Available at: https://www.cabi.org/cpc/

[B26] Cadle-Davidson L. (2018). “A perspective on breeding and implementing durable powdery mildew resistance” in Book of Abstracts of 12th International conference on grapevine breeding and genetics. (Bordeaux, France), 93. Available at: http://gbg2018.u-bordeaux.fr/files/gbg2018/presentation/o60_20180720BordeauxLCD.pdf

[B27] Cadle-Davidson L. Gadoury D. M. Fresnedo-Ramírez J. Yang S. Barba P. Sun Q. et al. (2016). Lessons from a phenotyping center revealed by the genome-guided mapping of powdery mildew resistance loci. Phytopathology 106, 1159–1169. 10.1094/PHYTO-02-16-0080-FI 27135675

[B28] Cadle-Davidson L. Wakefield L. Seem R. C. Gadoury D. M. (2010). Specific isolation of RNA from the grape powdery mildew pathogen Erysiphe necator, an Epiphytic, Obligate Parasite. J. Phytopathol. 158, 69–71. 10.1111/j.1439-0434.2009.01578.x

[B29] Caffarra A. Rinaldi M. Eccel E. Rossi V. Pertot I. (2012). Modelling the impact of climate change on the interaction between grapevine and its pests and pathogens: European grapevine moth and powdery mildew. Agric. Ecosyst. Environ. 148, 89–101. 10.1016/j.agee.2011.11.017

[B30] Caldo R. A. Nettleton D. Peng J. Wise R. P. (2006). Stage-specific suppression of basal defense discriminates barley plants containing fast- and delayed-acting Mla powdery mildew resistance alleles. Mol. Plant-Microbe Interact. 19, 939–947. 10.1094/MPMI-19-0939 16941898

[B31] Caldo R. A. Nettleton D. Wise R. P. (2004). Interaction-dependent gene expression in Mla -specified response to barley powdery mildew. Plant Cell 16, 2514–2528. 10.1105/tpc.104.023382 15319481 PMC520949

[B32] Cannon R. D. Lamping E. Holmes A. R. Niimi K. Baret P. V. Keniya M. V. (2009). Efflux-mediated antifungal drug resistance. Clin. Microbiol. Rev. 22, 291–321. 10.1128/CMR.00051-08 19366916 PMC2668233

[B33] Cartolaro P. Steva H. (1990). Control of powdery mildew in the laboratory. Phytoma 419, 37–40.

[B34] Carver T. L. W. Zeyen R. J. Bushnell W. R. Robbins M. P. (1994). Inhibition of phenylalanine ammonia lyase and cinnamyl alcohol dehydrogenase increases quantitative susceptibility of barley to powdery mildew (Erysiphe graminis D.C.). Physiol. Mol. Plant Pathol. 44, 261–272. 10.1016/S0885-5765(05)80029-3

[B35] Cheng S. Xie X. Xu Y. Zhang C. Wang X. Zhang J. (2016). Genetic transformation of a fruit-specific, highly expressed stilbene synthase gene from Chinese wild Vitis quinquangularis. Planta 243, 1041–1053. 10.1007/s00425-015-2459-1 26781778

[B36] Cherrad S. Charnay A. Hernandez C. Steva H. Belbahri L. Vacher S. (2018). Emergence of boscalid-resistant strains of Erysiphe necator in French vineyards. Microbiol. Res. 216, 79–84. 10.1016/j.micres.2018.08.007 30269859

[B37] Choi Y. J. Ahn S. Y. Kim S. H. Hur Y. Y. Yun H. K. (2011). Profiling transcripts by EST from Vitis coignetiae against Anthracnose Infection. ?국국?농업개발학회지 23, 452–458.

[B38] Coleman C. Copetti D. Cipriani G. Hoffmann S. Kozma P. Kovács L. (2009). The powdery mildew resistance gene REN1 co-segregates with an NBS-LRR gene cluster in two Central Asian grapevines. BMC Genet. 10, 89. 10.1186/1471-2156-10-89 20042081 PMC2814809

[B39] Cools H. J. Fraaije B. A. (2013). Update on mechanisms of azole resistance in Mycosphaerella graminicola and implications for future control. Pest Manage. Sci. 69, 150–155. 10.1002/ps.3348 22730104

[B40] Corio-Costet M. F. Bouscaut J. Delmotte F. Douence L. Richart-Cervera S. Amrani L. (2003). Genetic structure of powdery mildew and fungicide resistance: AFLP and molecular tools of detection. in, “Proceedings 7th ANPP International Conference on Plant Diseases”. University of Agricultural Sciences, GKVK, Bengaluru, India.

[B41] Cortesi P. Ottaviani M. P. Milgroom M. G. (2004). Spatial and genetic analysis of a flag shoot subpopulation of Erysiphe necator in Italy. Phytopathology 94, 544–550. 10.1094/PHYTO.2004.94.6.544 18943478

[B42] Da Silva C. Zamperin G. Ferrarini A. Minio A. Dal Molin A. Venturini L. (2013). The high polyphenol content of grapevine cultivar tannat berries is conferred primarily by genes that are not shared with the reference genome. Plant Cell 25, 4777–4788. 10.1105/tpc.113.118810 24319081 PMC3903987

[B43] Dai L. Wang D. Xie X. Zhang C. Wang X. Xu Y. (2016). The novel gene VpPR4-1 from Vitis pseudoreticulata increases powdery mildew resistance in transgenic Vitis vinifera L. Front. Plant Sci. 7, 695. 10.3389/fpls.2016.00695 27303413 PMC4882328

[B44] Dai L. Xie X. Yang Y. Zhang C. Xu Y. Zhang J. (2017). VpUR9, a novel RING-type ubiquitin ligase gene from Vitis pseudoreticulata, is involved in powdery mildew response in transgenic V. Plant Cell Tissue Organ Cult. 131, 41–49. 10.1007/s11240-017-1260-1

[B45] Dai R. Ge H. Howard S. Qiu W. (2012). Transcriptional expression of Stilbene synthase genes are regulated developmentally and differentially in response to powdery mildew in Norton and Cabernet Sauvignon grapevine. Plant Sci. 197, 70–76. 10.1016/j.plantsci.2012.09.004 23116673

[B46] Dalbó M. A. Weeden N. F. Reisch B. I. (2000). QTL analysis of disease resistance in interspecific hybrid grapes. Acta Hortic. 528, 217–222. 10.17660/ActaHortic.2000.528.29

[B47] Dalbó M. A. Ye G. N. Weeden N. F. Wilcox W. F. Reisch B. I. (2001). Marker-assisted Selection for Powdery Mildew Resistance in Grapes. J. Am. Soc Hortic. Sci. 126, 83–89. 10.21273/JASHS.126.1.83

[B48] De Waard M. A. Andrade A. C. Hayashi K. Schoonbeek H. J. Stergiopoulos I. Zwiers L. H. (2006). Impact of fungal drug transporters on fungicide sensitivity, multidrug resistance and virulence. Pest Manage. Sci. 62, 195–207. 10.1002/ps.1150 16475240

[B49] Delmotte F. Guimier S. Demeaux I. Couture C. Schneider C. Cailliatte R. (2018). “OSCAR, a national observatory to support the deployment of new grapevine disease-resistant varieties in France,” in Book of Abstracts of the XII International Conference on Grapevine Breeding and Genitics., Bordeaux, France, 15-20 July 2018, 29.

[B50] Délye C. Bousset L. Corio-Costet M. F. (1998). PCR cloning and detection of point mutations in the eburicol 14α-demethylase (CYP51) gene from Erysiphe graminis f. Curr. Genet. 34, 399–403. 10.1007/s002940050413 9871123

[B51] Délye C. Corio-Costet M. F. (1998). Origin of primary infections of grape by Uncinula necator: RAPD analysis discriminates two biotypes. Mycol. Res. 102, 283–288. 10.1017/S0953756297004632

[B52] Délye C. Laigret F. Corio-Costet M. F. (1997a). A mutation in the 14αd-Demethylase gene of Uncinula necator that correlates with resistance to a sterol biosynthesis inhibitor. Appl. Environ. Microbiol. 63, 2966–2970. 9251183 10.1128/aem.63.8.2966-2970.1997 PMC168594

[B53] Délye C. Laigret F. Corio-Costet M. F. (1997b). RAPD analysis provides insight into the biology and epidemiology of Uncinula necator. Phytopathology 87, 670–677. 10.1094/PHYTO.1997.87.7.670 18945087

[B54] Délye C. Ronchi V. Laigret F. Corio-Costet M. F. (1999). Nested allele-specific PCR primers distinguish genetic groups of Uncinula necator. Appl. Environ. Microbiol. 65, 3950–3954. 10473400 10.1128/aem.65.9.3950-3954.1999 PMC99725

[B55] Demaree J. B. Dix I. W. Magoon C. A. (1937). Observations on the resistance of grape varieties to black rot and downy mildew. Proc. Am. Soc Hortic. Sci. 35, 451–460.

[B56] Deokate A. S. Khilare V. C. Gangawane L. V. (2002). Resistance to carbendazim in Gloeosporium ampelophagum (Pass) Sacc. Indian J. Plant Prot. 30, 69–70.

[B57] Dry I. B. Feechan A. Anderson C. Jermakow A. M. Bouquet A. Adam-Blondon A. F. (2010). Molecular strategies to enhance the genetic resistance of grapevines to powdery mildew. Aust. J. Grape Wine Res. 16, 94–105. 10.1111/j.1755-0238.2009.00076.x

[B58] Dufour M. C. Fontaine S. Montarry J. Corio-Costet M. F. (2011). Assessment of fungicide resistance and pathogen diversity in Erysiphe necator using quantitative real-time PCR assays. Pest Manage. Sci. 67, 60–69. 10.1002/ps.2032 20949585

[B59] Dutech C. Enjalbert J. Fournier E. Delmotte F. Barrès B. Carlier J. (2007). Challenges of microsatellite isolation in fungi. Fungal Genet. Biol. 44, 933–949. 10.1016/j.fgb.2007.05.003 17659989

[B60] Ellis M. A. (1986). Electronic Grape Black Rot Predictor for Scheduling Fungicides with Curative Activity. Plant Dis. 70, 938. 10.1094/pd-70-938

[B61] Evans K. J. Whisson D. L. Stummer B. E. Scott E. S. (1997). DNA markers identify variation in Australian populations of Uncinula necator. Mycol. Res. 101, 923–932. 10.1017/S0953756297003596

[B62] Falacy J. S. Grove G. Mahaffee Wagriculture F. Galloway H. Glawe D. A. Larsen R. C. (2007). Detection of Erysiphe necator in air samples using the polymerase chain reaction and species-specific primers. Phytopathology 97, 1290–1297. 10.1094/PHYTO-97-10-1290 18943687

[B63] Fan X. L. Barreto R. W. Groenewald J. Z. Bezerra J. D. P. Pereira O. L. Cheewangkoon R. (2017). Phylogeny and taxonomy of the scab and spot anthracnose fungus Elsinoë (Myriangiales, Dothideomycetes). Stud. Mycol. 87, 1–41. 10.1016/j.simyco.2017.02.001 28373739 PMC5367849

[B64] FAO (2016). The state of food and agriculture. Climate Change Agricolture Food Secur. Available at: http://www.fao.org/3/I9553EN/i9553en.pdf

[B65] Feechan A. Anderson C. Torregrosa L. Jermakow A. Mestre P. Wiedemann-Merdinoglu S. (2013). Genetic dissection of a TIR-NB-LRR locus from the wild North American grapevine species Muscadinia rotundifolia identifies paralogous genes conferring resistance to major fungal and oomycete pathogens in cultivated grapevine. Plant J. 76, 661–674. 10.1111/tpj.12327 24033846

[B66] Feechan A. Kocsis M. Riaz S. Zhang W. Gadoury D. M. Walker M. A. (2015). Strategies for RUN1 Deployment Using RUN2 and REN2 to manage grapevine powdery mildew informed by studies of race specificity. Phytopathology 105, 1104–1113. 10.1094/PHYTO-09-14-0244-R 26039639

[B67] Fekete C. Fung R. W. M. Szabó Z. Qiu W. Chang L. Schachtman D. P. (2009). Up-regulated transcripts in a compatible powdery mildew–grapevine interaction. Plant Physiol. Biochem. 47, 732–738. 10.1016/j.plaphy.2009.03.006 19362490

[B68] Fennell J. L. (1948). Inheritance studies with the tropical grape. J. Hered. 39, 54–66. 10.1093/oxfordjournals.jhered.a105800

[B69] Ferrin D. M. (1976). Epidemiological studies of the dispersal of and infection by Guignardia bidwellii (Ellis) Viala and Ravaz, the causal agent of black rot disease of “Concord” and “Niagara” grapes, Vitis labrusca L.

[B70] Ferrin D. M. Ramsdell D. C. (1977). Ascospore dispersal and infection of grapes by Guignardia bidwellii, the causal agent of grape black rot dDisease. Phytopathology 77, 1501. 10.1094/Phyto-67-1501

[B71] Ferrin D. M. Ramsdell D. C. (1978). Influence of Conidia dispersal and environment on infection of grape by Guignardia bidwellii. Phytopathology 68, 892–895. 10.1094/Phyto-68-892

[B72] Foria S. Monte C. Testolin R. Di Gaspero G. Cipriani G. (2019). Piramydizing resistance genes in grape: a breeding program for the selection of “elite” cultivars, Acta Hortic. 1248, 549–554. 10.17660/ActaHortic.2019.1248.73

[B73] FRAC (2019). Available at: https://www.frac.info/

[B74] Frenkel O. Cadle-Davidson L. Wilcox W. F. Milgroom M. G. (2015). Mechanisms of resistance to an Azole fungicide in the grapevine powdery mildew fungus, Erysiphe necator. Phytopathology 105, 370–377. 10.1094/PHYTO-07-14-0202-R 25271353

[B75] Frenkel O. Portillo I. Brewer M. T. Péros J. P. Cadle-Davidson L. Milgroom M. G. (2012). Development of microsatellite markers from the transcriptome of Erysiphe necator for analysing population structure in North America and Europe. Plant Pathol. 61, 106–119. 10.1111/j.1365-3059.2011.02502.x

[B76] Fu Y. Zhang H. Mandal S. N. Wang C. Chen C. Ji W. (2016). Quantitative proteomics reveals the central changes of wheat in response to powdery mildew. J. Proteomics 130, 108–119. 10.1016/j.jprot.2015.09.006 26381202

[B77] Fung R. W. M. Gonzalo M. Fekete C. Kovacs L. G. He Y. Marsh E. (2008). Powdery mildew induces defense-oriented reprogramming of the transcriptome in a susceptible but not in a resistant grapevine. Plant Physiol. 146, 236–249. 10.1104/pp.107.108712 17993546 PMC2230561

[B78] Fung R. W. M. Qiu W. Su Y. Schachtman D. P. Huppert K. Fekete C. (2007). Gene expression variation in grapevine species Vitis vinifera L. Genet. Resour. Crop Evol. 54, 1541–1553. 10.1007/s10722-006-9146-9

[B79] Gadoury D. M. Cadle-Davidson L. Wilcox W. F. Dry I. B. Seem R. C. Milgroom M. G. (2012). Grapevine powdery mildew (Erysiphe necator): a fascinating system for the study of the biology, ecology and epidemiology of an obligate biotroph. Mol. Plant Pathol. 13, 1–16. 10.1111/j.1364-3703.2011.00728.x 21726395 PMC6638670

[B80] Gao F. Shu X. Ali M. B. Howard S. Li N. Winterhagen P. (2010). A functional EDS1 ortholog is differentially regulated in powdery mildew resistant and susceptible grapevines and complements an Arabidopsis eds1 mutant. Planta 231, 1037–1047. 10.1007/s00425-010-1107-z 20145949

[B81] Gao M. Niu J. Zhao S. Jiao C. Xu W. Fei Z. (2012a). Characterization of Erysiphe necator-responsive genes in Chinese Wild Vitis quinquangularis. Int. J. Mol. Sci. 13, 11497–11519. 10.3390/ijms130911497 23109867 PMC3472759

[B82] Gao M. Wang Q. Wan R. Fei Z. Wang X. (2012b). Identification of genes differentially expressed in grapevine associated with resistance to Elsinoe ampelina through suppressive subtraction hybridization. Plant Physiol. Biochem. 58, 253–268. 10.1016/j.plaphy.2012.07.009 22864229

[B83] Gao Y.-R. Han Y.-T. Zhao F.-L. Li Y.-J. Cheng Y. Ding Q. (2016). Identification and utilization of a new Erysiphe necator isolate NAFU1 to quickly evaluate powdery mildew resistance in wild Chinese grapevine species using detached leaves. Plant Physiol. Biochem. 98, 12–24. 10.1016/j.plaphy.2015.11.003 26590705

[B84] Gee C. T. Gadoury D. M. Cadle-Davidson L. (2008). Ontogenic resistance to Uncinula necator varies by genotype and tissue type in a diverse collection of Vitis spp. Plant Dis. 92, 1067–1073. 10.1094/pdis-92-7-1067 30769521

[B85] Gisi U. Sierotzki H. Cook A. McCaffery A. (2002). Mechanisms influencing the evolution of resistance to Qo inhibitor fungicides. Pest Manage. Sci. 58, 859–867. 10.1002/ps.565 12233175

[B86] Godfrey D. Able A. J. Dry I. B. (2007). Induction of a grapevine germin-like protein (VvGLP3) gene is closely linked to the site of Erysiphe necator infection: a possible role in defense? Mol. Plant Microbe. Interact. 20, 1112–1125. 10.1094/MPMI-20-9-1112 17849714

[B87] Goyal N. Bhatia G. Sharma S. Garewal N. Upadhyay A. Upadhyay S. K. (2019). Genome-wide characterization revealed role of NBS-LRR genes during powdery mildew infection in Vitis vinifera. Genomics. 10.1016/j.ygeno.2019.02.011 30802599

[B88] Guan X. Zhao H. Xu Y. Wang Y. (2011). Transient expression of glyoxal oxidase from the Chinese wild grape Vitis pseudoreticulata can suppress powdery mildew in a susceptible genotype. Protoplasma 248, 415–423. 10.1007/s00709-010-0162-4 20512385

[B89] Gubler W. D. Ypema H. L. Ouimette D. G. Bettiga L. J. (1996). Occurrence of resistance in Uncinula necator to triadimefon, myclobutanil, and fenarimol in California grapevines. Plant Dis. 80, 902–909. 10.1094/PD-80-0902

[B90] Guginski-Piva C. A. Bogo A. Gomes B. R. Menon J. K. Nodari R. O. Welter L. J. (2018). Morphological and molecular characterization of Colletotrichum nymphaeae and C. J. Plant Dis. Prot. 125, 405–413. 10.1007/s41348-018-0176-2

[B91] Hacquard S. (2014). “Chapter Four - The Genomics of Powdery Mildew Fungi: Past Achievements, Present Status and Future Prospects,” in Advances in Botanical Research (Birmingham, West Midlands, England: Academic Press), 109–142. 10.1016/B978-0-12-397940-7.00004-5

[B92] Hajjeh H. Miazzi M. De Guido M. A. Faretra F. (2005). Specific SCAR primers for the “flag shoot” and “ascospore” biotypes of the grape powdery mildew fungus Erysiphe necator. J. Plant Pathol. 87, 71–74. 10.4454/jpp.v87i1.899

[B93] Hamamoto H. Hasegawa K. Nakaune R. Lee Y. J. Makizumi Y. Akutsu K. (2000). Tandem repeat of a transcriptional enhancer upstream of the sterol 14α-demethylase gene (CYP51) in Penicillium digitatum. Appl. Environ. Microbiol. 66, 3421–3426. 10.1128/AEM.66.8.3421-3426.2000 10919801 PMC92165

[B94] Han L. Weng K. Ma H. Xiang G. Li Z. Wang Y. (2016). Identification and characterization of Erysiphe necator-responsive MicroRNAs in Chinese wild Vitis pseudoreticulata by high-throughput sequencing. Front. Plant Sci. 7, 621. 10.3389/fpls.2016.00621 27303408 PMC4885880

[B95] Hanif M. Rahman M. Gao M. Yang J. Ahmad B. Yan X. (2018). Heterologous xxpression of the grapevine JAZ7 gene in Arabidopsis confers enhanced resistance to powdery mildew but not to Botrytis cinerea. Int. J. Mol. Sci. 19, 3889. 10.3390/ijms19123889 PMC6321488 30563086

[B96] Harms M. Holz G. Hoffmann C. Lipps H.-P. Silvanus W. (2005). Occurrence of Guignardia bidwellii, the causal fungus of black rot on grapevine, in the vine growing areas of Rhineland-Palatinate, Germany M Harms. BCPC Symp., 127–132.

[B97] Hausmann L. Rex F. Töpfer R. (2017). Evaluation and genetic analysis of grapevine black rot resistances. Acta Hortic. 1188, 285–290. 10.17660/ActaHortic.2017.1188.37

[B98] Hausmann L. Maul E. Ganesch A. Töpfer R. (2019). Overview of genetic loci for traits in grapevine and their integration into the VIVC database. Acta Hortic. 1248, 221–226. 10.17660/ActaHortic.2019.1248.32

[B99] Hayashi K. Schoonbeek H. J. De Waard M. A. (2002). Expression of the ABC transporter BcatrD from Botrytis cinerea reduces sensitivity to sterol demethylation inhibitor fungicides. Pestic. Biochem. Physiol. 73, 110–121. 10.1016/S0048-3575(02)00015-9

[B100] Higgins S. I. Scheiter S. (2012). Atmospheric CO2 forces abrupt vegetation shifts locally, but not globally. Nature 488, 209–212. 10.1038/nature11238 22763447

[B101] Hoffman L. E. Wilcox W. F. (2002). Utilizing epidemiological investigations to optimize management of grape black rot. Phytopathology 92, 676–680. 10.1094/PHYTO.2002.92.6.676 18944268

[B102] Hoffman L. E. Wilcox W. F. Gadoury D. M. Seem R. C. (2002). Influence of grape berry age on susceptibility to Guignardia bidwellii and its incubation period length. Phytopathology 92, 1068–1076. 10.1094/PHYTO.2002.92.10.1068 18944217

[B103] Hoffman L. E. Wilcox W. F. Gadoury D. M. Seem R. C. Riegel D. G. (2004). Integrated control of grape black rot: influence of host phenology, inoculum availability, sanitation, and spray timing. Phytopathology 94, 641–650. 10.1094/PHYTO.2004.94.6.641 18943489

[B104] Hoffmann S. Di Gaspero G. Kovács L. Howard S. Kiss E. Galbács Z. (2008). Resistance to Erysiphe necator in the grapevine “Kishmish vatkana” is controlled by a single locus through restriction of hyphal growth. Theor. Appl. Genet. 116, 427–438. 10.1007/s00122-007-0680-4 18064436

[B105] Hollomon D. W. (2015). Fungicide resistance: facing the challenge. Plant Prot. Sci. 51, 170–176. 10.17221/42/2015-PPS

[B106] Hopkins D. L. Harris J. W. (2000). A greenhouse method for screening grapevine seedlings for resistance to Anthracnose. HortScience 35, 89–91. 10.21273/HORTSCI.35.1.89

[B107] Huang Y. (2016). Assessing the genetic diversity of grape ripe rot pathogen Colletotrichum using SRAP markers. Mycosphere 7, 1103–1110. 10.5943/mycosphere/si/2c/3

[B108] Hyun J.-W. Timmer L. W. Lee S.-C. Yun S.-H. Ko S.-W. Kim K.-S. (2001). Pathological characterization and molecular analysis of Elsinoe isolates causing scab diseases of citrus in Jeju Island in Korea. Plant Dis. 85, 1013–1017. 10.1094/PDIS.2001.85.9.1013 30823084

[B109] Islam M. Z. Ahn S. Y. Yun H. K. (2015a). Analysis of structure and differential expression of Pseudomonas syringae 5-like(RPS5-like) genes in pathogen-infected Vitis flexuosa. TURKISH J. Biol. 39, 775–789. 10.3906/biy-1502-38

[B110] Islam M. Z. Ahn S. Y. Yun H. K. (2015b). Identification of six transcripts encoding putative receptor-like protein kinase (RLK) and their expression profiles in Vitis flexuosa infected with pathogens. Sci. Hortic. (Amsterdam) 192, 108–116. 10.1016/j.scienta.2015.05.025

[B111] Islam M. Z. Yun H. (2016a). Characterization of nine Mlo family genes and analysis of their expression against pathogen infections in Vitis flexuosa. Euphytica 211, 379–394. 10.1007/s10681-016-1752-9

[B112] Islam M. Z. Yun H. K. (2016b). Identification and expression profiles of six transcripts encoding carboxylesterase protein in Vitis flexuosa infected with pathogens. Plant Pathol. J. 32, 347–356. 10.5423/PPJ.OA.11.2015.0241 27493610 PMC4968645

[B113] Islam M. Z. Yun H. K. (2017). Three transcripts of EDS1-like genes respond differently to Vitis flexuosa infection. J. Plant Biotechnol. 44, 125–134. 10.5010/JPB.2017.44.2.125

[B114] Islam W. Noman A. Qasim M. Wang L. (2018). Plant responses to pathogen attack: small RNAs in focus. Int. J. Mol. Sci. 19. 10.3390/ijms19020515 PMC5855737 29419801

[B115] Jabco J. Nesbitt W. Werner D. (1985). Resistance of various classes of grapes to the bunch and muscadine grape forms of Black Rot. J. Am. Soc Hortic. Sci. 110, 762–765.

[B116] Jacobs A. K. Dry I. B. Robinson S. P. (1999). Induction of different pathogenesis-related cDNAs in grapevine infected with powdery mildew and treated with ethephon. Plant Pathol. 48, 325–336. 10.1046/j.1365-3059.1999.00343.x

[B117] Jailloux F. (1992). In vitro production of the teleomorph of Guignardia bidwellii, causal agent of black rot of grapevine. Can. J. Bot. 70, 254–257. 10.1139/b92-035

[B118] Jang M. H. Ahn S. Y. Kim S. H. Noh J. H. Yun H. K. (2011). Evaluation of grapevine varietal resistance to anthracnose through treating culture filtrates from Elsinoe ampelina. Hortic. Environ. Biotechnol. 52, 152–157. 10.1007/s13580-011-0107-7

[B119] Jayasankar S. Li Z. Gray D. J. (2000). In-vitro selection of Vitis vinifera `Chardonnay’ with Elsinoe ampelina culture filtrate is accompanied by fungal resistance and enhanced secretion of chitinase. Planta 211, 200–208. 10.1007/s004250000285 10945214

[B120] Jiao C. Gao M. Wang X. Fei Z. (2015). Transcriptome characterization of three wild Chinese Vitis uncovers a large number of distinct disease related genes. BMC Genomics 16, 223. 10.1186/s12864-015-1442-3 25888081 PMC4373064

[B121] Jiao Y. Wang D. Wang L. Jiang C. Wang Y. (2017). VqMAPKKK38 is essential for stilbene accumulation in grapevine. Hortic. Res. 4, 17058. 10.1038/hortres.2017.58 29051820 PMC5645558

[B122] Jiao Y. Xu W. Duan D. Wang Y. Nick P. (2016). A stilbene synthase allele from a Chinese wild grapevine confers resistance to powdery mildew by recruiting salicylic acid signalling for efficient defence. J. Exp. Bot. 67, 5841–5856. 10.1093/jxb/erw351 27702992 PMC5066501

[B123] Jones L. Riaz S. Morales-Cruz A. Amrine K. C. H. McGuire B. Gubler W. D. (2014). Adaptive genomic structural variation in the grape powdery mildew pathogen, Erysiphe necator. BMC Genomics 15, 1081. 10.1186/1471-2164-15-1081 25487071 PMC4298948

[B124] Joshi R. K. Nayak S. (2010). Gene pyramiding-A broad spectrum technique for developing durable stress resistance in crops. Biotechnol. Mol. Biol. Rev. 5, 51–60.

[B125] Kim G. H. Yun H. K. Choi C. S. Park J. H. Jung Y. J. Park K. S. (2008). Identification of AFLP and RAPD markers linked to anthracnose resistance in grapes and their conversion to SCAR markers. Plant Breed. 127, 418–423. 10.1111/j.1439-0523.2008.01488.x

[B126] Koch E. Enders M. Ullrich C. Molitor D. Berkelmann-Löhnertz B. (2013). Effect of Primula root and other plant extracts on infection structure formation of Phyllosticta ampelicida (asexual stage of Guignardia bidwellii) and on black rot disease of grapevine in the greenhouse. J. Plant Dis. Prot. 120, 26–33. 10.1007/BF03356450

[B127] Kong G. (2009). Diagnostic methods for black rot of grapes. PaDIL Plant Biosecur. Toolbox 28.

[B128] Kono A. Nakaune R. Yamada M. Nakano M. Mitani N. Ueno T. (2009). Effect of culture conditions on Conidia formation by Elsinoë ampelina, the causal organism of grapevine anthracnose. Plant Dis. 93, 481–484. 10.1094/PDIS-93-5-0481 30764135

[B129] Kono A. Sato A. Nakano M. Yamada M. Mitani N. Ban Y. (2012). Evaluating grapevine cultivars for resistance to anthracnose based on lesion number and length. Am. J. Enol. Vitic. 63, 262–268. 10.5344/ajev.2012.11109

[B130] Kretschmer M. Leroch M. Mosbach A. Walker A. S. Fillinger S. Mernke D. (2009). Fungicide-driven evolution and molecular basis of multidrug resistance in field populations of the grey mould fungus Botrytis cinerea. PloS Pathog. 5. 10.1371/journal.ppat.1000696 PMC2785876 20019793

[B131] Kunova A. Pizzatti C. Bonaldi M. Cortesi P. (2016). Metrafenone resistance in a population of Erysiphe necator in northern Italy. Pest Manage. Sci. 72, 398–404. 10.1002/ps.4060 PMC5034827 26079510

[B132] Kuo K. Hoch H. C. (1996). The parasitic relationship between Phyllosticta ampelicida and Vitis vinifera. Mycologia 88, 626. 10.2307/3761158

[B133] Kwon J.-H. Choi O. Kang D.-W. Kim W.-I. Kim J. (2015). Guignardia bidwellii causes leaf spot on Boston ivy in South Korea. Australas. Plant Dis. Notes 10, 20. 10.1007/s13314-015-0172-3

[B134] Lafon R. Bugaret Y. Bulit J. Clerjau M. (1984). Results of 5 years viticultural trials to control the most important fungus diseases with the combination of aluminiumfosetyl and folpet in France. Wein Wiss. 29, 226–242.

[B135] Leroux P. Albertini C. Gautier A. Gredt M. Walker A. (2007). Mutations in the CYP51 gene correlated with changes in sensitivity to sterol 14α-demethylation inhibitors in field isolates of Mycosphaerella graminicola. Pest Manage. Sci. Former. Pestic. Sci. 63, 688–698. 10.1002/ps.1390 17511023

[B136] Leroux P. Walker A. S. (2011). Multiple mechanisms account for resistance to sterol 14α-demethylation inhibitors in field isolates of Mycosphaerella graminicola. Pest Manage. Sci. 67, 44–59. 10.1002/ps.2028 20949586

[B137] Li D. Wan Y. Wang Y. He P. (2008). Relatedness of resistance to anthracnose and to white rot in Chinese wild grapes. Vitis - J. Grapevine Res. 47, 213–215.

[B138] Li H. (1993). Studies on the resistance of grapevine to powdery mildew. Plant Pathol. 42, 792–796. 10.1111/j.1365-3059.1993.tb01566.x

[B139] Li H. Xu Y. Xiao Y. Zhu Z. Xie X. Zhao H. (2010). Expression and functional analysis of two genes encoding transcription factors, VpWRKY1 and VpWRKY2, isolated from Chinese wild Vitis pseudoreticulata. Planta 232, 1325–1337. 10.1007/s00425-010-1258-y 20811906

[B140] Liu M. Zhang W. Zhou Y. Liu Y. Yan J. Y. Li X. H. (2016). First report of twig anthracnose on grapevine caused by Colletotrichum nymphaeae in China. Plant Dis. 100, 2530–2530. 10.1094/PDIS-05-16-0632-PDN

[B141] Loskill B. Molitor D. Koch E. Harms M. Berkelmann-Löhnertz B. Hoffmann C. (2009). Strategien zur Regulation der Schwarzfäule (Guignardia bidwellii) im ökologischen Weinbau. Available at: http://orgprints.org/17072/1/17072-04OE032-jki-maixner-2009-schwarzfaeule.pdf

[B142] Louime C. Lu J. Onokpise O. Vasanthaiah H. K. N. Kambiranda D. Basha S. M. (2011). Resistance to Elsinoë ampelina and expression of related resistant genes in Vitis rotundifolia Michx. Int. J. Mol. Sci. 12, 3473–3488. 10.3390/ijms12063473 21747689 PMC3131573

[B143] Luo C. X. Cox K. D. Amiri A. Schnabel G. (2008). Occurrence and detection of the DMI resistance-associated genetic element “Mona” in Monilinia fructicola. Plant Dis. 92, 1099–1103. 10.1094/PDIS-92-7-1099 30769523

[B144] Lupetti A. Danesi R. Campa M. Del Tacca M. Kelly S. (2002). Molecular basis of resistance to azole antifungals. Trends Mol. Med. 8, 76–81. 10.1016/S1471-4914(02)02280-3 11815273

[B145] Luttrell E. S. (1946). Black rot of muscadine grapes. Phytopathology 36, 905–924.

[B146] Luttrell E. S. (1948). Physiologic specialization in Guignardia bidwellii, cause of black rot of Vitis and Parthenocissus species. Phytopathology 38, 716–723.

[B147] Luttrell E. S. (1974). Parasitism of fungi on Vascular Plants. Mycologia 66, 1–15. 10.1080/00275514.1974.12019567

[B148] Ma Z. Michailides T. J. (2005). Advances in understanding molecular mechanisms of fungicide resistance and molecular detection of resistant genotypes in phytopathogenic fungi. Crop Prot. 24, 853–863. 10.1016/j.cropro.2005.01.011

[B149] Ma Z. Proffer T. J. Jacobs J. L. Sundin G. W. (2006). Overexpression of the 14α-demethylase target gene (CYP51) mediates fungicide resistance in Blumeriella jaapii. Appl. Environ. Microbiol. 72, 2581–2585. 10.1128/AEM.72.4.2581-2585.2006 16597960 PMC1449055

[B150] Magarey R. D. Coffey B. E. Emmett R. W. (1993a). Anthracnose of grapevines, a review. Plant Prot. Q. 8, 106–110.

[B151] Magarey R. D. Emmett R. W. Magarey P. A. Franz P. R. (1993b). Evaluation of control of grapevine anthracnose caused by Eisinoe ampelina by pre-infection fungicides. Australas. Plant Pathol. 22, 48–52. 10.1071/APP9930048

[B152] Mahanil S. Ramming D. Cadle-Davidson M. Owens C. Garris A. Myles S. (2012). Development of marker sets useful in the early selection of Ren4 powdery mildew resistance and seedlessness for table and raisin grape breeding. Theor. Appl. Genet. 124, 23–33. 10.1007/s00122-011-1684-7 21904846

[B153] Marchive C. Mzid R. Deluc L. Barrieu F. Pirrello J. Gauthier A. (2007). Isolation and characterization of a Vitis vinifera transcription factor, VvWRKY1, and its effect on responses to fungal pathogens in transgenic tobacco plants. J. Exp. Bot. 58, 1999–2010. 10.1093/jxb/erm062 17456504

[B154] Marsh E. Alvarez S. Hicks L. M. Barbazuk W. B. Qiu W. Kovacs L. (2010). Changes in protein abundance during powdery mildew infection of leaf tissues of Cabernet Sauvignon grapevine (Vitis vinifera L.). Proteomics 10, 2057–2064. 10.1002/pmic.200900712 20232356

[B155] Mathukorn S. (2012). Morphological, pathogenicity and virulence characterization of Sphaceloma ampelinum the causal agent of grape anthracnose in Thailand. Afr. J. Microbiol. Res. 6, 2313–2320. 10.5897/AJMR11.1149

[B156] Maul E. Sudharma K. N. Kecke S. Marx G. Müller C. Audeguin L. (2012). The European Vitis Database (www.eu-vitis.de): A technical innovation through an online uploading and interactive modification system. Vitis 51, 79–85.

[B157] McGovern P. E. (2004). Ancient Wine: The Search for the Origins of Viniculture. Princeton, New Jersey: Princeton University Press.

[B158] Merdinoglu D. Schneider C. Prado E. Wiedemann-Merdinoglu S. Mestre P. (2018). Breeding for durable resistance to downy and powdery mildew in grapevine. OENO One 52, 203–209. 10.20870/oeno-one.2018.52.3.2116

[B159] Miazzi M. Hajjeh H. Faretra F. (2008). Occurrence and distribution of two distinct genetic groups in populations of Erisiphe necator schw. J. Plant Pathol. 90, 563–573. 10.4454/jpp.v90i3.701

[B160] Miclot A. Wiedemann-Merdinoglu S. Duchêne E. Merdinoglu D. Mestre P. (2012). A standardised method for the quantitative analysis of resistance to grapevine powdery mildew. Eur. J. Plant Pathol. 133, 483–495. 10.1007/s10658-011-9922-z

[B161] Miedaner T. (2016). “Breeding Strategies for Improving Plant Resistance to Diseases,” in Advances in Plant Breeding Strategies: Agronomic, Abiotic and Biotic Stress Traits (Cham: Springer International Publishing), 561–599. 10.1007/978-3-319-22518-0_15

[B162] Miessner S. Mann W. Stammler G. (2011). Guignardia bidwellii, The causal agent of black rot on grapevine has a low risk for QoI resistance. J. Plant Dis. Prot. 118, 51–53. 10.1007/BF03356381

[B163] Miles L. A. Miles T. D. Kirk W. W. Schilder A. M. C. (2012). Strobilurin (QoI) resistance in populations of Erysiphe necator on grapes in Michigan. Plant Dis. 96, 1621–1628. 10.1094/PDIS-01-12-0041-RE 30727456

[B164] Molitor D. Augenstein B. Mugnai L. Rinaldi P. A. Sofia J. Hed B. (2016). Composition and evaluation of a novel web-based decision support system for grape black rot control. Eur. J. Plant Pathol. 144, 785–798. 10.1007/s10658-015-0835-0

[B165] Molitor D. Berkelmann-Löhnertz B. (2011). Simulating the susceptibility of clusters to grape black rot infections depending on their phenological development. Crop Prot. 30, 1649–1654. 10.1016/j.cropro.2011.07.020

[B166] Molitor D. Beyer M. (2014). Epidemiology, identification and disease management of grape black rot and potentially useful metabolites of black rot pathogens for industrial applications - a review. Ann. Appl. Biol. 165, 305–317. 10.1111/aab.12155

[B167] Molitor D. Fruehauf C. Baus O. Berkelmann-Löhnertz B. (2012). A cumulative degree-day-based model to calculate the duration of the incubation period of Guignardia bidwellii. Plant Dis. 96, 1054–1059. 10.1094/PDIS-11-11-1005-RE 30727216

[B168] Mortensen J. A. (1977). Segregation for resistance to black rot in selfed grape seedlings. Fruit Var. J. 31, 59–60.

[B169] Mortensen J. A. (1981). Sources and inheritance of resistance to anthracnose in Vitis. J. Hered. 72, 423–426. 10.1093/oxfordjournals.jhered.a109545

[B170] Moyer M. M. Gadoury D. M. Cadle-Davidson L. Dry I. B. Magarey P. A. Wilcox W. F. (2010). Effects of acute low-temperature events on development of Erysiphe necator and susceptibility of Vitis vinifera. Phytopathology 100, 1240–1249. 10.1094/phyto-01-10-0012 20649419

[B171] Muthmann R. Nardin P. (2007). The use of plant protection products in the European Union: Data 1992–2003. Office for Official Publications of the European Communities, Luxembourg.

[B172] Myers J. (2012). Assembly, annotation, and polymorphic characterization of the Erysiphe necator transcriptome. Diss. thesis. Available at: http://scholarworks.rit.edu/theses/4077

[B173] Mzid R. Marchive C. Blancard D. Deluc L. Barrieu F. Corio-Costet M. F. (2007). Overexpression of VvWRKY2 in tobacco enhances broad resistance to necrotrophic fungal pathogens. Physiol. Plant 131, 434–447. 10.1111/j.1399-3054.2007.00975.x 18251882

[B174] Narduzzi-Wicht B. Jermini M. Gessler C. Broggini G. A. L. (2014). Microsatellite markers for population studies of the ascomycete Phyllosticta ampelicida, the pathogen causing grape black rot. Phytopathol. Mediterr. 53, 470–479. 10.14601/phytopathol_mediterr-14481

[B175] Ng D. W.-K. Abeysinghe J. K. Kamali M. (2018). Regulating the regulators: the control of transcription factors in plant defense signaling. Int. J. Mol. Sci. 19. 10.3390/ijms19123737 PMC6321093 30477211

[B176] Niu D. Wang Z. Wang S. Qiao L. Zhao H. (2015). Profiling of small RNAs involved in plant-pathogen interactions. Methods Mol. Biol. 1287, 61–79. 10.1007/978-1-4939-2453-0_4 25740356

[B177] Northover P. R. (2008). Factors Influencing the Infection of Cultivated Grape (Vitis spp. Section Euvitis) Shoot Tissue by Guignardia bidwellii (Ellis) Viala & Ravaz.

[B178] Núñez Y. Gallego J. Ponz F. Raposo R. (2006). Analysis of population structure of Erysiphe necator using AFLP markers. Plant Pathol. 55, 650–656. 10.1111/j.1365-3059.2006.01435.x

[B179] OEPP/EPPO (2001). Guidelines for the efficacy evaluation of fungicides. OEPP/EPPO Bullettin 31, 313–317.

[B180] Ogawa J. M. Gubler W. D. Manji B. T. (1988). Effect of sterol biosynthesis inhibitors on diseases of stone fruits and grapes in California. Sterol Biosynth. Inhib. Pharm. Agrochem. Asp. by D. B. M. Plempel.

[B181] OIV (2009). Descriptor list for grape varieties and Vitis species. Paris: Office International de la Vigne et du Vin Available at: http://www.oiv.int/public/medias/2274/code-2e-edition-finale.pdf

[B182] Oliveira M. Cunha M. (2015). Study of the portuguese populations of powdery mildew fungus from diverse grapevine cultivars (Vitis vinifera). OENO One 49, 173. 10.20870/oeno-one.2015.49.3.83

[B183] Oliver R. P. Hewitt H. G. (2014). “Chapter 5: Fungicide performance,” in Fungicides in crop protection (Wallingford, UK: CABI International), 71–122. Available at: https://espace.curtin.edu.au/handle/20.500.11937/10045

[B184] Olmo H. P. (1971). Vinifera Rotundifolia Hybrids as Wine Grapes. Am. J. Enol. Vitic. 22, 87–91.

[B185] Olmo H. P. (1979). “Grapes. In Evolution of Crop Plants,” (London: Simmonds).

[B186] Onesti G. (2015). Studies on inoculum dynamics of Guignardia bidwellii, causal agent of grape black-rot. Available at: http://hdl.handle.net/10280/10799 10.1094/PHYTO-07-16-0255-R 27726499

[B187] Onesti G. González-Domínguez E. Manstretta V. Rossi V. (2018). Release of Guignardia bidwellii ascospores and conidia from overwintered grape berry mummies in the vineyard. Aust. J. Grape Wine Res. 24, 136–144. 10.1111/ajgw.12321

[B188] Onesti G. González-Domínguez E. Rossi V. (2016). Accurate prediction of black rot epidemics in vineyards using a weather-driven disease model. Pest Manage. Sci. 72, 2321–2329. 10.1002/ps.4277 26996951

[B189] Onesti G. González-Domínguez E. Rossi V. (2017a). Production of Guignardia bidwellii conidia on grape leaf lesions is influenced by repeated washing events and by alternation of dry and wet periods. Eur. J. Plant Pathol. 147, 949–953. 10.1007/s10658-016-1052-1

[B190] Onesti G. González-Domínguez E. Rossi V. (2017b). Production of Pycnidia and Conidia by Guignardia bidwellii, the causal agent of grape black rot, as affected by temperature and humidity. Phytopathology 107, 173–183. 10.1094/PHYTO-07-16-0255-R 27726499

[B191] Pandey S. P. Somssich I. E. (2009). The Role of WRKY transcription factors in plant immunity. Plant Physiol. 150, 1648–1655. 10.1104/pp.109.138990 19420325 PMC2719123

[B192] Pap D. Riaz S. Dry I. B. Jermakow A. Tenscher A. C. Cantu D. (2016). Identification of two novel powdery mildew resistance loci, Ren6 and Ren7, from the wild Chinese grape species Vitis piasezkii. BMC Plant Biol. 16, 170. 10.1186/s12870-016-0855-8 27473850 PMC4966781

[B193] Parage C. Tavares R. Réty S. Baltenweck-Guyot R. Poutaraud A. Renault L. (2012). Structural, functional, and evolutionary analysis of the unusually large stilbene synthase gene family in grapevine. Plant Physiol. 160, 1407–1419. 10.1104/pp.112.202705 22961129 PMC3490603

[B194] Pauquet J. Bouquet A. This P. Adam-Blondon A.-F. (2001). Establishment of a local map of AFLP markers around the powdery mildew resistance gene Run1 in grapevine and assessment of their usefulness for marker assisted selection. Theor. Appl. Genet. 103, 1201–1210. 10.1007/s001220100664

[B195] Pearson R. C. Gadoury D. M. (1992). “Powdery Mildew of Grapes,” in Plant diseases of international importance. Vol III. Diseases of fruit crops. Eds. Kumar J. Chaube H. S. Singh U. S. Mukhopadhyay A. N. (Englewood Cliffs, USA: Prentice Hall), 129–146.

[B196] Penet L. Briand S. Petro D. Bussière F. Guyader S. (2017). Data on microsatellite markers in Colletotrichum gloeosporioides s.l., polymorphism levels and diversity range. Data Br. 12, 644–648. 10.1016/j.dib.2017.05.012 PMC5432675 28540357

[B197] Péros J. P. Troulet C. Guerriero M. Michel-Romiti C. Notteghem J.-L. (2005). Genetic variation and population structure of the grape powdery mildew fungus, Erysiphe necator, in Southern France. Eur. J. Plant Pathol. 113, 407–416. 10.1007/s10658-005-4563-8

[B198] Péros J. P. Michel-Romiti C. Troulet C. Notteghem J. L. (2006a). New rapid PCR protocols to distinguish genetic groups in Erysiphe necator. Vitis - J. Grapevine Res. 45, 47–48.

[B199] Péros J. P. Nguyen T. H. Troulet C. Michel-Romiti C. Notteghem J. L. (2006b). Assessment of powdery mildew resistance of grape and Erysiphe necator pathogenicity using a laboratory assay. Vitis - J. Grapevine Res. 45, 29–36.

[B200] Pertot I. Caffi T. Rossi V. Mugnai L. Hoffmann C. Grando M. S. (2017). A critical review of plant protection tools for reducing pesticide use on grapevine and new perspectives for the implementation of IPM in viticulture. Crop Prot. 97, 70–84. 10.1016/j.cropro.2016.11.025

[B201] Pessina S. Lenzi L. Perazzolli M. Campa M. Dalla Costa L. Urso S. (2016). Knockdown of MLO genes reduces susceptibility to powdery mildew in grapevine. Hortic. Res. 3, 16016. 10.1038/hortres.2016.16 27390621 PMC4935963

[B202] Phukan U. J. Jeena G. S. Shukla R. K. (2016). WRKY transcription factors: molecular regulation and stress responses in plants. Front. Plant Sci. 7, 760. 10.3389/fpls.2016.00760 27375634 PMC4891567

[B203] Pike S. Gao F. Kim M. J. Kim S. H. Schachtman D. P. Gassmann W. (2014). Members of the NPF3 transporter subfamily encode pathogen-inducible nitrate/nitrite transporters in grapevine and Arabidopsis. Plant Cell Physiol. 55, 162–170. 10.1093/pcp/pct167 24259683

[B204] Polonio Á. Pineda M. Bautista R. Martínez-Cruz J. Pérez-Bueno M. L. Barón M. (2019). RNA-seq analysis and fluorescence imaging of melon powdery mildew disease reveal an orchestrated reprogramming of host physiology. Sci. Rep. 9, 7978. 10.1038/s41598-019-44443-5 31138852 PMC6538759

[B205] Poolsawat O. Mahanil S. Laosuwan P. Wongkaew S. Tharapreuksapong A. Reisch B. (2013). Inheritance of downy mildew (Plasmopara viticola) and anthracnose (Sphaceloma ampelinum) resistance in grapevines. Genet. Mol. Res. 12, 6752–6761. 10.4238/2013.December.13.8 24391016

[B206] Poolsawat O. Tharapreuksapong A. Wongkaew S. Chaowiset W. Tantasawat P. (2012). Laboratory and field evaluations of resistance to Sphaceloma ampelinum causing anthracnose in grapevine. Australas. Plant Pathol. 41, 263–269. 10.1007/s13313-012-0127-5

[B207] Poolsawat O. Tharapreuksapong A. Wongkaew S. Reisch B. Tantasawat P. (2010). Genetic diversity and pathogenicity analysis of Sphaceloma ampelinum causing grape anthracnose in Thailand. J. Phytopathol. 158, 837–840. 10.1111/j.1439-0434.2010.01696.x

[B208] Poolsawat O. Tharapreuksapong A. Wongkaew S. Tantasawat P. (2009). Cultural characteristics of Sphaceloma ampelinum, causal pathogen of grape anthracnose on different media. Suranaree J. Sci. Technol 16, 149–157.

[B209] Populer C. (1978). “Changes in host susceptibility with time,” in Plant Disease, An Advanced Treatise. Vol. II. Hous Disease develops in Populations. Eds. Horsfall, and Cowling (New York, USA: Academic Press).

[B210] Qiu W. Feechan A. Dry I. (2015). Current understanding of grapevine defense mechanisms against the biotrophic fungus (Erysiphe necator), the causal agent of powdery mildew disease. Hortic. Res. 2, 15020. 10.1038/hortres.2015.20 26504571 PMC4595975

[B211] Ramming D. W. Gabler F. Smilanick J. Cadle-Davidson M. Barba P. Mahanil S. (2011). A single dominant locus, Ren4, confers rapid non-race-specific resistance to grapevine powdery mildew. Phytopathology 101, 502–508. 10.1094/PHYTO-09-10-0237 21091183

[B212] Ramming D. W. Gabler F. Smilanick J. L. Margosan D. A. Cadle-Davidson M. Barba P. (2012). Identification of race-specific resistance in North American Vitis spp. Phytopathology 102, 83–93. 10.1094/PHYTO-03-11-0062 22165984

[B213] Ramsdell D. C. Milholland R. D. (1988). “Black rot,” in Compendium of Grape Diseases. Eds. Pearson R. C. Goheen A. C. (St. Paul, MN, USA: APS Press), 15–17.

[B214] Rex F. Fechter I. Hausmann L. Töpfer R. (2014). QTL mapping of black rot (Guignardia bidwellii) resistance in the grapevine rootstock “Börner” (V. Theor. Appl. Genet. 127, 1667–1677. 10.1007/s00122-014-2329-4 24865508

[B215] Riaz S. Tenscher A. C. Ramming D. W. Walker M. A. (2011). Using a limited mapping strategy to identify major QTLs for resistance to grapevine powdery mildew (Erysiphe necator) and their use in marker-assisted breeding. Theor. Appl. Genet. 122, 1059–1073. 10.1007/s00122-010-1511-6 21188350 PMC3056998

[B216] Rinaldi P. A. Paffetti D. Comparini C. Broggini G. A. L. Gessler C. Mugnai L. (2017). Genetic variability of Phyllosticta ampelicida, the agent of black rot disease of grapevine. Phytopathology 107, 1406–1416. 10.1094/PHYTO-11-16-0404-R 28569125

[B217] Rossi V. Onesti G. Legler S. E. Caffi T. (2015). Use of systems analysis to develop plant disease models based on literature data: grape black-rot as a case-study. Eur. J. Plant Pathol. 141, 427–444. 10.1007/s10658-014-0553-z

[B218] Roznik D. Hoffmann S. Kozma P. (2017). Screening a large set of grape accessions for resistance against black rot (Guignardia bidwellii/(Ell.)). Mitteilungen Klosterneuburg Rebe und Wein Obs. und Früchteverwertung 67, 149–157.

[B219] Saenz G. S. Taylor J. W. (1999). Phylogeny of the Erysiphales (powdery mildews) inferred from internal transcribed spacer ribosomal DNA sequences. Can. J. Bot. 77, 150–168. 10.1139/b98-235

[B220] Salinari F. Giosuè S. Tubiello F. N. Rettori A. Rossi V. Spanna F. (2006). Downy mildew (Plasmopara viticola) epidemics on grapevine under climate change. Glob. Chang. Biol. 12, 1299–1307. 10.1111/j.1365-2486.2006.01175.x

[B221] Santos R. F. Spósito M. B. Ayres M. R. Sosnowski M. R. (2018a). Phylogeny, morphology and pathogenicity of Elsinoë ampelina, the causal agent of grapevine anthracnose in Brazil and Australia. J. Phytopathol. 166, 187–198. 10.1111/jph.12675

[B222] Santos R. F. Spósito M. B. Ayres M. R. Sosnowski M. (2018b). In vitro production of conidia of Elsinoë ampelina, the causal fungus of grapevine anthracnose. Eur. J. Plant Pathol. 152, 815–821. 10.1007/s10658-018-1502-z

[B223] Savocchia S. Stummer B. Scott E. Wicks T. (1999). Detection of DMI resistance among populations of powdery mildew fungus. Aust. Grapegrow. Winemak., 429, 39–41.

[B224] Sawant I. S. Narkar S. P. Shetty D. S. Upadhyay A. Sawant S. D. (2012). Emergence of Colletotrichum gloeosporioides sensu lato as the dominant pathogen of anthracnose disease of grapes in India as evidenced by cultural, morphological and molecular data. Australas. Plant Pathol. 41, 493–504. 10.1007/s13313-012-0143-5

[B225] Sawant I. S. Wadkar P. N. Rajguru Y. R. Mhaske N. H. Salunkhe V. P. Sawant S. D. (2016). Biocontrol potential of two novel grapevine associated Bacillus strains for management of anthracnose disease caused by Colletotrichum gloeosporioides. Biocontrol Sci. Technol. 26, 964–979. 10.1080/09583157.2016.1174770

[B226] Schnabel G. Jones A. L. (2001). The 14α-Demethylase (CYP51A1) gene is overexpressed in Venturia inaequalis strains resistant to myclobutanil. Phytopathology 91, 102–110. 10.1094/PHYTO.2001.91.1.102 18944284

[B227] Schnee S. Viret O. Gindro K. (2008). Role of stilbenes in the resistance of grapevine to powdery mildew. Physiol. Mol. Plant Pathol. 72, 128–133. 10.1016/j.pmpp.2008.07.002

[B228] Schoch C. L. Seifert K. A. Huhndorf S. Robert V. Spouge J. L. Levesque C. A. (2012). Nuclear ribosomal internal transcribed spacer (ITS) region as a universal DNA barcode marker for Fungi. Proc. Natl. Acad. Sci. U. S. A. 109, 6241–6246. 10.1073/pnas.1117018109 22454494 PMC3341068

[B229] Seifert K. A. (2009). Progress towards DNA barcoding of fungi. Mol. Ecol. Resour. 9, 83–89. 10.1111/j.1755-0998.2009.02635.x 21564968

[B230] Shi J. He M. Cao J. Wang H. Ding J. Jiao Y. (2014). The comparative analysis of the potential relationship between resveratrol and stilbene synthase gene family in the development stages of grapes (Vitis quinquangularis and Vitis vinifera). Plant Physiol. Biochem. PPB 74, 24–32. 10.1016/j.plaphy.2013.10.021 24246671

[B231] Snyder C. (2018). Comparative transcriptomics of grapevine powdery mildew. Thesis. Available at: http://scholarworks.rit.edu/theses/9767

[B232] Sombardier A. Dufour M. Blancard D. Corio-Costet M. (2010). Sensitivity of Podosphaera aphanis isolates to DMI fungicides: distribution and reduced cross-sensitivity. Pest Manage. Sci. Former. Pestic. Sci. 66, 35–43. 10.1002/ps.1827 19728323

[B233] Soytong K. Srinon W. Rattanacherdchai K. Kanokmedhakul S. Kanokmedhakul K. (2005). Application of antagonistic fungi to control anthracnose disease of grape. J. Agric. Technol. 33, 33–41.

[B234] Spanu P. D. Abbott J. C. Amselem J. Burgis T. A. Soanes D. M. Stüber K. (2010). Genome Expansion and Gene Loss in Powdery Mildew Fungi Reveal Tradeoffs in Extreme Parasitism. Sci. (80-.). 330, 1543–1546. 10.1126/science.1194573 21148392

[B235] Spotts R. A. (1976). The effect of temperature and leaf wetness duration on grape black rot infection. Proc. Am. Phytopathol. Soc 3, 373.

[B236] Spotts R. A. (1977). Effect of leaf wetness duration and temperature on the infectivity of Guignardia bidwellii on grape leaves. Phytopathology 67, 1378–1381. 10.1094/phyto-67-1378

[B237] Spotts R. A. (1980). Infection of grape by Guignardia bidwellii - factors affecting lesion development, conidial dispersal, and conidial populations on leaves. Phytopathology 70, 252–255. 10.1094/phyto-70-252

[B238] Staudt G. (1997). Evaluation of resistance to grapevine powdery mildew (Uncinula necator [Scxw.] BURR. Vitis 36, 151–154.

[B239] Stergiopoulos I. Van Nistelrooy J. G. M. Kema G. H. J. De Waard M. A. (2003). Multiple mechanisms account for variation in base-line sensitivity to azole fungicides in field isolates of Mycosphaerella graminicola. Pest Manage. Sci. 59, 1333–1343. 10.1002/ps.766 14667055

[B240] Steva H. Cazenave C. (1996). Evolution of grape powdery mildew insensitivity to DMI fungicides. in “Proceedings of the Brighton Crop Protection Conference, Pests Diseases”, (British Crop Protection Council) 725–730.

[B241] Stummer B. E. Zanker T. Scott E. S. Whisson D. L. (2000). Genetic diversity in populations of Uncinula necator: comparison of RFLP- and PCR-based approaches. Mycol. Res. 104, 44–52. 10.1017/S0953756299001070

[B242] Stuthman D. D. Leonard K. J. Miller-Garvin J. (2007). Breeding crops for durable resistance to disease. Adv. Agron. 95, 319–367. 10.1016/S0065-2113(07)95004-X

[B243] Tantasawat P. A. P. Poolsawat O. Prajongjai T. Chaowiset W. Tharapreuksapong A. (2012). Association of RGA-SSCP markers with resistance to downy mildew and anthracnose in grapevines. Genet. Mol. Res. 11, 1799–1809. 10.4238/2012.July.2.1 22869536

[B244] Teh S. L. Fresnedo-Ramírez J. Clark M. D. Gadoury D. M. Sun Q. Cadle-Davidson L. (2017). Genetic dissection of powdery mildew resistance in interspecific half-sib grapevine families using SNP-based maps. Mol. Breed. 37, 1. 10.1007/s11032-016-0586-4 28127252 PMC5226326

[B245] Tharapreuksapong A. Poolsawat O. Jenweerawat S. Wongkaew S. Thipyapong P. (2009). Molecular, morphological, and pathogenicity characterization of Sphaceloma ampelinum isolates from Thailand. Acta Hortic. 827, 611–618. 10.17660/ActaHortic.2009.827.107

[B246] Thind T. S. Clerjeau M. Olivier J. M. (1986). “First observations on resistance in Venturia inaequalis and Guignardia bidwellii to ergosterol-biosynthesis inhibitors in France,” in 1986 British Crop Protection Conference. Pests and Diseases, Vol. 2 (British Crop Protection Council), 491–498.

[B247] Tian X. Zhang L. Feng S. Zhao Z. Wang X. Gao H. (2019). Transcriptome analysis of apple leaves in response to powdery mildew (Podosphaera leucotricha) infection. Int. J. Mol. Sci. 20. 10.3390/ijms20092326 PMC6539105 31083412

[B248] Toffolatti S. L. De Lorenzis G. Costa A. Maddalena G. Passera A. Bonza M. C. (2018). Unique resistance traits against downy mildew from the center of origin of grapevine (Vitis vinifera). Sci. Rep. 8, 1–11. 10.1038/s41598-018-30413-w 30131589 PMC6104083

[B249] Tomoiaga L. Comsa M. (2010). The strategy of optimization for combat the black rot of vine (Giugnardia bidwelli), in the ecoclimatice conditions from vineyard Tarnave in “Bulletin Of University Of Agricultural Sciences And Veterinary Medicine Cluj-Napoca. Horticulture”, 67, 500–500.

[B250] Töpfer R. Eibach R. (2016). Breeding the next-generation disease-resistant grapevine varieties. Wine Vitic. J. 41, 47.

[B251] Töpfer R. Hausmann L. Harst M. Maul E. Zyprian E. Eibach R. (2011). New Horizons for Grapevine Breeding. Fruit Veg. Cereal Sci. Biotechnol. 5, 79–100.

[B252] Töpfer R. Trapp O. Rist F. Herzog K. Hausmann L. Eibach R. (2018). “Calardis blanc ‘ a new grapevine variety with combined resistances against downy mildew, high resistance against black rot, and high botrytis resilience” in Book of Abstracts of 12th International conference on grapevine breeding and genetics. (Bordeaux, France), 256. Available at: http://gbg2018.u-bordeaux.fr/files/gbg2018/presentation/O17_20180705_ GBGB_Bordeaux.pdf

[B253] Toth Z. Winterhagen P. Kalapos B. Su Y. Kovacs L. Kiss E. (2016). Expression of a grapevine NAC transcription factor gene is induced in response to powdery mildew colonization in salicylic acid-independent manner. Sci. Rep. 6, 30825. 10.1038/srep30825 27488171 PMC4973223

[B254] Ullrich C. C. I. Kleespies R. R. G. Enders M. Koch E. (2009). Biology of the black rot pathogen, Guignardia bidwellii, its development in susceptible leaves of grapevine Vitis vinifera. J. Für Kult. 61, 82–90.

[B255] van Heerden C. J. Burger P. Vermeulen A. Prins R. (2014). Detection of downy and powdery mildew resistance QTL in a ‘Regent’ × ‘RedGlobe’ population. Euphytica 200, 281–295. 10.1007/s10681-014-1167-4

[B256] Vanacker H. Carver T. L. W. Foyer C. H. (2000). Early H(2)O(2) accumulation in mesophyll cells leads to induction of glutathione during the hyper-sensitive response in the barley-powdery mildew interaction. Plant Physiol. 123, 1289–1300. 10.1104/pp.123.4.1289 10938348 PMC59088

[B257] Vannozzi A. Dry I. B. Fasoli M. Zenoni S. Lucchin M. (2012). Genome-wide analysis of the grapevine stilbene synthase multigenic family: genomic organization and expression profiles upon biotic and abiotic stresses. BMC Plant Biol. 12, 130. 10.1186/1471-2229-12-130 22863370 PMC3433347

[B258] Vasanthaiah H. K. N. Basha S. M. Katam R. (2010). Differential expression of chitinase and stilbene synthase genes in Florida hybrid bunch grapes to Elsinoë ampelina infection. Plant Growth Regul. 61, 127–134. 10.1007/s10725-010-9458-9

[B259] Vasanthaiah H. K. N. N. Katam R. Basha S. M. (2009). Characterization of unique and differentially expressed proteins in anthracnose-tolerant florida hybrid bunch grapes. Appl. Biochem. Biotechnol. 157, 395–406. 10.1007/s12010-008-8380-3 18931950

[B260] Venturini L. Ferrarini A. Zenoni S. Tornielli G. B. Fasoli M. Dal Santo S. (2013). De novo transcriptome characterization of Vitis vinifera cv. BMC Genomics 14, 41. 10.1186/1471-2164-14-41 23331995 PMC3556335

[B261] Vezzulli S. Zulini L. Stefanini M. (2019). Genetics-assisted breeding for downy/powdery mildew and phylloxera resistance at FEM. BIO Web Conf. 12, 01020. 10.1051/bioconf/20191201020

[B262] Wakefield L. Gadoury D. M. Seem R. C. Milgroom M. G. Sun Q. Cadle-Davidson L. (2011). Differential gene expression during conidiation in the grape powdery mildew pathogen, Erysiphe necator. Phytopathology 101, 839–846. 10.1094/PHYTO-11-10-0295 21405992

[B263] Wang J. Yao W. Wang L. Ma F. Tong W. Wang C. (2017a). Overexpression of VpEIFP1, a novel F-box/Kelch-repeat protein from wild Chinese Vitis pseudoreticulata, confers higher tolerance to powdery mildew by inducing thioredoxin z proteolysis. Plant Sci. 263, 142–155. 10.1016/j.plantsci.2017.07.004 28818370

[B264] Wang L. Xie X. Yao W. Wang J. Ma F. Wang C. (2017b). RING-H2-type E3 gene VpRH2 from Vitis pseudoreticulata improves resistance to powdery mildew by interacting with VpGRP2A. J. Exp. Bot. 68, 1669–1687. 10.1093/jxb/erx033 28369599

[B265] Wang M. Vannozzi A. Wang G. Liang Y.-H. Tornielli G. B. Zenoni S. (2014). Genome and transcriptome analysis of the grapevine (Vitis vinifera L.) WRKY gene family. Hortic. Res. 1, 14016. 10.1038/hortres.2014.16 26504535 PMC4596322

[B266] Wang X. Wang Y. Hao W. (2007). cDNA cloning and characterization of the novel genes related to aldehyde dehydrogenase from wild Chinese grape (Vitis pseudoreticulata W. DNA Seq. 18, 9–18. 10.1080/10425170600724618 17364808

[B267] Wang X. X. Guo R. Tu M. Wang D. Guo C. Wan R. (2017c). Ectopic expression of the Wild Grape WRKY transcription factor VqWRKY52 in Arabidopsis thaliana enhances resistance to the Biotrophic pathogen powdery Mildew but not to the Necrotrophic pathogen Botrytis cinerea. Front. Plant Sci. 8, 97. 10.3389/fpls.2017.00097 28197166 PMC5281567

[B268] Wang Y. Liu Y. He P. Chen J. Lamikanra O. Lu J. (1995). Evaluation of foliar resistance to Uncinula necator in Chinese wild Vitis spp. Vitis 34, 159–164.

[B269] Wang Y. Liu Y. He P. Lamikanra O. Lu J. (1998). Resistance of Chinese Vitis species to Elsinoë ampelina (de Bary) Shear. HortScience 33, 123–126. 10.21273/HORTSCI.33.1.123

[B270] Wang Y. Wang D. Wang F. Huang L. Tian X. van Nocker S. (2017d). Expression of the Grape VaSTS19 Gene in Arabidopsis improves resistance to powdery Mildew and Botrytis cinerea but increases susceptibility to Pseudomonas syringe pv Tomato DC3000. Int. J. Mol. Sci. 18, 2000. 10.3390/ijms18092000 PMC5618649 28926983

[B271] Welter L. J. Göktürk-Baydar N. Akkurt M. Maul E. Eibach R. Töpfer R. (2007). Genetic mapping and localization of quantitative trait loci affecting fungal disease resistance and leaf morphology in grapevine (Vitis vinifera L). Mol. Breed. 20, 359–374. 10.1007/s11032-007-9097-7

[B272] Wen Y. Wang X. Xiao S. Wang Y. (2012). Ectopic expression of VpALDH2B4, a novel aldehyde dehydrogenase gene from Chinese wild grapevine (Vitis pseudoreticulata), enhances resistance to mildew pathogens and salt stress in Arabidopsis. Planta 236, 525–539. 10.1007/s00425-012-1624-z 22437646

[B273] Wen Z. Yao L. Singer S. D. Muhammad H. Li Z. Wang X. (2017). Constitutive heterologous overexpression of a TIR-NB-ARC-LRR gene encoding a putative disease resistance protein from wild Chinese Vitis pseudoreticulata in Arabidopsis and tobacco enhances resistance to phytopathogenic fungi and bacteria. Plant Physiol. Biochem. 112, 346–361. 10.1016/j.plaphy.2017.01.017 28131063

[B274] Wen Z. Yao L. Wan R. Li Z. Liu C. Wang X. (2015). Ectopic expression in Arabidopsis thaliana of an NB-ARC encoding putative disease resistance gene from wild Chinese Vitis pseudoreticulata enhances resistance to Phytopathogenic fungi and bacteria. Front. Plant Sci. 6, 1087. 10.3389/fpls.2015.01087 26697041 PMC4674559

[B275] Weng K. Li Z.-Q. Liu R.-Q. Wang L. Wang Y.-J. Xu Y. (2014). Transcriptome of Erysiphe necator-infected Vitis pseudoreticulata leaves provides insight into grapevine resistance to powdery mildew. Hortic. Res. 1, 14049. 10.1038/hortres.2014.49 26504551 PMC4596327

[B276] Wicht B. Petrini O. Jermini M. Gessler C. Broggini G. A. L. (2012). Molecular, proteomic and morphological characterization of the ascomycete Guignardia bidwellii, agent of grape black rot: a polyphasic approach to fungal identification. Mycologia 104, 1036–1045. 10.3852/11-242 22492405

[B277] Wicker T. Oberhaensli S. Parlange F. Buchmann J. P. Shatalina M. Roffler S. (2013). The wheat powdery mildew genome shows the unique evolution of an obligate biotroph. Nat. Genet. 45, 1092–1096. 10.1038/ng.2704 23852167

[B278] Wilcox W. F. (2000). Evaluation of fungicide programs for control of black rot of grapes, 1999. Fungic. Nematic. Tests 55, 118.

[B279] Wilcox W. F. (2003). Black rot. Disease Identification Sheet No. 102GFSG-D4. New York State IPM Program Available at: https://ecommons.cornell.edu/handle/1813/43076

[B280] Wilcox W. F. (2005). Occurrence and management of QoI fungicide resistance in grape vineyards. Phytopathology 95, S143.

[B281] Wilcox W. F. Gubler W. D. Uyemoto J. K. (2017). “PART I: Diseases Caused by Biotic Factors,” in Compendium of Grape Diseases, Disorders, and Pests, Second Edition, Second Printing. Eds. Wilcox W. F. Gubler W. D. Uyemoto J. K. (St. Paul, Minnesota 55121, USA: The American Phytopathological Society), 17–146. 10.1094/9780890544815.002

[B282] Wilcox W. F. Riegel D. G. (1996). Evaluation of fungicides and spray timings for control of black rot and downy mildew of grapes, 1995. Fungic. Nematic. Tests 51, 74–75.

[B283] Wilcox W. F. Riegel D. G. (1997). Evaluation of fungicide programs for control of black rot of grapes, 1996. Fungic. Nematic. Tests 52, 874.

[B284] Wilcox W. F. Riegel D. G. Emele L. R. (1999). Evaluation of fungicide programs for control of grape black rot, 1998. Fungic. Nematic. Tests 54, 110.

[B285] Wilkinson S. W. Magerøy M. H. Sánchez A. L. Smith L. M. Furci L. Cotton T. E. A. (2019). Surviving in a hostile world: plant strategies to resist pests and diseases. Annu. Rev. Phytopathol. 57, 505–529. 10.1146/annurev-phyto-082718-095959 31470772

[B286] Wyand R. A. Brown J. K. M. (2005). Sequence variation in the CYP51 gene of Blumeria graminis associated with resistance to sterol demethylase inhibiting fungicides. Fungal Genet. Biol. 42, 726–735. 10.1016/j.fgb.2005.04.007 15916909

[B287] Xie X. Wang Y. (2016). VqDUF642, a gene isolated from the Chinese grape Vitis quinquangularis, is involved in berry development and pathogen resistance. Planta 244, 1075–1094. 10.1007/s00425-016-2569-4 27424038

[B288] Xu L. Zhu L. Tu L. Liu L. Yuan D. Jin L. (2011a). Lignin metabolism has a central role in the resistance of cotton to the wilt fungus Verticillium dahliae as revealed by RNA-Seq-dependent transcriptional analysis and histochemistry. J. Exp. Bot. 62, 5607–5621. 10.1093/jxb/err245 21862479 PMC3223054

[B289] Xu T.-F. Zhao X.-C. Jiao Y.-T. Wei J.-Y. Wang L. Xu Y. (2014). A pathogenesis related protein, VpPR-10.1, from Vitis pseudoreticulata: an insight of its mode of antifungal activity. PloS One 9, e95102. 10.1371/journal.pone.0095102 24759805 PMC3997386

[B290] Xu W. Yu Y. Ding J. Hua Z. Wang Y. (2010a). Characterization of a novel stilbene synthase promoter involved in pathogen- and stress-inducible expression from Chinese wild Vitis pseudoreticulata. Planta 231, 475–487. 10.1007/s00425-009-1062-8 19937257

[B291] Xu W. Yu Y. Zhou Q. Ding J. Dai L. Xie X. (2011b). Expression pattern, genomic structure, and promoter analysis of the gene encoding stilbene synthase from Chinese wild Vitis pseudoreticulata. J. Exp. Bot. 62, 2745–2761. 10.1093/jxb/erq447 21504880

[B292] Xu Y. Yu H. He M. Yang Y. Wang Y. (2010b). Isolation and expression analysis of a novel pathogenesis-related protein 10 gene from Chinese wild Vitis pseudoreticulata induced by Uncinula necator. Biologia (Bratisl) 65, 653–659. 10.2478/s11756-010-0056-0

[B293] Xu Y. Zhu Z. Xiao Y. Wang Y. (2009). Construction of a cDNA library of Vitis pseudoreticulata native to China inoculated with Uncinula necator and the analysis of potential defence-related expressed sequence tags (ESTs). South Afr. J. Enol. Vitic. 30, 65–71.

[B294] Yamamoto T. Iketani H. Ieki H. Nishizawa Y. Notsuka K. Hibi T. (2000). Transgenic grapevine plants expressing a rice chitinase with enhanced resistance to fungal pathogens. Plant Cell Rep. 19, 639–646. 10.1007/s002999900174 30754799

[B295] Yan J.-Y. Jayawardena M. M. R. S. R. S. Goonasekara I. D. Wang Y. Zhang W. Liu M. (2015). Diverse species of Colletotrichum associated with grapevine anthracnose in China. Fungal Divers. 71, 233–246. 10.1007/s13225-014-0310-9

[B296] Yan X. Qiao H. Zhang X. Guo C. Wang M. Wang Y. (2017). Analysis of the grape (Vitis vinifera L.) thaumatin-like protein (TLP) gene family and demonstration that TLP29 contributes to disease resistance. Sci. Rep. 7, 4269. 10.1038/s41598-017-04105-w 28655869 PMC5487326

[B297] Ypema H. L. Ypema M. Gubler W. D. (1997). Sensitivity of Uncinula necator to benomyl, triadimefon, myclobutanil, and fenarimol in California. Plant Dis. 81, 293–297. 10.1094/PDIS.1997.81.3.293 30861773

[B298] Yu Y. Xu W. Wang J. Wang L. Yao W. Xu Y. (2013a). A core functional region of the RFP1 promoter from Chinese wild grapevine is activated by powdery mildew pathogen and heat stress. Planta 237, 293–303. 10.1007/s00425-012-1769-9 23053541

[B299] Yu Y. Xu W. Wang J. Wang L. Yao W. Yang Y. (2013b). The Chinese wild grapevine (Vitis pseudoreticulata) E3 ubiquitin ligase Erysiphe necator-induced RING finger protein 1 (EIRP1) activates plant defense responses by inducing proteolysis of the VpWRKY11 transcription factor. New Phytol. 200, 834–846. 10.1111/nph.12418 23905547

[B300] Yu Y. Xu W. Wang S. Xu Y. Li H. Wang Y. (2011). VpRFP1, a novel C4C4-type RING finger protein gene from Chinese wild Vitis pseudoreticulata, functions as a transcriptional activator in defence response of grapevine. J. Exp. Bot. 62, 5671–5682. 10.1093/jxb/err253 21862480 PMC3223060

[B301] Yun H. K. Louime C. Lu J. (2007). First Report of Anthracnose Caused by Elsinoe ampelina on Muscadine Grapes (Vitis rotundifolia) in Northern Florida. Plant Dis. 91, 905–905. 10.1094/PDIS-91-7-0905B 30780405

[B302] Yun H. K. Park K. S. Rho J. H. Choi Y. J. Kang K. K. (2006). Evaluating the resistance of grapevines against anthracnose by pathogen inoculation, vineyard inspection, and bioassay with culture filtrate from Elsinoe ampelina. J. Am. Pomol. Soc 60, 97–103.

[B303] Zapparata A. Da Lio D. Sarrocco S. Vannacci G. Baroncelli R. (2017). First Report of Colletotrichum godetiae Causing Grape (Vitis vinifera) Berry Rot in Italy. Plant Dis. 101, 1051. 10.1094/PDIS-12-16-1764-PDN

[B304] Zendler D. Schneider P. Töpfer R. Zyprian E. (2017). Fine mapping of Ren3 reveals two loci mediating hypersensitive response against Erysiphe necator in grapevine. Euphytica 213, 68. 10.1007/s10681-017-1857-9

[B305] Zhang K. Zhang N. Cai L. (2013). Typification and phylogenetic study of phyllosticta ampelicida and p. Mycologia 105, 1030–1042. 10.3852/12-392 23709479

[B306] Zhu Q. Gao P. Wan Y. Cui H. Fan C. Liu S. (2018). Comparative transcriptome profiling of genes and pathways related to resistance against powdery mildew in two contrasting melon genotypes. Sci. Hortic. (Amsterdam) 227, 169–180. 10.1016/j.scienta.2017.09.033

[B307] Zhu Z. Shi J. Cao J. He M. Wang Y. (2012a). VpWRKY3, a biotic and abiotic stress-related transcription factor from the Chinese wild Vitis pseudoreticulata. Plant Cell Rep. 31, 2109–2120. 10.1007/s00299-012-1321-1 22847334

[B308] Zhu Z. Shi J. He M. Cao J. Wang Y. (2012b). Isolation and functional characterization of a transcription factor VpNAC1 from Chinese wild Vitis pseudoreticulata. Biotechnol. Lett. 34, 1335–1342. 10.1007/s10529-012-0890-y 22391737

[B309] Zhu Z. Shi J. Xu W. Li H. He M. Xu Y. (2013). Three ERF transcription factors from Chinese wild grapevine Vitis pseudoreticulata participate in different biotic and abiotic stress-responsive pathways. J. Plant Physiol. 170, 923–933. 10.1016/j.jplph.2013.01.017 23541511

[B310] Zini E. Dolzani C. Stefanini M. Gratl V. Bettinelli P. Nicolini D. (2019). R-Loci arrangement versus downy and powdery mildew resistance level: a Vitis Hybrid Survey. Int. J. Mol. Sci. 20, 3526. 10.3390/IJMS20143526 PMC6679420 31323823

[B311] Zyprian E. Ochßner I. Schwander F. Šimon S. Hausmann L. Bonow-Rex M. (2016). Quantitative trait loci affecting pathogen resistance and ripening of grapevines. Mol. Genet. Genomics 291, 1573–1594. 10.1007/s00438-016-1200-5 27038830

