# Supplementary material for: Emergent Ascomycetes in Viticulture: An Interdisciplinary Overview
Source: Front Plant Sci. 2019 Nov 22;10:1394. doi: 10.3389/fpls.2019.01394 (PMC6883492; doi:10.3389/fpls.2019.01394)
Supplement: Supplementary file 1 [file DataSheet_1.pdf]

Emergent Ascomycetes in viticulture: an interdisciplinary overview

Pirrello C. [1,2§], Mizzotti C. [3§], Tomazetti T.C. [4§], Colombo M.[1], Bettinelli P.[1], Prodorutti D.[5], Peressotti E.[1], Zulini L.[1], Stefanini M.[1], Angeli G.[5], Masiero S.[3], Welter L.J.[6], Hausmann L.[7], Vezzulli S.[1]*

Supplementary Text 1. Susceptibility of genes associated with PM

Improved efficacy and durability of PM resistance can be enhanced by understanding the genetic basis of resistance and susceptibility. Based on a high-resolution map, Barba et al. (2014) studied the inheritance of *E. necator* resistance and susceptibility of wild *V. rupestris* B38 and cultivated *V. vinifera* 'Chardonnay' finding evidence for quantitative variation. In particular, they identified ten SNPs on chromosome 9 associated with a locus for susceptibility from 'Chardonnay', named *Sen1* (*Susceptibility to E. necator 1*). This finding is a breakthrough towards negative selection among breeding progenies.

In order to dissect PM susceptibility, an alternative approach relies on the biological candidacy of susceptibility (S) genes. Unlike R genes, S genes are required for successful pathogen infection, and thus are considered essential for compatible plant-pathogen interactions. Based on these interactions, three main molecular mechanisms have been associated with S genes: (i) basic compatibility, which assists in host recognition and penetration; (ii) sustained compatibility, which is required for pathogen proliferation and spread; and (iii) negative regulation of immune signals (van Schie and Takken, 2014). While R genes are mostly dominant, the disease resistance provided by manipulation of S genes is mostly recessive and associated with some fitness cost. S gene-mediated resistance can be pathogen specific, when the impaired pathway is implicated in pre-penetration, penetration, or post-penetration requirements of a certain pathogen (Zaidi et al., 2018).

S genes can belong to several families with different roles within the cell; their peculiarity consists of assisting the pathogen in spreading the infection, and, consequently, to hinder the pathogen activity when mutated in homozygosity. Initially discovered as a natural mutation in barley (*Hordeum vulgare*; Jorgensen, 1992), *MLO* (Mildew resistance Locus O) driven-resistance against PM (*Blumeria graminis*) was studied in numerous other plants. Natural *mlo* mutants were identified in cucumber (*Cucumis sativus*), melon (*Cucumis melo*), pea (*Pisum sativum*), tomato (*Solanum lycopersicum*) and tobacco (*Nicotiana tabacum*) (Kusch *et al.*, 2017). Unlike wheat (*Triticum aestivum*) which bears the closest orthologs of barley *MLO,* the number of genes belonging to this family varies a lot within dicots: from 10 to 25 members divided in clades where clade V is the one involved in PM susceptibility. In the last decade, grapevine S genes were studied in more depth. Feechan et al. (2008) defined the grapevine *VvMLO* gene family with 17 putative members belonging to 6 clades. Studies began in *A. thaliana* with *AtMLO2, AtMLO6* and *AtMLO12* and tomato *SlMLO1* genes, which were found to be required for PM susceptibility, culminating in the identification of 7

VvMLO orthologs: *VvMLO1, VvMLO3, VvMLO6, VvMLO7, VvMLO9, VvMLO13* and *VvMLO17,* all belonging to clade V.

As a further confirmation for the key role of such genes in grapevine-*E. necator* interaction, different members of *VvMLO* gene family were found to be induced at transcriptional level upon *E. necator* inoculation: *VvMLO3, VvMLO4*, *VvMLO17* (Feechan et al., 2008) and *VvMLO13*, *VvMLO7* (Winterhagen et al., 2008). Furthermore, exogenous expression of *VvMLO11* and *VvMLO13* showed partial recovery of susceptibility to *E. cichoracearum* in *Arabidopsis mlo2 mlo6 mlo12* triple mutant (Feechan et al., 2013). In contrast, Pessina et al. (2016) proved that *VvMLO7* and *VvMLO6* RNAi silencing gives the most significant response in terms of resistance to *E. necator* in grapevine, even more than *VvMLO11* and *VvMLO13* knock-down. Members of the MLO protein family show a heptahelical transmembrane structure with three extracellular loops at the N-terminus and three intracellular loops at the C-terminus (Devoto et al., 1999, 2003) next to a calmodulin-binding site, which negatively regulates defence mechanisms by the accumulation of cell wall appositions at the *E. necator* penetration site (Kim et al., 2002; Feechan et al., 2008). Other carried out studies on Arabidopsis showed that MLO protein are involved in a number of physiological aspects, such as as root morphogenesis and architecture (Chen et al., 2009), as well as pollen tube reception by the ovary (Kessler et al., 2010). Given this evidence for their biological function, some *VvMLO* genes are being deep-sequenced in a large *Vitis* spp. panel, and thus scouted for their natural variations as novel potential players in grapevine PM resistance breeding (Pirrello et al. 2018).

Disrupting S genes may interfere with the compatibility between the host and the pathogens and consequently provide broad-spectrum and durable disease resistance. In the past, genetic manipulation of such S genes has been shown to confer disease resistance in various economically important crops. Recent studies focused on the use of genome editing to target S genes for the development of transgene-free and durable disease-resistant crop varieties (Zaidi et al. 2018). On this trail, an example of S gene-mediated approach to induce *E. necator* resistance has recently been presented (Giacomelli et al. 2018). In coming years, it is not excluded that S genes associated with the studied ascomycetes will be identified and exploited both in conventional breeding programs and in genome editing strategies.

References

Barba, P., Cadle-Davidson, L., Harriman, J., Glaubitz, J. C., Brooks, S., Hyma, K., et al. (2014). Grapevine powdery mildew resistance and susceptibility loci identified on a high-resolution SNP map. *Theor. Appl. Genet.* 127, 73–84. doi:10.1007/s00122-013-2202-x.

Chen, Z., Noir, S., Kwaaitaal, M., Hartmann, H. A., Wu, M.-J., Mudgil, Y., et al. (2009). Two seven-transmembrane domain MILDEW RESISTANCE LOCUS O proteins cofunction in Arabidopsis root thigmomorphogenesis. *Plant Cell* 21, 1972–91. doi:10.1105/tpc.108.062653.

Devoto, A., Hartmann, H. A., Piffanelli, P., Elliott, C., Simmons, C., Taramino, G., et al. (2003). Molecular phylogeny and evolution of the plant-specific seven-transmembrane MLO family. *J. Mol. Evol.* 56, 77–88. doi:10.1007/s00239-002-2382-5.

Devoto, A., Piffanelli, P., Nilsson, I., Wallin, E., Panstruga, R., von Heijne, G., et al. (1999). Topology, Subcellular Localization, and Sequence Diversity of the Mlo Family in Plants. *J. Biol. Chem.* 274, 34993–35004. doi:10.1074/jbc.274.49.34993.

Feechan, A., Jermakow, A. M., Ivancevic, A., Godfrey, D., Pak, H., Panstruga, R., et al. (2013). Host Cell Entry of Powdery Mildew Is Correlated with Endosomal Transport of Antagonistically Acting VvPEN1 and VvMLO to the Papilla. *Mol. Plant-Microbe Interact.* 26, 1138–1150. doi:10.1094/MPMI-04-13-0091-R.

Feechan, A., Jermakow, A. M., Torregrosa, L., Panstruga, R., and Dry, I. B. (2008). Identification of grapevine MLO gene candidates involved in susceptibility to powdery mildew. *Funct. Plant Biol.* 35, 1255. doi:10.1071/FP08173.

Giacomelli, L., Moser, C., Malnoy, M., van der Voort, JR., Zeilmaker, T., (2018). Generation of mildew-resistant grapevine clones via genome editing. Book of Abstracts of the XII International Conference on Grapevine Breeding and Genetics, Bordeaux, France, 15-20 July 2018.

Kessler, S. A., Shimosato-Asano, H., Keinath, N. F., Wuest, S. E., Ingram, G., Panstruga, R., et al. (2010). Conserved molecular components for pollen tube reception and fungal invasion. *Science* 330, 968–71. doi:10.1126/science.1195211.

Kim, M. C., Panstruga, R., Elliott, C., Müller, J., Devoto, A., Yoon, H. W., et al. (2002). Calmodulin interacts with MLO protein to regulate defence against mildew in barley. *Nature* 416, 447–51. doi:10.1038/416447a.

Kusch, S., and Panstruga, R. (2017). mlo-Based Resistance: An Apparently Universal "Weapon" to Defeat Powdery Mildew Disease. *Mol. Plant. Microbe. Interact.* 30, 179–189. doi:10.1094/MPMI-12-16-0255-CR.

Pessina, S., Lenzi, L., Perazzolli, M., Campa, M., Dalla Costa, L., Urso, S., et al. (2016). Knockdown of MLO genes reduces susceptibility to powdery mildew in grapevine. *Hortic. Res.* 3, 16016. doi:10.1038/hortres.2016.16.

Pirrello, C., Zeilmaker, T., Giacomelli, L., Bianco, L., Moser, C., Vezzulli, S. (2018). Scouting downy and powdery mildew susceptibility genes: a diversity study in *Vitis* spp. Book of Abstracts of the XII International Conference on Grapevine Breeding and Genetics, Bordeaux, France, 15-20 July 2018.

van Schie, C. C. N., and Takken, F. L. W. (2014). Susceptibility Genes 101: How to Be a Good Host. *Annu. Rev. Phytopathol.* 52, 551–581. doi:10.1146/annurev-phyto-102313-045854.

Winterhagen, P., Howard, S. F., Qiu, W., and Kovács, L. G. (2008). Transcriptional up-regulation of grapevine MLO genes in response to powdery mildew infection. *Am. J. Enol. Vitic.* 59, 159–168. Available at: http://www.ajevonline.org/content/59/2/159.long.

Zaidi, S. S. e. A., Mukhtar, M. S., and Mansoor, S. (2018). Genome Editing: Targeting Susceptibility Genes for Plant Disease Resistance. *Trends Biotechnol.* 36, 898–906. doi:10.1016/j.tibtech.2018.04.005.
