# Supplementary material for: Emergent Ascomycetes in Viticulture: An Interdisciplinary Overview
Source: Front Plant Sci. 2019 Nov 22;10:1394. doi: 10.3389/fpls.2019.01394 (PMC6883492; doi:10.3389/fpls.2019.01394)
Supplement: Supplementary file 2 [file Presentation_1.pptx]

## Slide 1
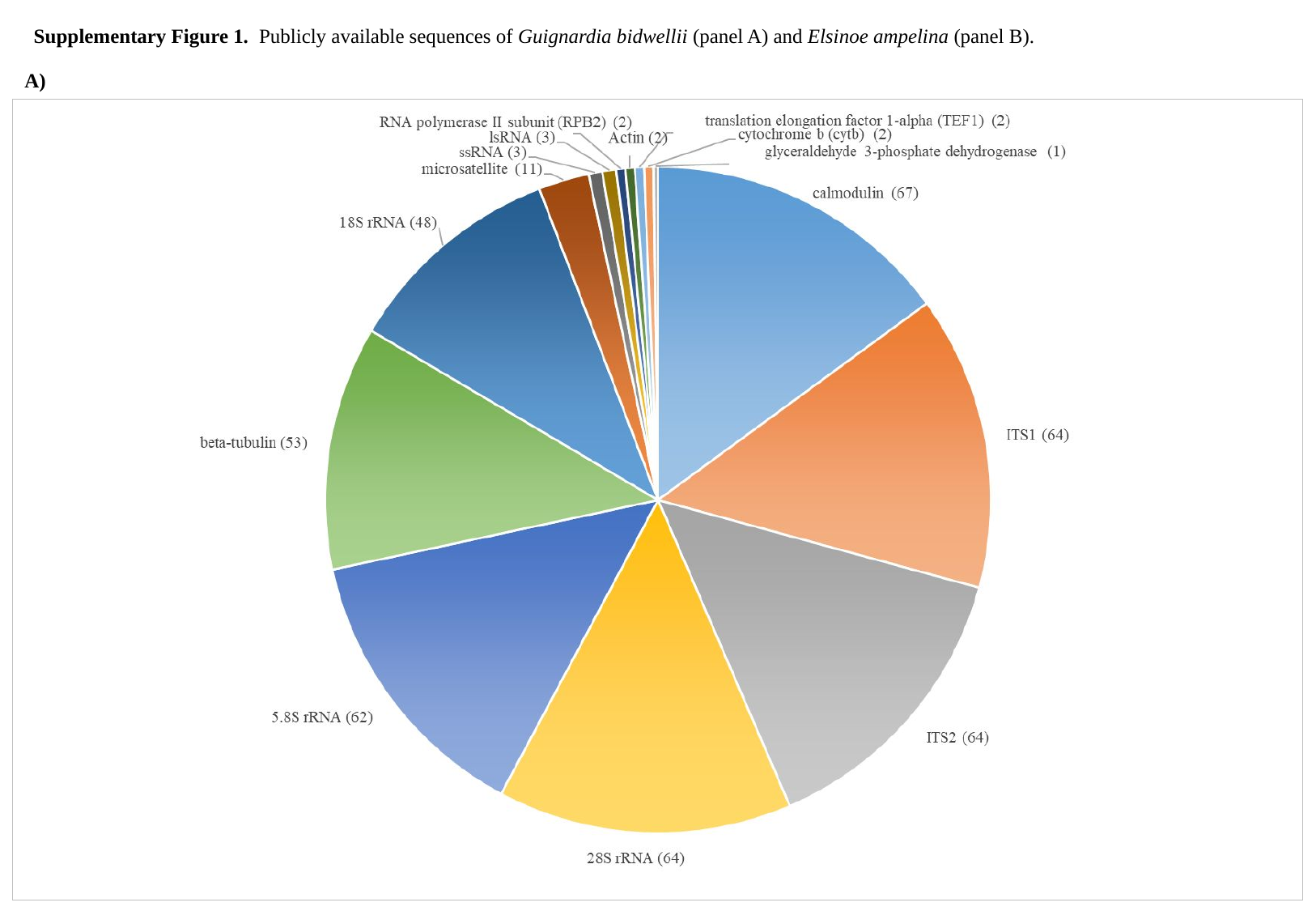

Supplementary Figure 1. Publicly available sequences of Guignardia bidwellii (panel A) and Elsinoe ampelina (panel B).
A)

## Slide 2
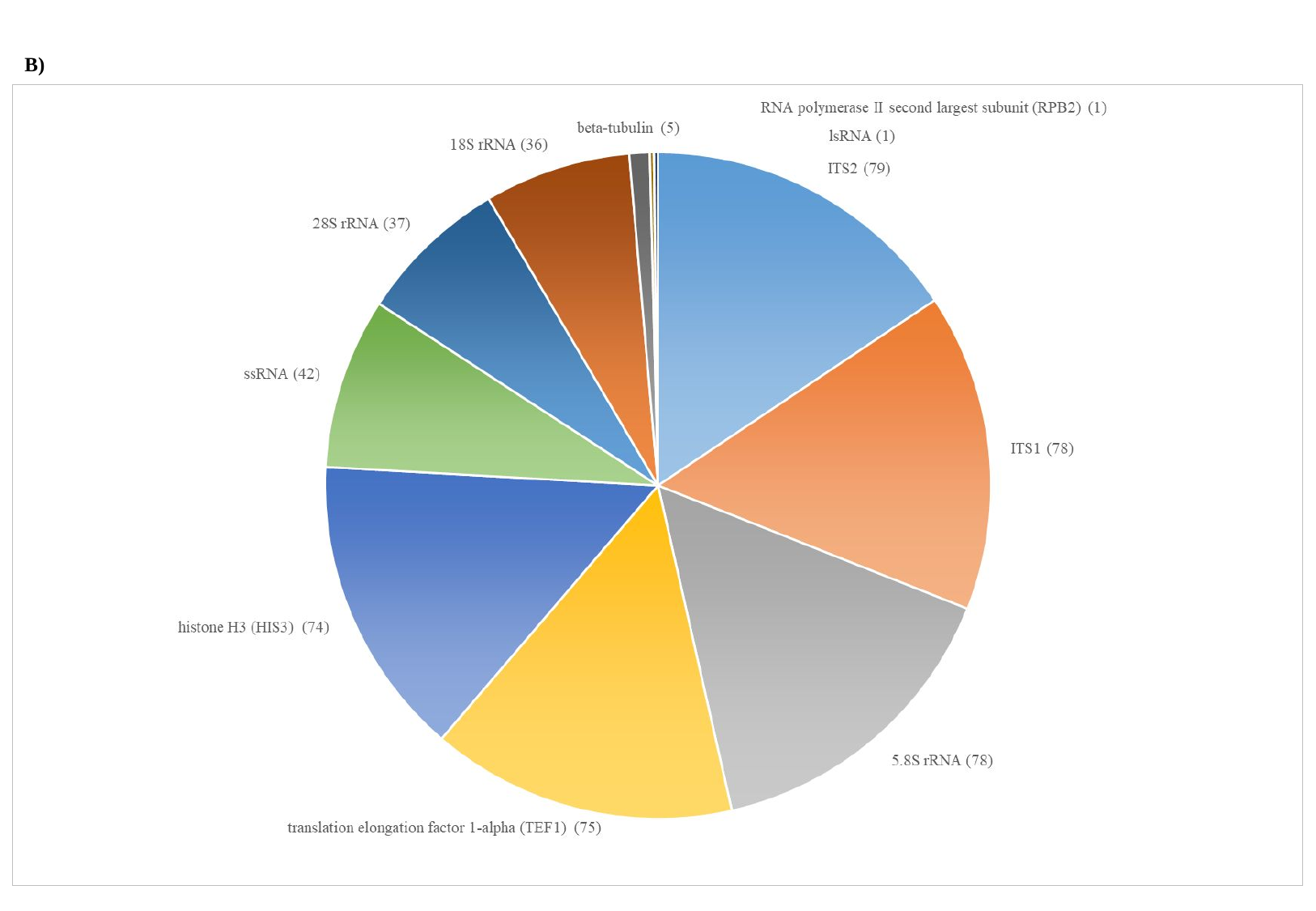

B)
